# Comparative analysis of rumen metagenomes with dietary supplementation of 3-nitrooxypropanol revealed divergent modes of action in hydrogen metabolism and reductant pathways between beef and dairy cattle

**DOI:** 10.1186/s40168-025-02201-y

**Published:** 2026-02-19

**Authors:** Youyoung Choi, Mi Zhou, Masahito Oba, Atmir Romero-Pérez, Karen A. Beauchemin, Stephane Duval, Maik Kindermann, Le Luo Guan

**Affiliations:** 1 Faculty of Land and Food Systems, The University of British Columbia, Vancouver, BC V6T 1Z4 Canada; 2 Department of Agricultural, Food and Nutritional Science, University of Alberta, Edmonton, AB T6G 2P5 Canada; 3 The Research Center for Animal Science, Graduate School of Integrated Sciences for Life, Hiroshima University, Higashi-Hiroshima, 739-8528 Japan; 4 Department of Animal Nutrition and Biochemistry, Faculty of Veterinary Medicine and Animal Science, National Autonomous University of Mexico, Mexico City, 04510 Mexico; 5 Agriculture and Agri-Food Canada, Lethbridge Research and Development Centre, Lethbridge, AB T1J 4B1 Canada; 6 DSM Nutritional Products, Animal Nutrition and Health, Wurmisweg 576, Kaiseraugst, 4303 Switzerland

**Keywords:** 3-Nitrooxypropanol, Beef cattle, Dairy cattle, Metagenomics, Metataxonomics, Comparative analysis

## Abstract

**Background:**

The compound 3-nitrooxypropanol (3-NOP), an inhibitor of methyl-coenzyme M reductase (MCR), reduces enteric methane production in both beef and dairy cattle. Although the proposed mechanisms of 3-NOP involve on inhibiting the activity of MCR in vivo, it is unknown how this process could affect rumen microbiome as a whole and if it differs between beef and dairy cattle. This study conducted a comparative analysis of the rumen microbiome and its functional shifts in four different cattle studies (two beef and two dairy cattle studies) that evaluated 3-NOP supplementation using metataxonomics and metagenomics.

**Results:**

Comparative analysis of 281 rumen metataxonomic datasets (143 beef and 138 dairy cattle) revealed that dietary supplementation with 3-NOP affected rumen bacteria and methanogens. Further, comparative analysis of 54 metagenomic datasets (24 beef and 30 dairy cattle) revealed that 3-NOP inhibited *mcrA*, decreased the abundances of *Methanobrevibacter gottschalkii* and the protozoal species *Isotricha prostoma*, while increased the abundances of *Methanobrevibacter ruminantium* and *Methanosphaera* sp., *Prevotella* sp. was a significant bacterial taxon in both beef and dairy cattle, contributing to various pathways such as propionate and butyrate production. Its increased abundance after 3-NOP supplementation may also be linked to the decrease in *Isotricha prostoma*. Hydrogenotrophic methanogenesis decreased after 3-NOP supplementation with the abundance of genes involved in methylenetetrahydromethanopterin dehydrogenase decreased in beef cattle, while that of 4Fe-4S ferredoxin gene decreased in dairy cattle. The abundance of protozoal *Polyplastron multivesiculatum* increased after long-term 3-NOP supplementation in beef cattle, potentially due to changes in hydrogen (H_2_) partial pressure. During 3-NOP-mediated methanogenesis reduction, abundance of genes encoding methanogenic hydrogenase and H_2_ producing hydrogenase were decreased, while those encoding H_2_ sensory hydrogenase increased. Acyl-CoA dehydrogenase gene involved in propionate and butyrate production pathways increased in both beef and dairy cattle, while nitrite reductase increased specifically in beef cattle, indicating a rise in alternative H_2_ sinks.

Video Abstract

**Conclusion:**

Our findings revealed broad effects of 3-NOP on rumen microbiome and functions in vivo, with varied effects in beef and dairy cattle, which provide mechanistic insights into the supplementation of 3-NOP in both beef and dairy cattle, supporting its more sustainable and effective use in the future.

**Supplementary Information:**

The online version contains supplementary material available at 10.1186/s40168-025-02201-y.

## Background

The recent Intergovernmental Panel on Climate Change (IPCC) 6th Assessment Report [1] claimed that mitigation of enteric methane (CH_4_) emissions could decrease global warming over relatively short timescales due to its short atmospheric lifetime [2, 3]. Recent reports on Agriculture, Forestry, and Other Land Use show that [4] agricultural CH_4_ emission have been steadily rising. According to three major agricultural emissions databases (Emission Database for Global Atmospheric Research [5], Food and Agriculture Organization of the United Nations Statistics Division [6], and United States Environmental Protection Agency [7]), the main source of these emissions is enteric fermentation from ruminants. Methane is a natural byproduct of rumen fermentation, and its release represents a 6.3 ± 2.0% loss of gross energy intake of ruminant animals [8]. Therefore, CH_4_ mitigation in ruminant sector is crucial for emissions reduction among available options in agriculture.

Recent research has revealed the use of 3-nitrooxypropanol (3-NOP) is a promising approach to lower enteric CH_4_ in ruminants. In beef cattle, 3-NOP has been supplemented at levels ranging from 53 to 345 mg/kg of dry matter (DM) [9–12], in dairy cattle from 37 to 183 mg/kg of DM [13–16], and in sheep at 50 and 500 mg/animal per day [17]. This compound inactivates methyl-coenzyme M reductase (MCR), the enzyme responsible for the final step of methanogenesis by the rumen methanogens [18] determined the inhibitory concentrations for different methanogens ranged from 0.25 μM for the most sensitive for *Methanobrevibacter* (*Mbb.*) *ruminantium* to > 1 μM for *Methanosphaera stadtmanae* [18]. However, the general inhibition of methanogenesis has raised concerns about hydrogen (H_2_) accumulation because it may impair rumen fiber fermentation [19]. Recent studies have reported that differences in CH_4_ emissions based on diet composition [20], cattle type [21], and low and high emission groups [22] are linked to variations in H_2_ metabolism in the rumen. Further, animal studies have shown that suppression of methanogenesis with the use of 3-NOP in both beef and dairy cattle [23–25] could lead to H_2_ accumulation and shifts the metabolic flow of H_2_ in rumen fermentation from acetate towards propionate or butyrate [9, 10, 15, 16]. Furthermore, assessment of rumen microbiome of early lactation dairy cows under 3-NOP supplementation (60 mg/kg of dry matter) for 15 weeks have revealed that methylotrophic methanogens, a relatively small group of archaea in the rumen, may contribute more significantly to total methanogenesis than previous thought, especially when hydrogenotrophic methanogenesis is inhibited [26]. In addition, some rumen fungi [27] and protozoa [28] produce large amounts of H_2_ via hydrogenosomes, with protozoa defaunation reducing CH_4_ production by up to 11% due to their symbiotic relationship with methanogens [28], while most rumen prokaryotes encode hydrogenases that either produce or utilize H_2_, depending on the species [22]. However, it is not clear whether 3-NOP could also influence the protozoa associated with H_2_ flow. In this context, it is crucial to investigate how 3-NOP affects methanogenesis-related genes and the dynamic flow of molecular H_2_, which is generated during rumen fermentation and consumed by methanogenic and H_2_-utilizing microbes.

We speculated that 3-NOP could affect rumen microbiome differently in beef and dairy cattle as inherent differences in their rumen microbiomes may exist [29, 30] as well as 3-NOP supplementation could lead to shifts in other rumen microbes that related to H_2_ production (bacteria and protozoa) and utilization (bacteria and archaea). Secondly, these microbiome shifts would be dependent on 3-NOP dosage, diet composition, and treatment period. While much of the research on rumen microbes has focused on methanogens, there is lack of understanding of how 3-NOP affects other rumen prokaryotes and eukaryotes, particularly bacteria and protozoa, and their associated functions. Here, we conducted both metataxonomic and metagenomic analyses, combining this data with a comparative analysis of four different studies on 3-NOP that focused on various factors, including cattle breeds (such as Angus and Holstein), CH_4_ emissions, and different 3-NOP dose levels. Specifically, we explored how 3-NOP affected enzymes, including hydrogenase, terminal reductase, and electron transferases, as well as host rumen metabolism, particularly carbohydrate and nitrogen metabolism across microbial (bacterial, archaeal, and protozoa) taxa and investigated whether these changes differed among cattle breeds. We hypothesized that 3-NOP could pose significant impact on other rumen microbial groups such as bacteria and protozoa, and such effects would differ between dairy and beef cattle due to their varied physiology and metabolism. Therefore, our specific objectives were to evaluate both broad and production system-specific effects of 3-NOP on rumen microbiota and their associated functions. The comprehensive understanding of microbial responses to 3-NOP supplementation will enable the development of more effective CH_4_ mitigation strategies in different cattle production systems.

## Methods

### Experiment procedures

The experiments were conducted following the guidelines of the Canadian Council of Animal Care (2011, Ottawa, ON, Canada). The two beef heifer studies were performed at Agriculture and Agri-Food Canada Research Centre in Lethbridge, Alberta, Canada. The two dairy cow studies were performed at Dairy Research and Technology Centre at University of Alberta, Canada.

### Animal experiments, sampling, and diet compositions

A schematic overview of the study design across all four trials (Beef1, Beef2, Dairy1, and Dairy2) is provided in Fig. S1. The sample from beef cattle were from two companion studies conducted previously by Romero-Pérez [9, 10]. The short-term study (Beef1) used 8 ruminally-cannulated Angus heifers in a 4 × 4 Latin square design with four 28-day periods. Dietary treatments included 3-NOP at four levels (control: 0, low: 53, med: 161, and high: 345 mg/kg of DM, respectively). Rumen digesta samples were collected on day-14 of each period at 0 (before feeding), 6, and 12 h after feeding and all samples were used for microbial community analysis, prior to moving the animals to the metabolic chamber for gas and total track digestibility measurements on day-14 and day-28. The long-term study (Beef2) used 8 ruminally-cannulated Angus heifers in a completely randomized design with 2 treatments: (control: 0 and high: 280 mg/kg of DM). The 146-day experiment included an 18-day period without 3-NOP, four 28-day periods with 3-NOP, and a 16-day recovery period without 3-NOP. Methane was measured at the end of each period for 3-days using metabolic chambers. Rumen digesta samples were collected at 0 (before feeding), 3, and 6 h after feeding on day-12 of the covariate period, day-22 of each treatment interval, and day-8 of the recovery period, for total volatile fatty acid (VFA) analysis, with microbial analysis conducted using the samples collected at 3 h post-feeding.

The sample from dairy cattle originated from two companion studies conducted previously by Haisan et al. [15, 16]. The first study (Dairy1) consisted of 12 ruminally-cannulated lactating Holstein cows used in a crossover design study with two periods, using dietary treatments of 3-NOP (control: 0 and high: 130 mg/kg of DM). Each 28-day period had a 21-day adaptation and a 7-day data collection phase. Rumen fermentation variables including pH, ammonia (NH_3_) nitrogen, VFA profiles, and milk composition were assessed. Enteric CH_4_ from individual cows were measured between day-23 and day-27 using the sulfur hexafluoride tracer gas technique [31] as modified by McGinn et al. [32]. Rumen digesta samples were collected on day-22 and day-28 before feeding and all samples were used for microbial community analysis. The second dairy study (Dairy2) used 15 ruminally-cannulated lactating Holstein cows in a 3 × 3 Latin square design with three levels of 3-NOP (control: 0, low: 68, and high: 132 mg/kg of DM). Rumen pH and CH_4_ were recorded as described by Haisan et al. [15]. Rumen digesta samples were collected on day-21 and day-28 before feeding for fermentation measurement and microbial community analyses, respectively.

Detailed feed information for each study is provided in Tables S1–S4. Three studies (Beef1, Beef2, and Dairy2) used similar diets (approximately 60% barley silage, 20–35% barley grain), while Dairy1 used a lower silage (37.9%) and higher grain diet. Additionally, Beef1 study included melengestrol acetate (2.69 mg/day) as a feed additive to suppress estrus, which is a common practice in beef cattle production [33] and has not been reported to affect rumen microbiota or CH_4_ production [34]. These dietary compositions were considered in the comparative analysis to account for potential dietary effects. More details on each study can be found in the respective publications [9, 10, 15, 16].

### Rumen microbial profiling using amplicon sequencing

The rumen digesta samples collected from each study (Beef1: 95 samples; Beef2: 48 samples; Dairy1: 48 samples; Dairy2: 90 samples; total: 281 samples) were processed to extract total microbial DNA using the repeated bead-beating plus column method. Briefly, 0.5 to 1 g of content samples were washed with TE buffer, and subjected to two steps of bead-beating followed by a precipitation. The precipitated DNA was then subjected to purification steps with QIAamp Fast DNA Stool Mini kit following the manufacturer’s protocol. The DNA concentration was measured with NanoDrop and the DNA quality was analyzed on 1% agarose gel. All of the diluted DNA samples (50 ng/μl) were sent to Génome Québec Innovation Centre to amplify bacteria/archaea partial 16S rRNA gene and protozoa partial 18S rRNA gene using primer pairs Bac9F (5′-GAGTTTGATCMTGGCTCAG-3′)/Bac515R (5′-CCGCGGCKGCTGGCAC-3′), Arc915aF (5′-AGGAATTGGCGGGGGAGCAC-3′)/Arc1386R (5′-GCGGTGTGTGCAAGGAGC-3′), and RP841F (5′-GACTAGGGATTGGARTGG-3′)/Reg1302R (5′-AATTGCAAAGATCTATCCC-3′) (obtained from Rumen Consensus Program, Ag/Research, New Zealand [35]), respectively. All of the amplicons were then subjected to pyrosequencing analysis using the 454 Titanium FLX (Roche), and the reads were processed using QIIME2 (v.2024.5) [36]. The DADA2 [37] was used for denoising (qiime dada2 denoise-pyro) and clustering, and the merged representative sequences were aligned to the Silva (v.138.1, [38]) gene database for bacteria and protozoa, and the RIM-DB [39] database for archaea. Relative abundance of taxa in the sample was calculated as the fraction of that taxa observed in the amplicon sequence variant (ASV) table, relative to the sum of all taxa observed for the corresponding sample in the ASV table. Alpha diversity indices (Chao1, evenness, and Shannon index) were calculated using QIIME2 (v.2024.5), and beta diversity was analyzed using the vegan package (v.2.6–6.1) in R Studio (v.4.2.0) with permutational multivariate ANOVA (PERMANOVA).

### Metagenomic sequencing and analysis of rumen microbiome

Based on the CH_4_ emission data, sub samples were chosen based on the top and bottom CH_4_ emissions from each study (Beef1: 16, top 8 and bottom 8; Beef2: 8, top 4 and bottom 4; Dairy1: 12, top 6 and bottom 6; Dairy2: 18, top 9 and bottom 9; total: 54 samples) for metagenomic analysis. Briefly, the metagenome library was prepared using the TruSeq DNA PCR-Free Library Preparation Kit (Illumina, San Diego, CA, USA). Sequencing was performed at the McGill University and Génome Québec Innovation Centre (Montréal, QC, Canada) with the Illumina HiSeq 2500 system, which generated 125 bp paired-end reads. A total of 434.7 Gb of raw reads were generated, with an average of 30.0 million reads per sample. All adapters and low-quality sequences were trimmed using Trimmomatic (v.0.39) [40]. Afterward, reads aligning to the host genome (*Bos taurus*, ARS-UCD2.0/bosTau9), human (*Homo sapiens*), and plant (barley, *Hordeum vulgare subsp. vulgare*) were identified and removed using Bowtie2 (v.2.5.1) [41], and 342.1 Gb (average of 6.34 Gb per sample) of high-quality reads were obtained. These cleaned sequences were used as the input data for the metagenomic assembly. We assembled the sequences from each sample using MEGAHIT (v.1.2.9) [42]. An average of 79.5% of the cleaned reads were used per sample and finally assembled into a total of 20.2 million contigs with a length exceeding 200 bp (Table S5).

### Taxonomy annotation based on metagenomes

Taxonomic profiles were assigned to quality-controlled reads by mapping the sequences to the database of complete genomes for bacteria, archaea, protozoa, and fungi from NCBI using Kraken2 (v.2.1.3) [43]. The database was augmented with Hungate1000 collection [44], 4,941 rumen-related metagenome-assembled genomes (MAGs, [45]), the genome of the protozoan *Entodinium caudatum* [46], 18 single-amplified genomes from Li et al. [47], genomes of *Mbb.* sp. Furthermore, we added available fungal genomes, downloaded from Joint Genome Institute (JGI) *Mycocosm* [48] (*Anaeromyces* sp. S4 [49], *Neocallimastix californiae* G1 [49], *Piromyces finnis* [49], *Piromyces* sp. E2 [49], *Caecomyces churrovis* [50], *Neocallimastix lanati* [51], and *Orpinomyces* sp. [52]). All of the output files were further analyzed using Bracken (v.3.0) [53] to calculate the relative abundance of rumen microbes at the species level.

### Functional annotation based on metagenomes

Prodigal (v.2.6.3) [54] with were used to predict open reading frames (ORFs) with a length of ≥ 100 bp were selected and translated into amino acid sequences. A non-redundant gene catalog was constructed with CD-HIT (v.4.8.1) [55] using the parameters “-c 0.95–aS 0.9”. We finally obtained 3,443,421 nonredundant genes with an average length of 252.7 bp. Functional classification was conducted by querying the predicted protein sequences using DIAMOND (v.2.1.8) [56] based on the BLASTP procedure and specific databases. For hydrogenases and terminal reductases, we used the comprehensive HydDB database established by Greening et al. [22]. Genes encoding subunits of terminal reductases or other metabolic enzymes (including *asrA*, *dmsA*, *dsrA*, *cydA*, *mcrA*, *narG*, *nrfA*, and *nifH*) were identified. Additionally, enzymes involved in reductant disposal via electron transferases, essential for carbohydrate and nitrogen metabolism, include those for propionate production (*LDH*, *MDH*, *ACADS*, *sdhA*, *sucD*, and *frdA*), butyrate production (*fabV* and *paaH*), reductive acetogenesis (*folD*, *fdhA*, and *FHS*), nitrate ammonification (*napA*, *nirB*, and *narB*), glutamate dehydrogenase/synthase (*gdhA*, *gltB*, and *gltD*), and methanogenesis (*MER*, *MTD*, and *mtaB*). These enzymes were annotated using Hidden Markov model (HMM) searches against a local protein database. Genes were subjected to the NCBI-NR database (February 2024; ~ 707 million sequences) for taxonomic and functional assignment using DIAMOND (v.2.1.8) based on BLASTP searches. Identified enzymes were categorized based on their metabolic roles and electron transfer mechanisms following the classification frameworks established in previous studies [20, 21, 57]. All the criteria for gene annotation included an *e* value threshold of 1e-50, identity values exceeding 50%, and amino acid length > 40 residues. KofamScan (v.1.3.0) [58] was used to assign K numbers to the protein sequences using Kyoto Encyclopedia of Genes and Genomes (KEGG) annotation profile database (v.23.12.01). Abundance quantification was conducted using the Salmon (v.1.7.0) [59] and the results were normalized to calculate count per million (CPM). Further, for each study we first split the data by experimental groups (control and 3-NOP treatments), identified features with CPM > 1 in at least 50% of animals within each group, and combined these retained features from all groups for subsequent comparative analysis.

### Comparative analysis of metagenomes from four animal studies

A comparative analysis approach was employed to investigate the impact of 3-NOP across each study, identifying different microbial signatures and functions using MMUPHin (v.1.18.1, Meta-analysis Methods with a Uniform Pipeline for Heterogeneity in Microbiome Studies, [60]). To ensure robust analyses, a comprehensive pre-processing protocol was applied before batch correction, with criteria set to retain only taxa with a relative abundance of ≥ 0.05% for metataxonomic data or ≥ 0.001% for metagenomic data [61], and those present in over 50% of animals in at least one group across four studies. These analyses were performed for both metataxonomic datasets focusing on microbial composition, and metagenomic datasets targeting functional genes related to hydrogenases, reductases, and electron transfer enzymes as well as associated metabolic pathways. This method allowed for batch effect correction between different studies while controlling for 3-NOP dose levels, breed (Angus or Holstein), experimental period, and diet composition (forage and concentrate), which were considered recovery of biological interest for this study. These variables were selectively included to avoid confounding and ensure valid analysis. Briefly, we used the “adjust_batch” function in MMUPHin to reduce batch effects between studies and then applied the “lm_meta” function to combine association effects across batches. The “lm_meta” function performs study-wise association testing using MaAsLin2 (v.1.12.0, Microbiome Multivariable Associations with Linear Models, [62]) internally, followed by aggregation through fixed-effects modeling, enabling robust meta-analytical inference across heterogeneous datasets. In parallel, multivariable analysis was performed using compound Poisson linear model (CPLM) in MaAsLin2 [62] without any normalization or transformation, using the batch-corrected data for overall association testing. Significant differences in taxa or functions were determined based on criteria that satisfied both MMUPHin and MaAsLin2 with *P* < 0.05 and *Q* < 0.05. The coefficients represent the effect size of a variable on the response, while the *Q* value indicates the minimum false discovery rate (FDR) at which a result is considered significant. Additionally, to enhance the robustness of our findings for individual studies, we conducted complementary analyses using ANCOM-BC2 (v.2.0.3, Analysis of Composition of Microbiomes with Bias Correction, [63]) on the same filtered metataxonomic datasets and report the taxa that showed significant changes in both statistical methods (MaAsLin2 and ANCOM-BC2).

### Statistical analysis

Statistical analyses were performed using R software stats (v.4.2.0) and FSA (v.0.9.5). In the Beef2 study, the recovery phase was compared to the 3-NOP supplementation phase using averaged data and analyzed with the Wilcoxon rank-sum test. For individual metagenome datasets and alpha diversity indices, Kruskal–Wallis test with Dunn’s post-hoc test was applied to the Beef1 and Dairy2 studies while Wilcoxon test was used for Beef2 and Dairy1 studies to detect significant differences in taxa and functions across 3-NOP dose levels. All *P* values were adjusted for FDR using the Benjamini-Hochberg (BH) method [64] with an FDR threshold of < 0.05 considered statistically significant.

## Results

To simplify the description of the four studies used in the analysis, the following abbreviations were used: the beef study by Romero-Perez et al. [9] with short-term 3-NOP supplementation was termed Beef1; the beef study by Romero-Perez et al. [10] with long-term 3-NOP supplementation was termed Beef2; the dairy study by Haisan et al. [15] using a high grain diet was termed Dairy1; and the dairy study by Haisan et al. [16] using a high forage diet was termed Dairy2. These four animal studies reported that CH_4_ emissions (g/kg DM intake) were reduced significantly across all four studies: Beef1: − 33%, Beef2: − 59%, Dairy1: − 60%, and Dairy2: − 37% [9, 10, 15, 16]. Additionally, 3-NOP dosages (Beef1: control: 0, low: 53, med: 161, and high: 345 mg/kg of DM; Beef2: control: 0 and high: 280 mg/kg of DM; Dairy1: control: 0 and high: 130 mg/kg of DM; Dairy2: control: 0, low: 68, and high: 132 mg/kg of DM) and the recovery period (recov; only applicable to Beef2) are indicated for each microbial taxon or gene that showed significant differences throughout this section.

### Metataxonomic analysis revealed shifts in bacterial, archaeal, and protozoal communities in response to 3-NOP supplementation of beef and dairy cattle diets

**Fig. 1:**
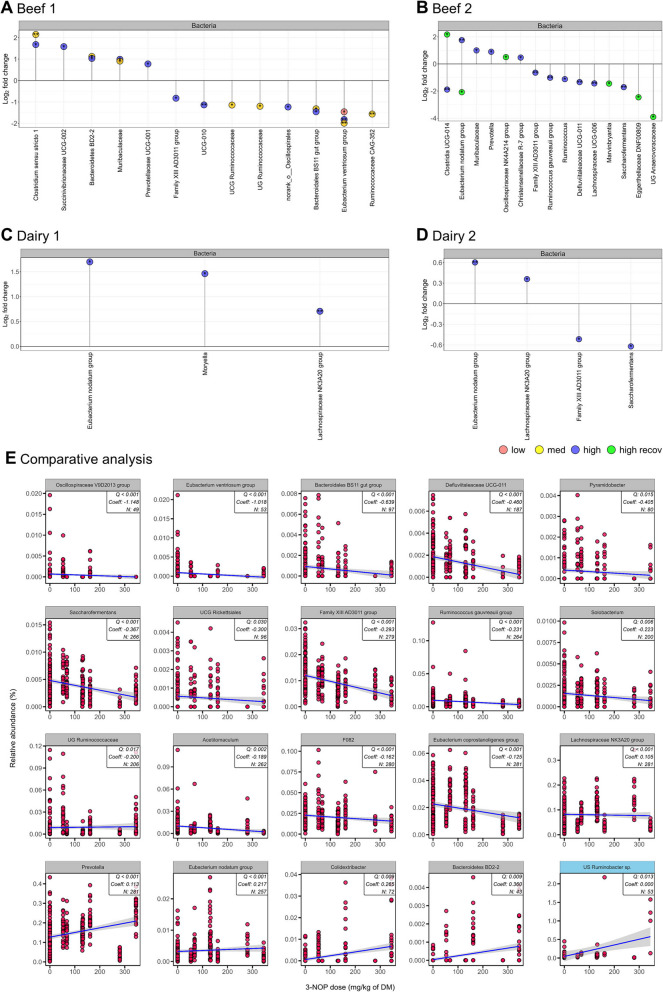
Comparison of rumen bacteria identified with 3-nitrooxypropanol (3-NOP) supplementation of beef and dairy cattle diet. **A**-**D** data were obtained from metataxonomic analysis of individual studies. All microbes were selected based on statistical analysis in individual studies, and log fold change (log 2) was employed for comparison. **E** results were obtained from a comparative analysis using MMUPHin and further validated by MaAsLin2 analysis. The gray and skyblue strip represent metataxonomic and metagenomic data, respectively. Asterisks indicate significance: **FDR < 0.01 and *FDR < 0.05, which were cross-verified by MaAsLin2 and ANCOM-BC2, with significance for Dairy2 observed only in MaAsLin2. Averaged data from the recovery and 3-NOP phases were compared using the Wilcoxon rank-sum test (*P* < 0.05). The comparative analysis was conducted with a threshold of *Q* < 0.05 and cross-verified by MaAsLin2 with *Q* < 0.05. Taxa were included if they were ≥ 0.05% (metataxonomic) or ≥ 0.001% (metagenomic) and appeared in over 50% of animals in at least one group. *recov* recovery period, *UCG* uncultured genus-level, *UCG* unclassified genus-level, *DM* dry matter, *FDR* false discovery rate

**Fig. 2:**
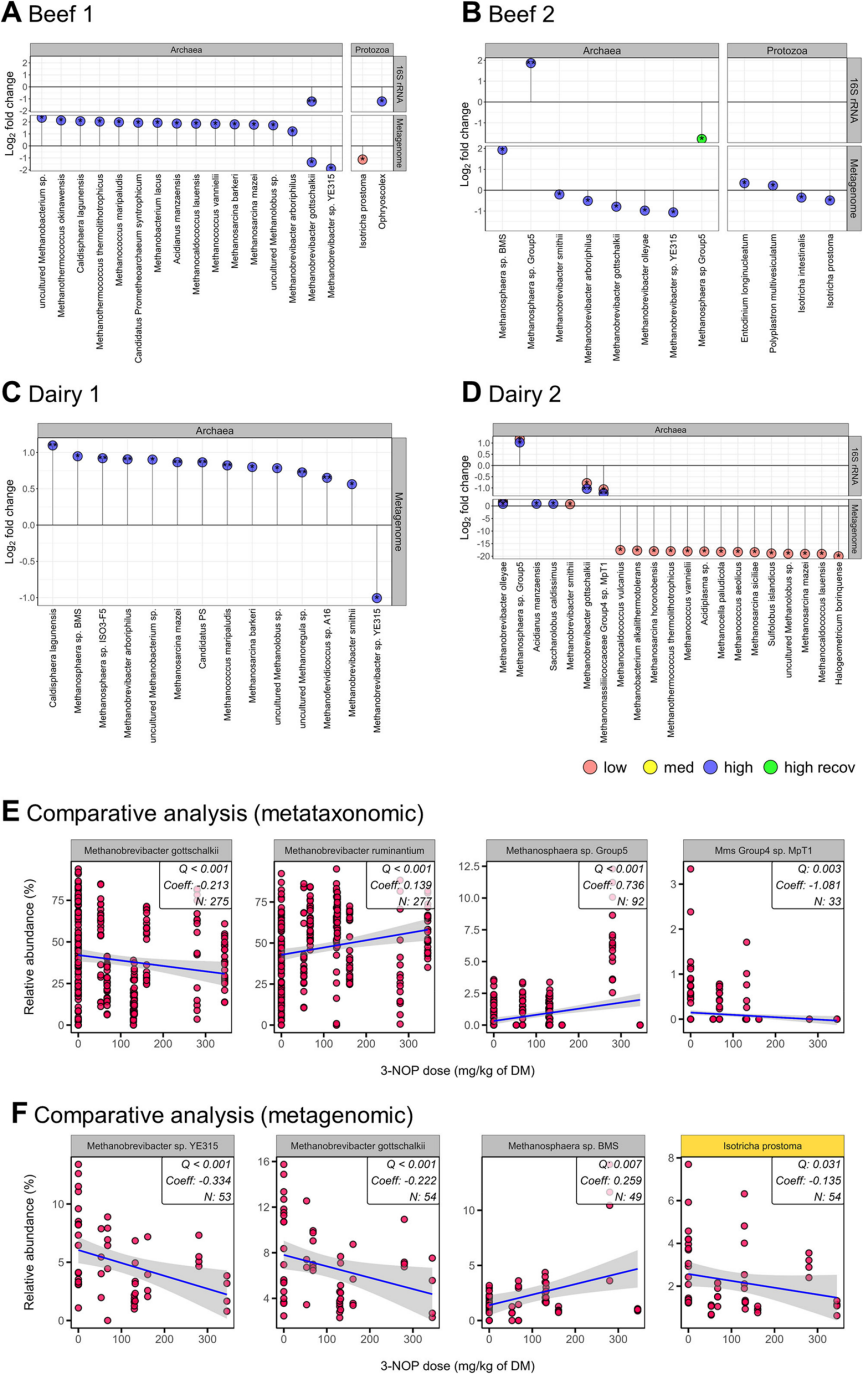
Comparison of rumen microbial taxa (archaea and protozoa) identified with 3-nitrooxypropanol (3-NOP) supplementation of beef and dairy cattle diet. Data were obtained from metataxonomic (16S and 18S rRNA) and metagenome analyses of individual studies. **A-D** results were obtained from individual studies. All microbes were selected based on statistical analysis in individual studies, and log fold change (log 2) was employed for comparison. **E-F** results were obtained from a comparative analysis using MMUPHin and further validated by MaAsLin2 analysis. **E** represents metataxonomic data and **F** represents metagenomic data. The gray and yellow strip represent archaea and protozoa, respectively. Asterisks indicate significance: **FDR < 0.01 and *FDR < 0.05, which were cross-verified by MaAsLin2 and ANCOM-BC2. Averaged data from the recovery and 3-NOP phases were compared using the Wilcoxon rank-sum test (*P* < 0.05). The comparative analysis was conducted with a threshold of *Q* < 0.05 and cross-verified by MaAsLin2 with *Q* < 0.05. Taxa were included if they were ≥ 0.05% (metataxonomic) or ≥ 0.001% (metagenomic) and appeared in over 50% of animals in at least one group. *recov* recovery period, *All* beef and dairy, *Mms Methanomassiliicoccaceae*, *DM* dry matter, *FDR* false discovery rate

A total of 281 rumen samples (Beef1: 95, Beef2: 48, Dairy1: 48, Dairy2: 90) were analyzed for rumen bacterial, archaeal, and protozoal communities after 3-NOP supplementation, revealing differences in the relative abundance of certain bacterial genera across all four studies. The significance of metataxonomic findings (*Q* value) was supported by MaAsLin2. Results from Beef1 showed that 3-NOP supplementation affected 15 bacterial genera, with 5 increased and 10 decreased (Fig. 1A) including *Clostridium *sensu stricto* 1* (Coeff = 0.697, *Q* = 0.039), *Prevotellaceae* UCG-001 (Coeff = 0.317, *Q* < 0.001), and *Succinivibrionaceae* UCG-002 (Coeff = 0.398, *Q* = 0.002), whereas UG *Ruminococcaceae* (Coeff = − 0.267, *Q* = 0.027), UCG *Ruminococcaceae* (Coeff = − 0.320, *Q* = 0.022), *Ruminococcaceae* CAG-352 (Coeff = − 0.244; *Q* = 0.022) and *Eubacterium ventriosum* group (Coeff = − 1.128, *Q* < 0.001) decreased. Beef2 showed that 3-NOP supplementation affected 12 bacterial genera with 5 increased and 7 decreased (Fig. 1B), including *Prevotella* (Coeff = 0.670, *Q* = 0.024), *Eubacterium nodatum* group (Coeff = 1.238; *Q* = 0.001) and *Muribaculaceae* (Coeff = 0.778; *Q* = 0.037) were increased, whereas *Ruminococcus* (Coeff = − 0.829, *Q* = 0.024) and *Ruminococcus gauvreauii* group (Coeff = − 0.968; *Q* = 0.001) decreased. For the recov, in Beef2, 6 bacterial genera were changed, with 2 increased and 4 decreased (Fig. 1B), including *Clostridia* UCG-014 (*P* = 0.029) and *Oscillospiraceae* NK4A214 group (*P* = 0.029) increased, whereas *Eubacterium nodatum* group (*P* = 0.029) and UG *Anaerovoracaceae* (*P* = 0.027) decreased. Results from Dairy1 showed that 3 bacterial genera were increased after 3-NOP supplementation (Fig. 1C), including *Eubacterium nodatum* group (Coeff = 0.478, *Q* = 0.035), *Moryella* (Coeff = 0.398, *Q* = 0.035), and *Lachnospiraceae* NK3A20 group (Coeff = 0.186, *Q* = 0.008). In Dairy2, while MaAsLin2 analysis identified changes in 4 bacterial genera after 3-NOP supplementation (Fig. 1D) with *Lachnospiraceae* NK3A20 group (Coeff = 0.098, *Q* = 0.005) and *Eubacterium nodatum* group (Coeff = 0.196, *Q* = 0.030) increased, whereas *Family XIII* AD3011 group (Coeff = − 0.150, *Q* = 0.013) and *Saccharofermentans* (Coeff = − 0.174, *Q* = 0.041) decreased, ANCOM-BC2 analysis did not detect any significant changes. The relative abundance of archaea also differed after 3-NOP supplementation, with *Mbb*. *gottschalkii* (Coeff = − 0.182, *Q* < 0.001) decreased (Fig. 2A). Beef2 showed *Methanosphaera* sp. Group5 (Coeff = 1.708, *Q* < 0.001) increased and during the recovery period, this species (recov; *P* = 0.029) decreased (Fig. 2B). Dairy1 showed increases in *Methanosphaera sp*. Group5 (Coeff = 1.891, *Q* = 0.002) based on MaAslin2 only (Fig. 2C). Dairy2 showed decreases in *Mbb. gottschalkii* (Coeff = − 0.314, *Q* < 0.001) and *Methanomassiliicoccaceae* Group4 sp. MpT1 (Coeff = − 0.678, *Q* = 0.001), while *Methanosphaera* sp. Group5 (Coeff = 0.610,* Q* = 0.002) increased (Fig. 2D). The protozoa genus *Ophryoscolex* was decreased in the Beef1 study (Coeff = − 0.415, *Q* < 0.001) but this was observed only in MaAslin2 analysis.

Comparative analysis of metataxonomic data revealed that 19 bacterial genera were affected by 3-NOP supplementation, with 14 increased and 5 decreased (Table S6). Similarly, four archaeal species significantly changed, with two increased (*Mbb*. *ruminantium*, *Methanosphaera* sp. Group5) and two decreased (*Mbb*. *gottschalkii*, *Methanomassiliicoccaceae* Group4 sp. MpT1). Comparative analysis using MaAsLin2 via MMUPHin revealed that *Mbb*. *gottschalkii* (Coeff = − 0.002, *Q* = 0.004), *Ruminococcus gauvreauii* group (Coeff = − 0.002, *Q* = 0.002), and UG *Ruminococcaceae* (Coeff = − 0.000, *Q* = 0.003) showed significant decreases. For example, when comparing individual study results between the two statistical methods, the decrease in *Mbb*. *gottschalkii* was detected by MaAsLin2 in three studies (Beef1, Beef2, and Dairy2) and by ANCOM-BC2 in two studies (Beef1 and Dairy2). Additionally, decreased in *Ruminococcus* genera were consistently observed in both Beef1 and Beef2 studies using both methods. While no significant differences were observed for protozoa. Detailed metataxonomic results are provided in supplemental Fig. S2–S5 and Table S7–S11.

We further analyzed the effects of batch correction on rumen microbial diversity. Alpha diversity (Shannon index) patterns remained consistent before and after batch correction (Fig. S6A–C). Beta diversity analysis revealed that batch correction substantially reduced study-specific variations, as evidenced by decreased R^2^ values in PERMANOVA (from bacteria: 46.9% to 14.6%, archaea: 40.5% to 8.2%, and protozoa: 13.7% to 7.5%, respectively; Fig. S6D–F). In addition, alpha diversity (Chao1 and evenness) and beta diversity for individual studies are shown in Fig. S7 and S8, respectively.

### Metagenome of selected samples and bacterial, archaeal, and protozoal communities in response to 3-NOP supplementation of beef and dairy cattle diets

A total of 54 samples (Beef1: 16, Beef2: 8, Dairy1: 12, Dairy2: 18) were selected for metagenomic analysis based on CH_4_ emission data, revealing that 3-NOP had varying impacts on archaeal and protozoal species across the groups. In Beef1, 39 bacterial, 15 archaeal, and 1 protozoal genus were significantly affected, with decreases in *Mbb.* sp. YE315 (Beef1-high; *P* = 0.014), *Mbb*. *gottschalkii* (Beef1-high; *P* = 0.045), *uncultured Ruminobacter* sp. (Beef1-low; *P* = 0.023), and *Isotricha prostoma* (Beef1-low; *P* = 0.035), while *Methanosarcina barkeri* (Beef1-high; *P* = 0.022), *Clostridium* sp. JN-1 (Beef1-low; *P* = 0.028), and uncultured *Clostridium* sp. (Beef1-med; *P* = 0.043) increased (Fig. 2A and Table S12). Beef2 showed significant changes in 41 bacterial, 6 archaeal, and 4 protozoal genera, including decreases in *Mbb. gottschalkii* (Beef2-high; *P* = 0.029), *Mbb*. *smithii* (Beef2-high; *P* = 0.029), *Mbb*. sp. YE315 (Beef2-high; *P* = 0.029), *Clostridium* spp. (Beef2-high; *P* < 0.05), *Isotricha prostoma* (Beef2-high; *P* = 0.029), and *Isotricha intestinalis* (Beef2-high; *P* = 0.029), while *Methanosphaera* sp. BMS (Beef2-high; *P* = 0.029), *Prevotella* sp. E2-28 (Beef2-high; *P* = 0.029), *Entodinium longinucleatum* (Beef2-high; *P* = 0.029), and *Polyplastron multivesiculatum* (Beef2-high; *P* = 0.029) increased (Fig. 2B and Table S13). In Dairy1, 24 bacterial and 14 archaeal genera were significantly impacted, with *Mbb*. sp. YE315 (Dairy1-high; *P* = 0.041) decreasing, while *uncultured Clostridium* sp. (Dairy1-high; *P* = 0.037), *uncultured Succiniclasticum* sp. (Dairy1-high; *P* = 0.037), *Mbb*. *smithii* (Dairy1-high; *P* = 0.026), and *Methanosphaera* sp. ISO3-F5 (Dairy1-high; *P* = 0.009) increased (Fig. 2C and Table S14). Dairy2 showed significant differences in 460 bacterial and 18 archaeal genera, including increases in *Mbb*. *olleyae* (Dairy2-low; *P* = 0.002, Dairy2-high; *P* = 0.040), *Mbb*. *smithii* (Dairy2-low; *P* = 0.028), *Clostridium kluyveri* (Dairy2-low; *P* = 0.032), and *uncultured Sarcina* sp. (Dairy2-low; *P* = 0.007), while *uncultured Selenomonadaceae bacterium* (Dairy2-low; *P* = 0.001) decreased (Fig. 2D and Table S15). Comparative analysis of the metagenomic data revealed effect on several microbial species (Fig. 2F and Table S16), with increases observed in *uncultured Ruminobacter* sp. (Coeff = 0.0003, *Q* = 0.013, Fig. 1E) and *Methanosphaera* sp. BMS (Coeff = 0.259, *Q* = 0.007), while decreases were found in *Mbb*. *gottschalkii* (Coeff = − 0.222, *Q* < 0.001) and *Isotricha prostoma* (Coeff = − 0.135, *Q* = 0.031).

### Effect of 3-NOP on distributions of hydrogenases and associated terminal reductases in the rumen of beef and dairy cattle

**Fig. 3:**
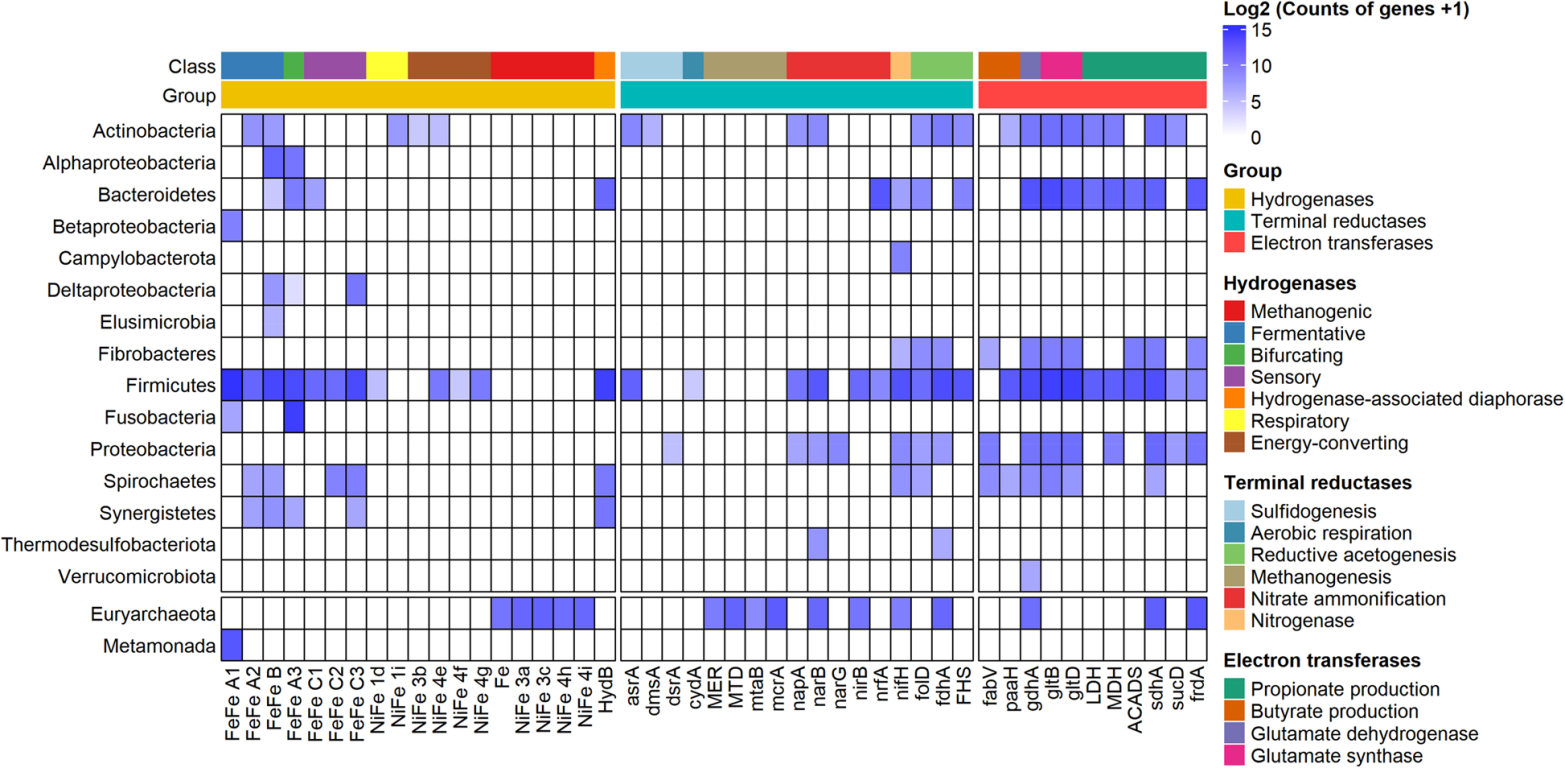
Effect of 3-nitrooxypropanol (3-NOP) supplementation of diets on the distributions of hydrogenases, associated terminal reductases, and electron transferases in beef and dairy cattle. Distributions (in phyla with hydrogenase-encoding genes) of fermentative hydrogenases (group A1, A2, and B FeFe-hydrogenases), bifurcating hydrogenases (group A3 FeFe-hydrogenases), sensory hydrogenases (group C FeFe-hydrogenases), respiratory hydrogenases (group 1 d and 1i NiFe-hydrogenases), energy-converting hydrogenases (bidirectional; group 3b, 4e, 4f, and 4 g NiFe-hydrogenases), and methanogenic hydrogenases (Fe-hydrogenases, group 3a, 3c, 4 h, and 4i NiFe-hydrogenases). *HydB* hydrogenase-associated diaphorase. The H_2_ uptake pathway includes genes involved in sulfidogenesis (*asrA*, alternative sulfite reductase; *dmsA*, DMSO and TMAO reductase, *dsrA*, dissimilatory sulfite reductase), aerobic respiration (*cydA*, cytochrome bd oxidase), methanogenesis (*MER*, 5,10–5,10-methylenetetrahydromethanopterin reductase; *MTD*, methylenetetrahydromethanopterin dehydrogenase; *mtaB*, methanol-5-hydroxybenzimidazolylcobamide Co-methyltransferase; *mcrA*, methyl-CoM reductase), nitrate ammonification (*napA*, periplasmic nitrate reductase; *narB*, ferredoxin nitrate reductase; *narG*, dissimilatory nitrate reductase; *nirB*, nitrite reductase; *nrfA*, ammonia-forming nitrite reductase, and *nifH*, nitrogenase, reductive acetogenesis) (*folD*, methylenetetrahydrofolate dehydrogenase; *fdhA*, formate dehydrogenase; *FHS*, formate-tetrahydrofolate ligase). Electron transfer-related genes involved in butyrate production (*fabV*, crotonoyl-CoA reductase; *paaH*, acetoacetyl-CoA reductase), glutamate dehydrogenase/synthesis (*gdhA*, glutamate dehydrogenase; *gltB*, glutamate synthase large chain; *gltD*, glutamate synthase small chain), propionate production (*LDH*, lactate dehydrogenase; *MDH*, malate dehydrogenase; *ACADS*, acyl-CoA dehydrogenase; *sucD*, succinyl-CoA synthetase; *sdhA*, succinate dehydrogenase; *frdA,* fumarate reductase). Only genes that were detected in at least 50% of all 54 samples and met the criterion of having a count per million (CPM) > 1 are shown

The normalized abundance of genes (CPM) associated with hydrogenases, terminal reductases, and electron transferases, categorized by phylum and functional groups were presented in Fig. 3. For the hydrogenases, seven classes were detected including fermentative (FeFe A1, A2, and B), bifurcating (FeFe A3), sensory (FeFe C1, C2, and C3), respiratory (NiFe 1 d and 1i), energy-converting (NiFe 3b, 4e, 4f, and 4 g), methanogenic (Fe, NiFe 3a, 3c, 4 h, and 4i), and hydrogenase-associated diaphorase (*HydB*). For the terminal reductases, we detected six classes were detected including sulfidogenesis (*asra*, alternative sulfite reductase; *dmsA*, DMSO and TMAO reductase; and *dsrA*, dissimilatory sulfite reductase), aerobic respiration (*cydA*, cytochrome bd oxidase), methanogenesis (*MER*, 5,10-methylenetetrahydromethanopterin reductase; *MTD*, methylenetetrahydromethanopterin dehydrogenase; *mtaB*, methanol-5-hydroxybenzimidazolylcobamide Co-methyltransferase; and *mcrA*), nitrate ammonification (*napA*, nitrate reductase (cytochrome); *narl*, nitrate reductase gamma subunit; *narB*, ferredoxin-nitrate reductase; *narG*, nitrate reductase; *nirB*, nitrate reductase; and *nrfA*, nitrate reductase), nitrogenase (*nifH*), and reductive acetogenesis (*folD*, methylenetetrahydrofolate dehydrogenase; *fdhA*, formate dehydrogenase alpha subunit; and *FHS*, formate tetrahydrofolate ligase). For the electron transferases, we detected 3 classes: glutamate dehydrogenase/synthase (*gdhA*, glutamate dehydrogenase; *gltB*, glutamate synthase large chain; and *gltD*, glutamate synthase small chain), propionate production (*LDH*, lactate dehydrogenase; *MDH*, malate dehydrogenase; *ACADS*, acyl-CoA dehydrogenase; *sdhA*, succinate dehydrogenase; *sucD*, succinyl-CoA synthetase; and *frdA*, fumarate reductase), and butyrate production (*fabV*, Crotonoyl-CoA reductase; *paaH*, Acetoacetyl-CoA reductase; and *ACADS*). Detailed information on hydrogenases and terminal reductases from the individual studies is provided in Fig. S9–12 and Table S17.

### Effect of 3-NOP on volatile fatty acid biosynthesis in beef and dairy cattle

**Fig. 4:**
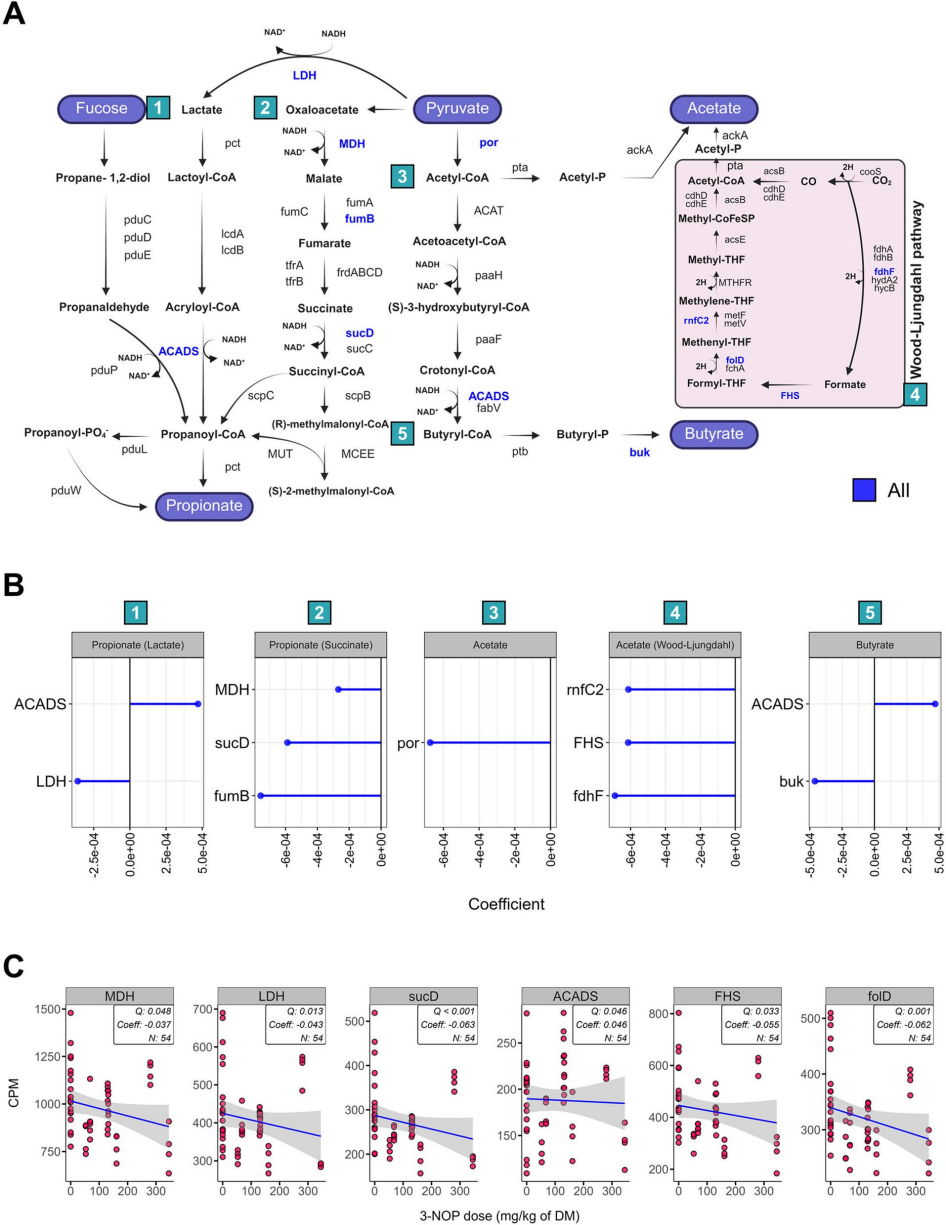
Results of a comparative analysis on the effect of 3-nitrooxypropanol (3-NOP) supplementation on acetate, propionate, and butyrate production pathways in beef and dairy cattle. **A**, **B** indicate the effects of 3-NOP on pathways for acetate, propionate, and butyrate: 1 and 2 are for propionate, 3 and 4 are for acetate, and 5 is for butyrate. **C** indicate the gene’s enrichment involved in the acetate, propionate, and butyrate metabolism. Each color indicates the affected gene related to the groups: all (blue). The comparative analysis was conducted with a threshold of *Q* < 0.05 and cross-verified by MaAsLin2 with *Q* < 0.05. Only genes that were detected in at least 50% of all 54 samples and met the criterion of having a count per million (CPM) > 1 are shown. *All* beef and dairy, *DM* dry matter

Previous animal studies consistently demonstrated an increased molar proportion of propionate across all four studies and an increased molar proportion of butyrate in most studies [9, 10, 15], except for the Dairy1 [16], while the molar proportion of acetate was decreased in all studies. Therefore, the impact of 3-NOP supplementation on VFA production pathways in the rumen microbiome was investigated, and revealed two pathways each for propionate and acetate production, and one for butyrate production (Fig. 4A). Comparative analysis revealed significant changes in several key genes (Fig. 4B, C), with the lactate-utilizing propionate pathway showing a decrease in *LDH* abundance (Coeff = − 0.043, *Q* = 0.013) and an increase in *ACADS* (Coeff = 0.046, *Q* = 0.046), while the succinate-utilizing propionate pathway showed decreases in all components: *MDH* (Coeff = − 0.037, *Q* = 0.048), *fumB* (fumarate hydratase) (Coeff = − 0.001, *Q* = 0.041), and *sucD* (Coeff = − 0.063, *Q* < 0.001). Acetate production was affected across multiple genes, all showing decreases: por (pyruvate-ferredoxin; Coeff = − 0.001, *Q* = 0.008), *fdhF* (formate dehydrogenase; Coeff = − 0.001, *Q* = 0.039), *FHS* (Coeff = − 0.055, *Q* = 0.033), *folD* (Coeff = − 0.062, *Q* = 0.001), and *rnfC2* (ion-translocating oxidoreductase; Coeff = − 0.001, *Q* = 0.007). In the butyrate production pathway, we observed an increase in *ACADS* (Coeff = 0.046, *Q* = 0.046) and a decrease in *buk* (butyrate kinase; Coeff = − 0.000, *Q* = 0.034). Detailed information is provided in the additional file (Table S18).

### Effect of 3-NOP on microbial functions including methanogenesis: mcrA, hydrogenases, terminal reductases, and electron transferases across all four studies

**Fig. 5:**
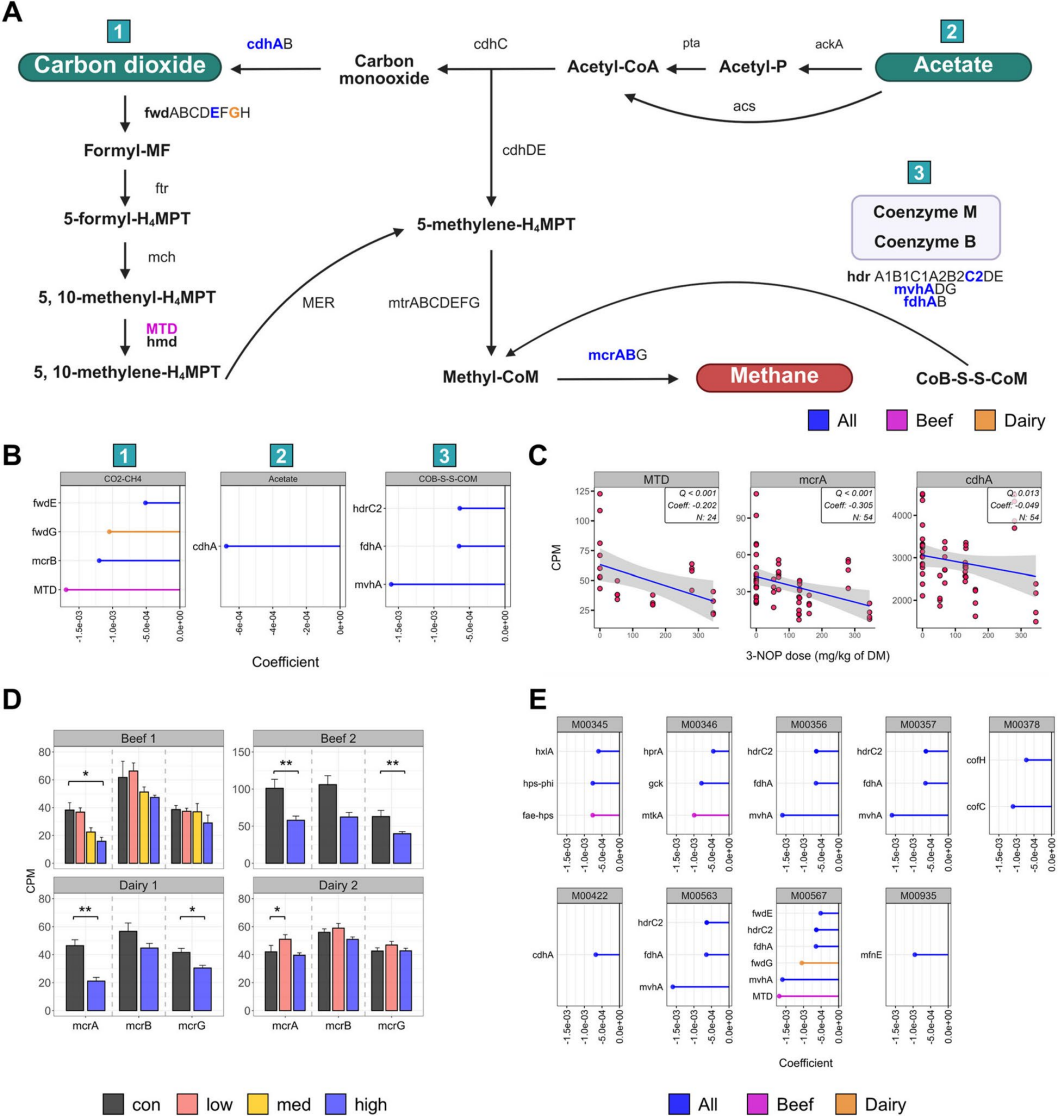
Results of a comparative analysis on the effect of 3-nitrooxypropanol (3-NOP) supplementation on methanogenesis in beef and dairy cattle. **A**, **B** indicate the effects of 3-NOP on methanogenesis: 1 is for CO_2_ to CH_4_, 2 is for acetate to CH_4_, and 3 is for heterodisulfide between coenzyme B and coenzyme M. **C** indicate the gene’s enrichment involved in the methanogenesis. **D** indicates methyl-CoM reductase for individual studies. **E** indicates module of methane metabolism. Each color indicates the affected gene related to the groups: all (blue), beef (magenta), and dairy (orange). M00345, formaldehyde assimilation ribulose monophosphate pathway; M00346, formaldehyde assimilation serine pathway; M00356, methanol → CH_4_; M00357, acetate → CH_4_; M00378, F420 biosynthesis; M00422, CO_2_ → acetyl-CoA; M00563, methylamine/dimethylamine/trimethylamine → CH_4_; M00567, CO_2_ → CH_4_; M00935, Methanofuran biosynthesis. Asterisks indicate significance: **P.adj < 0.01, *P.adj < 0.05 (beef 1 and dairy 2) and **P < 0.01, *P < 0.05 (beef 2 and dairy 1). The comparative analysis was conducted with a threshold of *Q* < 0.05 and cross-verified by MaAsLin2 with *Q* < 0.05. Only genes that were detected in at least 50% of all 54 samples and met the criterion of having a count per million (CPM) > 1 are shown. *All* beef and dairy, *DM* dry matter, *CO*_2_ carbon dioxide, *CH*_4_ methane

Methanogenesis-related gene abundance was significantly altered by 3-NOP supplementation, primarily affecting pathways converting carbon dioxide (CO_2_) and acetate to CH_4_, while the methylated-amine and methanol pathways showed no significant changes (Fig. 5A). Comparative analysis revealed decreased abundance across all affected methanogenesis genes, with some changes specific to beef or dairy cattle, respectively (Fig. 5B and C). In the CO_2_ to CH_4_ pathway, *fwdE* (formylmethanofuran dehydrogenase subunit E; Coeff = − 0.001, *Q* = 0.049), *fwdG* (4Fe-4S ferredoxin, dairy-specific; Coeff = − 0.001, *Q* = 0.039), and *MTD* (beef-specific; Coeff = − 0.202, *Q* < 0.001) decreased. The acetate to CH_4_ pathway showed decreased *cdhA* (anaerobic carbon-monoxide dehydrogenase; Coeff = − 0.049, *Q* = 0.013). Coenzyme B-Coenzyme M heterodisulfide (COB-S–S-COM)-related genes also decreased: *hdrC2* (heterodisulfide reductase subunit C2; Coeff = − 0.001, *Q* = 0.049), *FdhA* (Coeff = −0.001, *Q* = 0.045), and *mvhA* (F420-non-reducing hydrogenase; Coeff = − 0.002, *Q* = 0.003). *mcrA* (Coeff = − 0.305, *Q* < 0.001) and *mcrB* (Coeff = − 0.001, *Q* = 0.031) decreased, with individual studies confirming effects on the *mcrABG* complex (Fig. 5D). Further investigation of CH_4_ metabolism modules revealed that the abundance of these genes significantly decreased after 3-NOP supplementation (Fig. 5E).

**Fig. 6:**
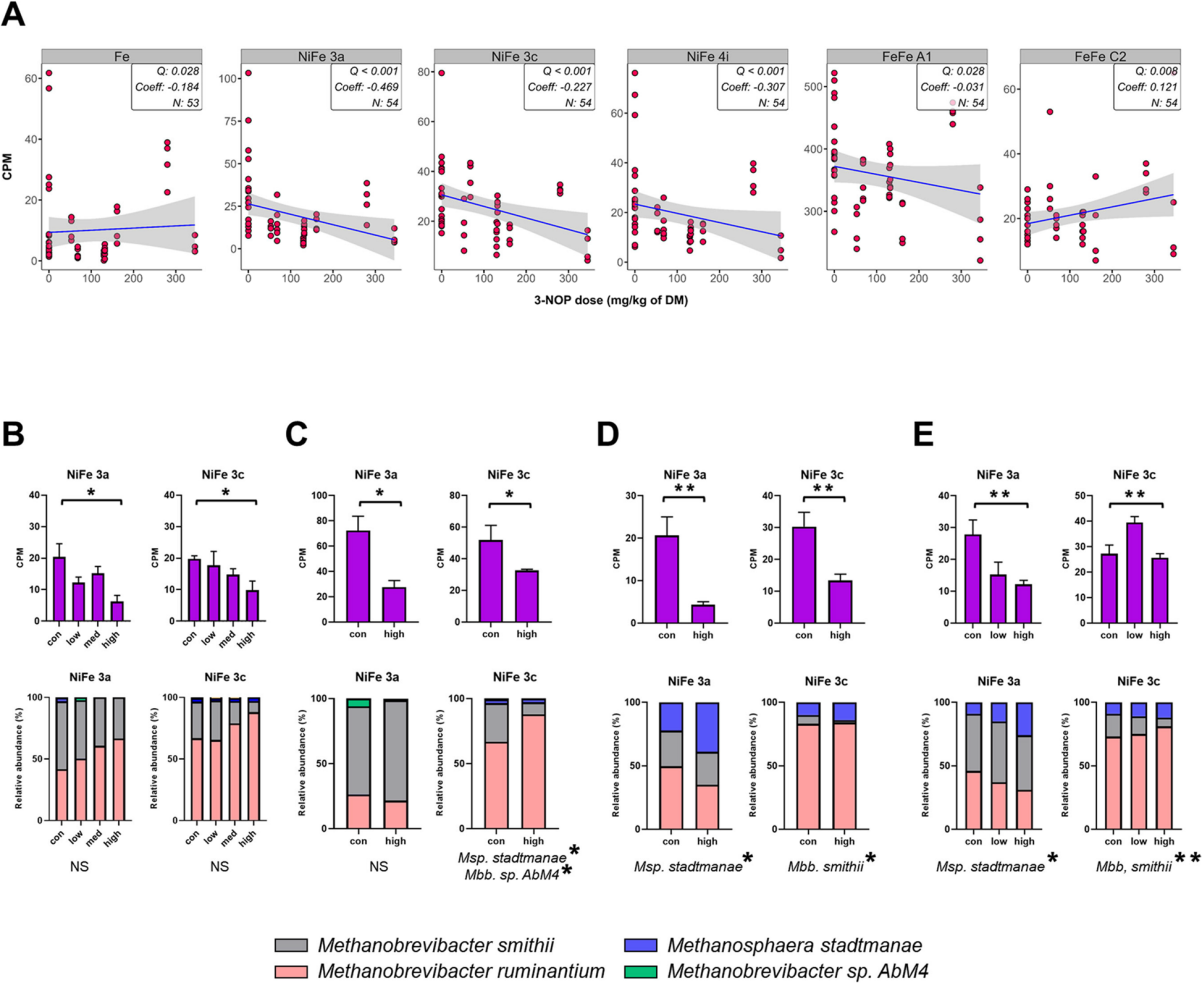
Effects of 3-nitrooxypropanol (3-NOP) supplementation on hydrogenase in beef and dairy cattle. **A** results were obtained from a comparative analysis using MMUPHin and further validated by MaAsLin2 analysis. **B-E** results were from individual studies, which all impacted methanogenic hydrogenases (NiFe 3a and NiFe 3c) and gene driver microbes. Asterisks indicate significance: **FDR < 0.05, *FDR < 0.1 (beef 1 and dairy 2) and **P < 0.05, *P < 0.1 (beef 2 and dairy 1). The comparative analysis was conducted with a threshold of *Q* < 0.05 and cross-verified by MaAsLin2 with *Q* < 0.05. Only genes that were detected in at least 50% of all 54 samples and met the criterion of having a count per million (CPM) > 1 are shown. *Msp Methanosphaera*, *Mbb Methanobrevibacter*, *DM* dry matter, *FDR* false discovery rate

Regarding hydrogenase, the results showed that methanogenic hydrogenases, including Fe (Coeff = − 0.184, *Q* = 0.028), NiFe 3a (Coeff = − 0.469, *Q* < 0.001), NiFe 3c (Coeff = − 0.227, *Q* < 0.001), and NiFe 4i (Coeff = − 0.307, *Q* < 0.001) as well as the fermentative hydrogenase FeFe A1 (Coeff = − 0.031, *Q* = 0.028) were decreased, whereas the sensory hydrogenase FeFe C2 (Coeff = 0.121, *Q* = 0.008) was increased after 3-NOP supplementation (Fig. 6A). Furthermore, we found NiFe 3a and NiFe 3c hydrogenases consistently decreased across all four studies after 3-NOP supplementation (Fig. 6B–E). We investigated the driving microbes responsible for these hydrogenases. For NiFe 3a, we found that *Methanosphaera stadtmanae* was the primary driving microbe, and interestingly, its abundance increased with 3-NOP supplementation. In contrast, NiFe 3c was associated with multiple driving microbes. *Mbb*. sp. *AbM4* and *Mbb*. *smithii*, both responsible for NiFe 3c, decreased with 3-NOP supplementation of diets. However, *Methanosphaera stadtmanae*, also associated with NiFe 3c, exhibited increased abundance after 3-NOP supplementation.

**Fig. 7:**
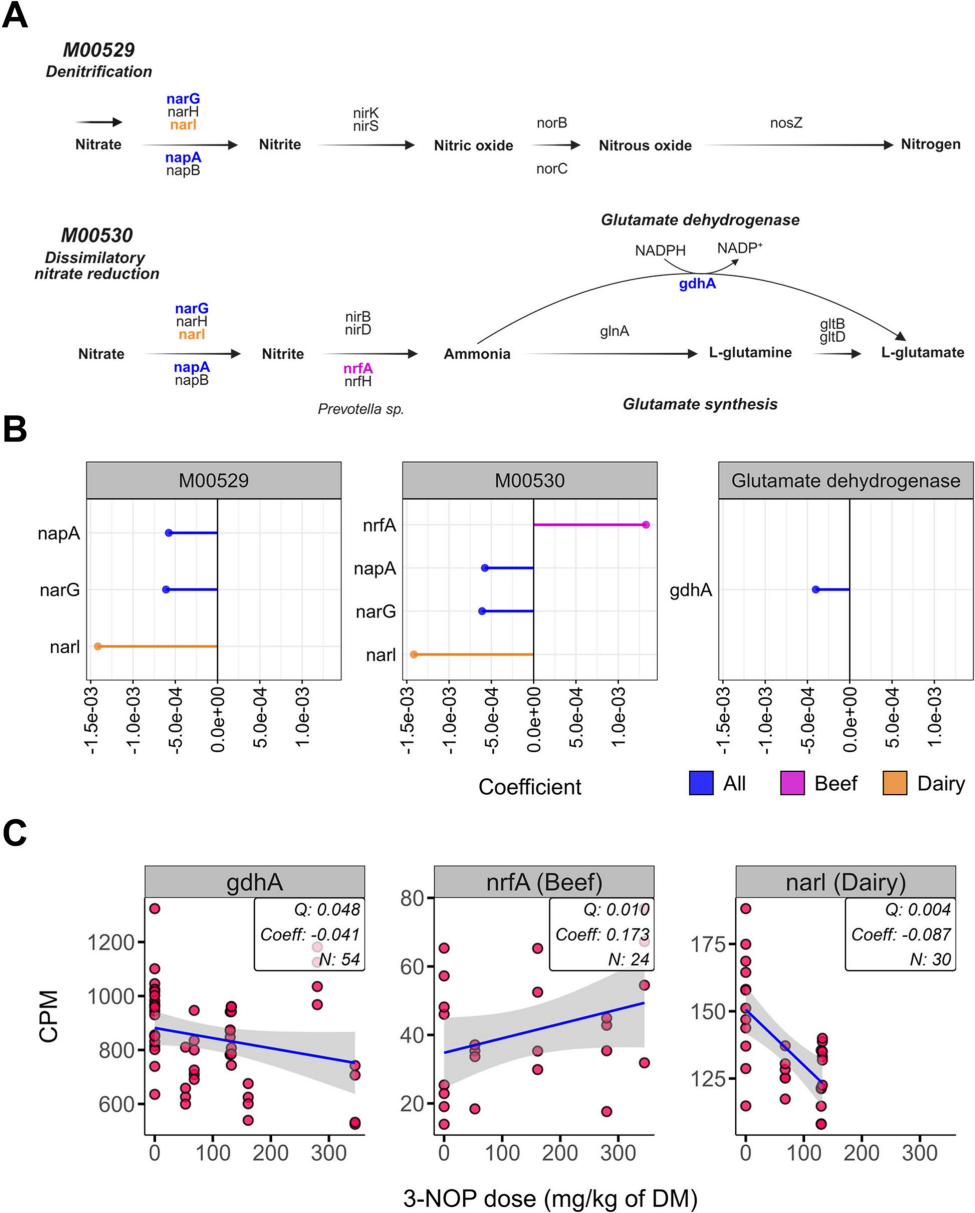
Results of a comparative analysis on the effect of 3-nitrooxypropanol (3-NOP) supplementation on nitrogen metabolism in beef and dairy cattle. **A**, **B** indicate the effects on pathways: 1 is for nitrate to nitrite, 2 is for nitrate to ammonia, and 3 is for glutamate synthesis. **C** indicates the gene’s enrichment involved in the nitrogen metabolism and glutamate synthesis. *nrfA* was only shown in beef heifers, whereas *narl* was only shown in dairy cows. Each color indicates the affected gene related to the groups: all (blue), beef (magenta), and dairy (orange). M00529, Denitrification, nitrate → nitrogen; M00530, Dissimilatory nitrate reduction, nitrate → ammonia. The comparative analysis was conducted with a threshold of *Q* < 0.05 and cross-verified by MaAsLin2 with *Q* < 0.05. Only genes that were detected in at least 50% of all 54 samples and met the criterion of having a count per million (CPM) > 1 are shown. *All* beef and dairy, *DM* dry matter

Nitrogen metabolism and glutamate dehydrogenase/synthase pathways, which serve as important H_2_ and electron sinks [65], were investigated (Fig. 7A). Most nitrate and nitrite reductase genes decreased, with some changes specific to either beef or dairy cattle (Fig. 7B and C). In both denitrification (M00529) and dissimilatory nitrate reduction (M00530) pathways, *napA* (Coeff = − 0.001, *Q* = 0.037), *narG* (Coeff = − 0.001, *Q* = 0.009), and *narl* (dairy-specific; Coeff = − 0.087, *Q* = 0.004) decreased after 3-NOP supplementation. Conversely, *nrfA* (beef-specific; Coeff = 0.173, *Q* = 0.010) increased, but only in the dissimilatory nitrate reduction pathway. Additionally, in the glutamate dehydrogenase pathway, *gdhA* decreased (Coeff = − 0.041, *Q* = 0.048). Detailed information including statistical coefficients, *Q* values, and module information for all significantly affected genes in CH_4_, volatile fatty acid, and nitrogen metabolism pathways is provided in the additional file (Table S19).

## Discussion

The current comparative analysis revealed the comprehensive effects of supplementation of 3-NOP at different dosages and periods that could alter microbial dynamics and the key enzymes including hydrogenase, terminal reductase, and electron transferases involved in H_2_ metabolism in the rumen of both beef and dairy cattle. Although previous meta-analyses have examined the effects of 3-NOP on CH_4_ emissions in ruminants [66–68], our study uniquely focuses on its impact on rumen microbiome based on metagenome datasets generated from four animal trials conducted in Canada. The present study used samples generated from four earlier investigations of 3-NOP in beef and dairy cattle with dose levels (beef: 53, 161, 280, and 345 mg/kg of DM and dairy: 68, 130, and 132 mg/kg of DM) that were above current commercial recommendations [69], yet these data remain valuable for understanding dose-dependent microbial responses in the rumen.

As expected, MCR gene abundance was decreased across all four studies (Table S20) with a consistent trend of decreased *Mbb*. *gottschalkii* and increased *Mbb*. *ruminantium* and *Methanosphaera* sp. with 3-NOP supplementation, which was unexpected. This differential response in various methanogenic species represents a novel in vivo observation and provides new insights into methanogenic species-specific responses to the supplementation of 3-NOP. Our results are consistent with previous studies using 3-NOP in dairy cattle [23, 26] and align with Duin et al.’s [18] finding in vitro that *Mbb.* sp. (< 1 µM) is highly sensitive to 3-NOP, whereas *Methanosphaera sp*. (> 1 µM) shows higher tolerance. Additionally, a previous study showed that *Methanosphaera* sp. was more common in low CH_4_-emitting sheep, while *Mbb*. *gottschalkii* was more abundant in high CH_4_-emitting sheep [70], indicating that not all methanogens respond to 3-NOP in the same manner and are associated with CH_4_ reduction. The observed methanogenic species-specific responses can be hypothesized to stem from the varying distribution of MCR isoforms among these species [71]. Methyl coenzyme M reductase is encoded by two isomeric genes: *mcrI* (isoform I) typically expressed under low H_2_ conditions, and *mrt* (isoform II) expressed under high H_2_ conditions [72]. These isoforms differ in catalytic properties and expression conditions [73–75], with *mcrI* showing superior substrate specificity (lower Km) and *mrt* having a higher maximum turnover rate (Vmax) [73]. The structural variations between isoforms may result in different responses to 3-NOP among methanogenic species, as 3-NOP competes with methyl-CoM for MCR binding. *Mbb*. *ruminantium* encodes *mcrI* only [76], while *Methanosphaera* sp. exclusively contains *mrt* [71, 77]. The presence of which isoforms in *Mbb*. *gottschalkii* remains uncertain due to the lack of a complete genome sequence. These differences suggest varying H_2_ utilization efficiency in different methanogen species, which may influence their response to 3-NOP. *Methanosphaera* sp. appeared less affected by 3-NOP treatment, which could potentially be associated with the presence of *mrt*, though this relationship requires further investigation. While the H_2_ accumulation caused by 3-NOP creates conditions that favor *mrt* expression compared to *mcrI*, the unexpected increase in *Mbb*. *ruminantium* might be associated with its ability to utilize formate, which is discussed in detail later. Notably, similar to the observation of the total methanogen abundance in our previous Beef2 study [10], we confirmed that *Mbb*. *gottschalkii* and *Mbb*. *ruminantium* showed no significant changes, while *Methanosphaera* sp. decreased when 3-NOP supplementation was discontinued, suggesting varied responses among different methanogen species. Although *Methanosphaera sp*. increased under 3-NOP supplementation, our comparative analysis showed no significant differences in genes (*mtaA*, *mtaC*, and *mtbA*) related to methylotrophic methanogenesis. This suggests that the enrichment of methylotrophic methanogens may not result from enhanced activity of this pathway, but potentially reflects a limited functional compensation for suppressed hydrogenotrophic methanogenesis. Further, metatranscriptomic and metaproteomic analyses are needed to clarify the functional activity of this pathway and evaluate the long-term response of methanogenic communities to 3-NOP-induced selective pressure.

Ruminal methanogenesis mainly occurs through three pathways: hydrogenotrophic, methylotrophic, and acetoclastic [78]. Hydrogenotrophic methanogens (*Mbb.* sp.) primarily use H_2_/CO_2_ as substrates for CH_4_ production [79], methylotrophic methanogens (*Methanosarcina*, *Methanosphaera*, and *Methanococcus*) mainly utilize methylated-amines and methanol [80], while acetoclastic methanogens (*Methanosarcinales*) use acetate for CH_4_ production [81]. We found that 3-NOP was associated with a decreased abundance of genes involved in hydrogenotrophic methanogenesis, contributing to a decrease in CH_4_ production in ruminants across all four studies.

Of the 13 CH_4_ metabolism modules, genes involved in 9 modules showed significant decreases after 3-NOP supplementation, with notable decrease in genes of the hydrogenotrophic methanogenesis pathway, highlighting its comprehensive impact on methanogenesis pathways. This contrasts with a previous study that reported no difference in this pathway [26], possibly due to the lower 3-NOP dose used (60 mg/kg of DM) compared to our wider range (beef: 53, 161, 280, and 345 mg/kg of DM and dairy: 68, 130, and 132 mg/kg of DM). The decreased abundance of *fwdE* and *fwdG* (only in dairy) by 3-NOP may suggest an impact on the initial stage of this process, as these enzymes are critical components of the formyl-MF dehydrogenase (*fwd*) complex, which catalyzes the first step of this pathway by reducing CO_2_ to formyl-MF [81]. The genes *fwdE* and *fwdG* are part of the electron-supplying core and catalytic units [81], and these genes are known to be encoded by several methanogens including *Mbb*. *gottschalkii*, *Mbb*. *ruminantium*, *Mbb*. *smithii*, and *Methanosphaera* sp. in NCBI database. The main difference between beef and dairy cattle was the higher abundance of *Mbb*. *smithii* in dairy cattle, suggesting that the decrease of *fwdG* might be related to *Mbb*. *smithii* abundance. However, from our metagenome data revealed that *fwdG* was primarily encoded by unknown microorganisms rather than *Mbb*. *smithii*. This suggests that our understanding of *fwdG* distribution in methanogens might be incomplete, as these genes could be more widely distributed across different microbial groups in the rumen environment. Further, research is needed to improve our understanding of the taxonomic distribution of these key methanogenic genes in the rumen. Particularly notable effect of 3-NOP supplementation was the decrease of *MTD* in beef cattle, a gene is crucial for converting 5,10-methenyl-H_4_MPT to 5,10-methylene-H_4_MPT, using F_420_H_2_ as an electron donor [81]. This *MTD* decrease, primarily encoded by *Mbb*. sp., corresponding with a significant decrease in *Mbb*. sp. *Abm4* and *Mbb*. *smithii*, which drive NiFe 3a and 3c hydrogenase activities. While NiFe 3a and 3c were decreased in both beef and dairy cattle, the specific decrease of *MTD* in beef cattle suggests a more significant disruption of the methanogenesis pathway in this group. This effect was more pronounced in beef cattle studies, while in dairy cattle, only the Dairy1 study (high-grain diet) showed a decrease, suggesting diet composition plays a crucial role. These findings are consistent with Greening et al. [22] who reported that the F_420_-reducing NiFe 3a hydrogenase and the heterodisulfide reductase-associated NiFe 3c hydrogenase of *Mbb*. sp. was among the most transcribed of all H_2_ uptake enzymes in the CO_2_-reducing pathway of methanogenesis. Furthermore, *mvhA*, a key part of NiFe hydrogenase crucial for H_2_ oxidation and electron transfer in hydrogenotrophic methanogenesis [82], was decreased. This likely affected the overall activity of NiFe hydrogenase, impairing H_2_ utilization and electron transfer in methanogenesis [82]. Based on these findings, 3-NOP could work at various aspects to decrease hydrogenotrophic methanogenesis, affecting the initial CO_2_ conversion, intermediate electron transfer steps, and the final CH_4_-forming reaction based on NiFe hydrogenase and *Mbb*. sp. While specific secondary effects of 3-NOP have not been extensively reported, the higher 3-NOP dose levels in the present studies potentially contribute to greater decrease in gene abundances compared to previous results [26].

Comparative analysis based on metataxonomic datasets revealed an increase in *Prevotella*, a genus known for propionate production [83], along with the *Eubacterium nodatum* group [84] and *Lachnospiraceae* NK3A20 group [85], which are involved in butyrate production after 3-NOP supplementation. These microbial changes align with increased propionate production across all four studies and increased butyrate in most studies [9, 10, 15], except for the Dairy1 [16]. In Beef2, which included a recovery period, CH_4_ emissions, propionate, and butyrate returned to normal levels when 3-NOP supplementation was discontinued [10]. These results indicate that increased propionate and butyrate synthesis, facilitated by these microbial changes, is a key mechanism contributing to decreased CH_4_ emissions with 3-NOP supplementation. This is supported by the increased molar proportion of propionate and butyrate observed in all studies. The most sensitive bacterial genus was the *Eubacterium nodatum* group, which increased after 3-NOP supplementation and decreased when 3-NOP was discontinued. This response pattern suggests its crucial role in 3-NOP-induced alterations of rumen metabolism and CH_4_ reduction. This genus can produce formate, acetate, and butyrate as end products [84], potentially contributing to alternative H_2_ utilization pathways, though its specific functional role needs further investigation. While some *Eubacterium* species such as *Eubacterium limosum* are known homoacetogens that utilize H_2_ or other electron donors with CO_2_ as a terminal electron acceptor [86], and while they typically do not compete with methanogens for H_2_ in the rumen [87], they become important when methanogens are decreased [88, 89].

When comparing beef and dairy cattle responses, we found distinct responses after 3-NOP supplementation. In beef cattle, 21 bacterial genera were affected, whereas 6 were affected in dairy cattle (Table S6), which may be attributed to the higher 3-NOP dose used in beef cattle studies (209.75 mg/kg of DM) compared to dairy cattle studies (110 mg/kg of DM). The higher dose seemed to have a greater impact on bacterial composition, or the effects may differ due to variations in rumen microbiomes shaped by genetics, diets, production goals, and physiological characteristics [66–68]. Both dairy and beef cattle fed a high-forage diet showed increases in the molar proportions of propionate and butyrate, but the bacterial taxa responsible for these changes differed between production systems. Propionate-producing bacteria (*Succiniclasticum*, *Succinivibrionaceae* UCG-002, *Prevotella*, and *Anaerovibrio*) were more prevalent in beef cattle, while butyrate-producing bacteria (*Lachnospiraceae* NK3A20 group and *Eubacterium nodatum* group) were more abundant in dairy cattle under 3-NOP supplementation. A previous dairy study suggested that *Clostridium kluyveri* (*C*. *kluyveri*), which can ferment acetate and ethanol to form butyrate and H_2_ [90], may increase in abundance, potentially contributing to an increase in butyrate synthesis when 3-NOP was supplemented [26]. In our study, only Dairy2 study had this species (control: 0.021% vs. 68 mg/kg of DM of 3-NOP: 0.037%, *P* = 0.032, Table S14) increased. The fact that *C*. *kluyveri* and butyrate-producing bacteria increased more in dairy cattle could be linked to the high-forage diet composition and the increased molar proportion of butyrate observed in the Dairy2 study, which is consistent with previous study [26], while Dairy1 which had a high-grain diet did not show an increase in *C*. *kluyveri* or butyrate. Notably, *C*. *kluyveri* was observed only in dairy cattle, suggesting inherent differences in the rumen microbial communities between beef and dairy production systems. The high-forage diets in dairy cattle provide more substrates for butyrate production, where *C*. *kluyveri* [90] contributes to both butyrate synthesis and H_2_ production under 3-NOP supplementation. This H_2_ could be detected by sensory hydrogenase and redirected to alternative pathways such as propionate formation. These findings suggest that both diet composition and cattle type/genetics play key roles in shaping the effects of 3-NOP on microbial composition, metabolite production, and ultimately CH_4_ reduction.

Protozoa have been estimated to contribute between 9 and 37% of ruminal CH_4_ production through their association with methanogens [28, 91, 92], yet they were not accessed for the responses upon 3-NOP treatment. In the present study, comparative analysis of rumen metagenomes showed a decrease in *Isotricha prostoma* (*I*. *prostoma*), a species known to be enriched in high CH_4_-producing communities [93]. *Isotricha* *prostoma* displayed higher hydrogenosome activity [28], and its symbiotic relationship with hydrogenotrophic methanogens contribute to increased CH_4_ emission [94]. This is further supported by Tymensen et al. [94], who identified *Isotricha* as a key protozoan species harboring symbiotic methanogens, including *Mbb* sp. Thus, we speculate that the decreased *I*. *prostoma* may be associated with the reduction of H_2_-utilizing methanogens (*Mbb*. sp.) observed after 3-NOP supplementation. Solomon et al. [93] found that higher abundance of *Isotricha* was linked to lower abundance of *Prevotella* compared to protozoa-free conditions. Consistently, our comparative analysis of rumen metataxonomic showed an increase in *Prevotella* abundance after 3-NOP supplementation suggesting that the decrease in CH_4_ may be due to a reduction in *I*. *prostoma* and an increase in *Prevotella*, which plays a key role in propionate production as an alternative H_2_ sink [83]. In the long-term 3-NOP supplementation trials with beef cattle, we observed increases in *Entodinium longinucleatum* and *Polyplastron multivesiculatum* (*P*. *multivesiculatum*). This may be linked to changes in H_2_ partial pressure caused by 3-NOP, as previous study has shown that *P*. *multivesiculatum* can alter its metabolism in response to such changes [95]. With 3-NOP supplementation decreasing methanogen abundance and increasing H_2_ partial pressure, *P*. *multivesiculatum* may shift towards increased formate production, which is supported by increased formate concentrations observed in the rumen after 3-NOP supplementation [96, 97]. This shift could provide formate-utilizing methanogens such as *Mbb*. *ruminantium* [76] with a competitive advantage over species like *Mbb*. *gottschalkii* that cannot use formate [98]. The differential responses of these species to 3-NOP support this hypothesis, with *Mbb*. *ruminantium* increasing in abundance and *Mbb*. *gottschalkii* decreasing. However, these interactions were only observed in the Beef2 study, suggesting that the effects may be specific to long-term 3-NOP use. Further research, including co-culture experiments with *P*. *multivesiculatum*, *Mbb*. *gottschalkii*, *Mbb*. *ruminantium*, and *Methanosphaera* sp., is needed to confirm whether similar dynamics occur under different conditions.

With 3-NOP-induced the decrease in hydrogenotrophic methanogens (*Mbb*. sp.), we expected an increase in homoacetogens to utilize the excess CO_2_ and H_2_. However, the results showed a decrease in both the molar proportion of acetate and the abundance of Wood-Ljungdahl pathway genes (*fdhF*, *FHS*, *folD*, and *rnfC2*), particularly in *Ruminococcus* (*R*) sp. This was notably evidenced by *R*. *albus*, a known H_2_ and acetate producer [99, 100], whose decreased abundance potentially contributed to decreasing acetate synthesis, even though H_2_ accumulation from 3-NOP treatment could have suppressed its H_2_-producing hydrogenase A1 and potentially favored acetate production [22]. It is noted that a recent study by Ni et al. [101] reported an increase in Wood-Ljungdahl pathway genes after 3-NOP supplementation, contrasting with our findings. This discrepancy likely stems from different analytical approaches: our study used functional annotation of the overall metagenome, while Ni et al. [101] employed MAGs. Additionally, differences in experimental conditions, such as 3-NOP dose and cattle type, may contribute to these divergent outcomes. These conflicting results highlight the complexity of rumen microbial responses to 3-NOP and underscore the need for further investigation, potentially combining both analytical approaches in future studies.

Further analysis of alternative H_2_ sink pathways, such as propionate and butyrate synthesis, and electron transfer processes such as glutamate dehydrogenase/synthase, nitrogen ammonification, and sulfur metabolism revealed better understanding of methanogenesis in the rumen and how it could be impacted by 3-NOP. Previous, CH_4_-reducing studies have shown that as methanogen abundances decrease, propionate- and butyrate-producing bacteria increase, indicating a shift in H_2_ utilization to alternative pathways [26, 102]. Our studies revealed significant changes in hydrogenase abundances, indicating shifts in H_2_ metabolism. The reduction in CH_4_ production and decreased acetate synthesis potentially led to an increase in ruminal H_2_ concentration, as evidenced by a significant reduction in fermentative hydrogenase FeFe A1 and *Ruminococcus* sp., which typically produces H_2_ [22] and an increase in sensory hydrogenase FeFe C2. We observed an enrichment of *ACADS*, which is related to propionate and butyrate production. The main propionate-producing pathways showed contradictory results, with decreased abundance of both *LDH* (lactate pathway) and *sucD* (succinate pathway). While *ACADS* abundance increased, this alone cannot explain the overall propionate metabolism as other major pathways were decreased. Propionate production are competitive pathways for CH_4_ production [103], and butyrate can increase under inhibited methanogenesis by 3-NOP [26], potentially due to redirection of H_2_ and activation of alternative synthesis pathways when CH_4_ production is reduced. It is important to note that our metagenomic analysis only provides insights into genetic potentials based on the relative abundances of genes and taxa, future metatranscriptomic analysis, and/or RT-qPCR as well as protein level measurement are needed to fully understand the functional activities of these taxa and genes.

This study also offers new insights into the potential role of terminal reductases in nitrogen metabolism within the rumen as affected by 3-NOP supplementation. Specifically, 3-NOP, known MCR-inhibitor, can be degraded into 1,3-propanediol and nitrite, the latter also known to inactivate MCR [18]. This potentially reflects the continuous degradation of 3-NOP in the rumen, resulting in ongoing nitrite production. Our comparative analysis revealed an increase in the nitrite reductase gene (*nrfA*) only in beef cattle, with one dairy study (Dairy1) also showing a significant increase. Notably, *nrfA* was primarily encoded by *Prevotella* sp. and *Bacteroides* sp., with *Prevotella* sp. showing greater enrichment in beef cattle than dairy cattle, suggesting its crucial role in enhancing nitrite reduction capacity and may explain the more pronounced *nrfA* increase in beef cattle. However, given the low concentrations of nitrite generated by 3-NOP metabolism (approximately 1.5 µM, [104]), the direct impact of *nrfA* increase on MCR inhibition may be limited, as supported by Duin et al. [18], who demonstrated slower MCR inhibition by nitrite compared to 3-NOP in pure culture studies. Although nitrite can impact methanogen abundance [105] and serve as an electron sink through NH_3_ conversion [65], the low concentrations from 3-NOP metabolism indicate minimal contribution to CH_4_ reduction. This is consistent with no significant changes in NH_3_ concentration across our studies, aligning with other 3-NOP studies [14, 102]. During nitrogen assimilation in the rumen, glutamate dehydrogenase (*gdhA*) facilitates NH_3_ to L-glutamate conversion, coupled with reductant disposal through the oxidation of NADPH to NADP^+^ [106]. We found that 79 genomes (76 bacterial and 3 archaeal) encoded this gene, primarily in *Prevotella* sp. and *Ruminococcus* sp., consistent with previous research [107, 108]. Despite *gdhA* reduction after 3-NOP supplementation, NH_3_ concentration remained unchanged, highlighting a potential discrepancy that requires further investigation.

**Fig. 8:**
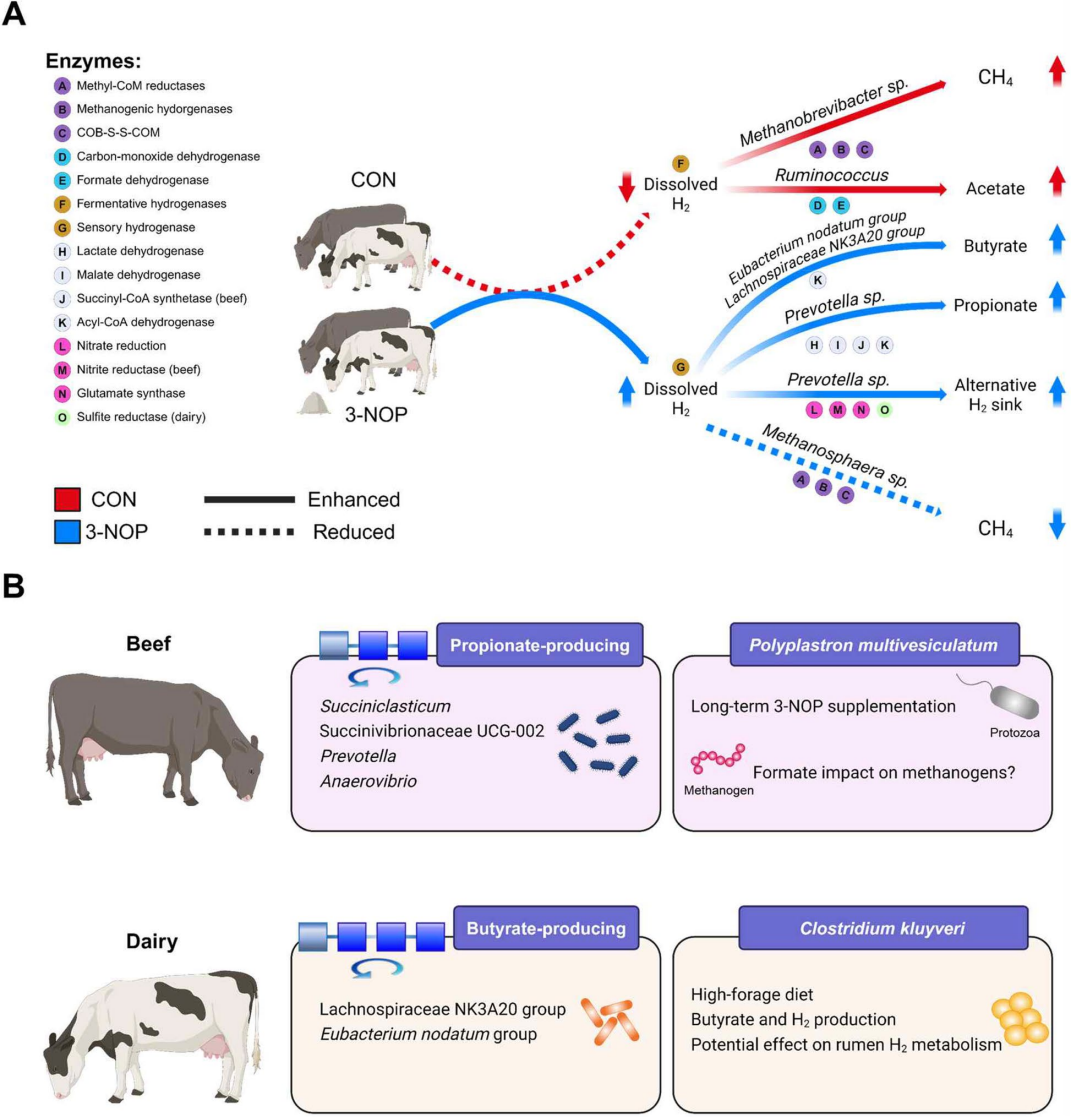
Proposed hydrogen metabolism model through comparative analysis of 3-nitrooxypropanol (3-NOP) supplementation in beef and dairy cattle diets. **A** indicates diverse rumen enzymes related to hydrogenase, reductase, and electron transferases between control and 3-NOP groups. **B** indicates the differential responses of rumen microbes between beef and dairy cattle. Solid and dotted lines indicate trends of enhanced and reduced pathways, respectively. Upward arrows indicate increased metabolites, while downward arrows indicate decreased metabolites. *H*_2_ hydrogen, *CH*_4_ methane

Based on our primary findings, we proposed H_2_ metabolism models and differential microbial responses between beef and dairy cattle after 3-NOP supplementation (Fig. 8). A notable difference between beef and dairy cattle was the enrichment of *MTD* in beef cattle, a key enzyme involved in hydrogenotrophic methanogenesis. Additionally, the interaction between *P*. *multivesiculatum*, *Mbb*. *ruminantium*, and *Mbb*. *gottschalkii* in beef cattle suggests a pivotal role for formate metabolism during long-term 3-NOP supplementation, as *Mbb*. *ruminantium* can utilize formate for methanogenesis, unlike *Mbb*. *gottschalkii*. In dairy cattle, *C*. *kluyveri* was exclusively enriched under high-forage diets, while beef cattle showed higher abundance of propionate-producing bacteria, suggesting potential influence of diet composition on microbial responses to 3-NOP supplementation. These models suggest that 3-NOP supplementation decreases or increases hydrogenotrophic methanogenesis, VFA production, and alternative H_2_ sink pathways, as evidenced by significant changes in rumen microbial composition and associated functions in both beef and dairy cattle. The limitations of this study include the use of 3-NOP doses (beef: 53, 161, 280, and 345 mg/kg of DM and dairy: 68, 130, and 132 mg/kg of DM) that were above current commercial recommendations, although these data remain valuable for understanding dose-dependent microbial responses in the rumen. Additionally, the use of a reference database with limited availability of ruminant-specific MAGs [57] may have reduced the taxonomic resolution in the metagenome analysis. However, it is important to note that our metagenomic analysis provides insights only into the genetic potentials. Metatranscriptomic analysis and validation using RT-qPCR are needed to fully understand the functional activities of these microbial communities and to validate these findings in future 3-NOP trials. While we present this as a preliminary report based on a comparative analysis of microbial composition and function between beef and dairy cattle supplemented with 3-NOP, we believe these findings provide valuable insights for future research in this field. Given that metagenomic studies involving 3-NOP remain scarce, this study is the first to comparatively analyze datasets from both beef and dairy cattle. Future meta-analyses will be necessary to validate and expand upon the patterns observed in this study as more datasets become available.

## Conclusion

This comparative analysis of rumen microbiome data from two beef and two dairy studies revealed key insights into how 3-NOP supplementation affected CH_4_ emissions by suppressing methanogenesis and restructuring rumen microbiome functions. While previous studies have demonstrated 3-NOP's effectiveness, our integrated analysis with doses ranging from 53 to 345 mg/kg DM in beef and 68–132 mg/kg DM in dairy cattle uniquely revealed cattle type-specific responses, which may contribute to our understanding of how to optimize its application in different production systems. Importantly, our findings support the direct impact of 3-NOP on specific methanogens and provide the evidence suggesting potential for intermittent use or dosage adjustments in CH_4_ mitigation strategies without long-term disruption of rumen microbial balance. We conclude that 3-NOP modulated rumen VFA production pathways, H_2_ metabolism, and electron transfer, leading to methanogenesis inhibition in both cattle types. The observed differences between cattle types emphasize the need for further research to understand the underlying factors influenced by diet composition and 3-NOP dose. Furthermore, the observed bacterial responses to 3-NOP suggest that future research could explore potential synergistic effects between 3-NOP and specific bacterial taxa as potential probiotics (e.g., *Prevotella sp*. and *C*. *kluyveri*) that showed positive responses in our study, which might lead to improved strategies for CH_4_ mitigation in commercial settings. More integrated research is needed to guide its adoption and translation into practice, potentially leading to more environmentally friendly and productive outcomes in both beef and dairy cattle.

## Supplementary Information

Additional file 1: Figure S1. Schematic representation of the four in vivo trials used in the comparative analysis, including short-term and long-term 3-NOP supplementation studies in beef and dairy cattle (Beef1: Romero-Perez et al., 2014 [9]; Beef2: Romero-Perez et al., 2015 [10]; Dairy1: Haisan et al., 2014 [15]; Dairy2: Haisan et al., 2017 [16]). Figure S2. Effect of short-term 3-nitrooxypropanol (3-NOP) supplementation on the abundance of A bacterial, B archaeal, and C protozoal taxa in beef cattle. *3-NOP dose level information: con: 0, low: 53, med: 161, high: 345 mg/kg of DM. Others indicates taxa with less than 5% abundance; UCF: uncultured family-level; UCG: uncultured genus-level; UG: unclassified genus-level. Figure S3. Effect of long-term 3-nitrooxypropanol (3-NOP) supplementation on the abundance of A bacterial, B archaeal, and C protozoal taxa in beef cattle. *3-NOP dose level information: con: 0, high: 280 mg/kg of DM. Others indicates taxa with less than 5% abundance; UCG: uncultured genus-level; UG: unclassified genus-level; recov: recovery period. Figure S4. Effect of 3-nitrooxypropanol (3-NOP) supplementation on the abundance of A bacterial, B archaeal, and C protozoal taxa in dairy cattle. *3-NOP dose level information: con: 0, high: 130 mg/kg of DM. Others indicates taxa with less than 5% abundance; UCG: uncultured genus-level; UG: unclassified genus-level. Figure S5. Dose response effect of 3-nitrooxypropanol (3-NOP) supplementation on the abundance of A bacterial, B archaeal, and C protozoal taxa in dairy cattle. *3-NOP dose level information: con: 0, low: 68, high: 132 mg/kg of DM. Others indicates taxa with less than 5% abundance; UCG: uncultured genus-level; UG: unclassified genus-level. Figure S6. Alpha diversity and beta diversity analysis of rumen microbiota before and after batch correction. Alpha diversity was measured by Shannon index in A bacteria, B archaea, and C protozoa of control and 3-NOP treated groups. *P* values were calculated using Wilcoxon rank sum test, with significance set at *P* < 0.05. Principal coordinate analysis (PCoA) plots of D bacteria, E archaea, and F protozoa were based on Bray-Curtis distance. *P* values were calculated with PERMANOVA (999 permutations). Figure S7. Alpha diversity (Chao1 and evenness) was measured from metataxonomic data (A Beef1, B Beef2, C Dariy1; and D Dairy2). The Kruskal-Wallis test with Dunn’s post-hoc test was applied to Beef1 and Dairy2, while the Wilcoxon test was used for Beef2 and Dairy1. *P*-values were FDR-adjusted, with < 0.05 considered statistically significant. Figure S8. Beta diversity was measured from metataxonomic data (A Beef1, B Beef2, C Dariy1; and D Dairy2). Principal coordinate analysis (PCoA) plots of bacteria, archaea, and protozoa were based on Weighted Unifrac distance. *P* values were calculated with PERMANOVA (999 permutations). Figure S9. Effect of short-term 3-nitrooxypropanol (3-NOP) supplementation on the distributions of hydrogenases and associated terminal reductases in beef cattle. A indicates distributions (in phyla with hydrogenase-encoding genes) of fermentative hydrogenases (group A1, A2, and B FeFe-hydrogenases), bifurcating hydrogenases (group A3 FeFe-hydrogenases), respiratory hydrogenases (group 1i NiFe-hydrogenases), methanogenic hydrogenases (Fe-hydrogenases, group 3a, 3c, 4 h, and 4i NiFe-hydrogenases), sensory hydrogenases (group C FeFe-hydrogenases), and energy-converting hydrogenases (bidirectional; group 4e and 4 g NiFe-hydrogenases). B indicates the H_2_ uptake pathway includes genes involved in sulfidogenesis (*asrA*, alternative sulfite reductase; *dmsA*, DMSO, and TMAO reductase), fumarate reduction (*frdA*, fumarate reductase), nitrate ammonification (*nrfA*, ammonia-forming nitrite reductase), and *nifH*, nitrogenase and methanogenesis (*mcrA*, methyl-CoM reductase). Only genes with an average relative abundance (CPM: count per million) > 1 are shown. Data with error bars are indicated as mean ± standard error. Asterisks indicate significance: *P < 0.1, **P < 0.05. Figure S10. Effect of long-term 3-nitrooxypropanol (3-NOP) supplementation on the distributions of hydrogenases and associated terminal reductases in beef cattle. A indicates distributions (in phyla with hydrogenase-encoding genes) of fermentative hydrogenases (group A1, A2, and B FeFe-hydrogenases), bifurcating hydrogenases (group A3 FeFe-hydrogenases), respiratory hydrogenases (group 1i NiFe-hydrogenases), methanogenic hydrogenases (Fe-hydrogenases, group 3a, 3c, 4 h, and 4i NiFe-hydrogenases), sensory hydrogenases (group C FeFe-hydrogenases), *HydB*, hydrogenase-associated diaphorase, energy-converting hydrogenases (bidirectional; group 4e and 4g NiFe-hydrogenases). B indicates the H_2_ uptake pathway includes genes involved in sulfidogenesis (*asrA*, alternative sulfite reductase; *dmsA*, DMSO, and TMAO reductase), fumarate reduction (*frdA*, fumarate reductase), *nifH*, nitrogenase, nitrate ammonification (*nrfA*, ammonia-forming nitrite reductase), methanogenesis (*mcrA*, methyl-CoM reductase). Only genes with a relative abundance (CPM: count per million) > 1 are shown. Data with error bars are indicated as mean ± standard error. Asterisks indicate significance: *P < 0.1, **P < 0.05. Figure S11. Effect of 3-nitrooxypropanol (3-NOP) supplementation on the distributions of hydrogenases and associated terminal reductases in dairy cattle. A indicates distributions (in phyla with hydrogenase-encoding genes) of fermentative hydrogenases (group A1, A2, and B FeFe-hydrogenases), bifurcating hydrogenases (group A3 FeFe-hydrogenases), respiratory hydrogenases (group 1d and 1i NiFe-hydrogenases), methanogenic hydrogenases (Fe-hydrogenases, group 3a, 3c, 4 h, and 4i NiFe-hydrogenases), sensory hydrogenases (group C FeFe-hydrogenases), *HydB*, hydrogenase-associated diaphorase, and energy-converting hydrogenases (bidirectional; group 4e and 4 g NiFe-hydrogenases). B indicates the H_2_ uptake pathway includes genes involved in sulfidogenesis (*asrA*, alternative sulfite reductase), aerobic respiration (*cydA*, cytochrome bd oxidase), fumarate reduction (*frdA*, fumarate reductase), *nifH*, nitrogenase, nitrate ammonification (*narG*, dissimilatory nitrate reductase; *nrfA*, ammonia-forming nitrite reductase), and methanogenesis (*mcrA*, methyl-CoM reductase). Only genes with a relative abundance (CPM: count per million) > 1 are shown. Data with error bars are indicated as mean ± standard error. Asterisks indicate significance: *P < 0.1, **P < 0.05. Figure S12. Dose response effect of 3-nitrooxypropanol (3-NOP) supplementation on the distributions of hydrogenases and associated terminal reductases in dairy cattle. A indicates distributions (in phyla with hydrogenase-encoding genes) of fermentative hydrogenases (group A1, A2, and B FeFe-hydrogenases), bifurcating hydrogenases (group A3 FeFe-hydrogenases), respiratory hydrogenases (group 1i NiFe-hydrogenases), methanogenic hydrogenases (Fe-hydrogenases, group 3a, 3c, 4 h, and 4i NiFe-hydrogenases), sensory hydrogenases (group C FeFe-hydrogenases), *HydB*, hydrogenase-associated diaphorase, and energy-converting hydrogenases (bidirectional; group 4e and 4g NiFe-hydrogenases). B indicates the H_2_ uptake pathway includes genes involved in sulfidogenesis (*asrA*, alternative sulfite reductase; *dsrA*, dissimilatory sulfite reductase), fumarate reduction (*frdA*, fumarate reductase), and *nifH*, nitrogenase, nitrate ammonification (*nrfA*, ammonia-forming nitrite reductase), and methanogenesis (*mcrA*, methyl-CoM reductase). Only genes with an average relative abundance (CPM: count per million) > 1 are shown. Data with error bars are indicated as mean ± standard error. Asterisks indicate significance: *P < 0.1, **P < 0.05. Figure S13. Effect of 3-nitrooxypropanol (3-NOP) supplementation on the distributions of hydrogenases, associated terminal reductases, and electron transferases in beef cattle. A results were obtained from a comparative analysis using MMUPHin and further validated by MaAsLin2 analysis. The gray, yellow, and skyblue strips represent hydrogenases, associated terminal reductases, and electron transferases, respectively. The comparative analysis was conducted with a threshold of Q < 0.05 and cross-verified by MaAsLin2 with Q < 0.05. CPM: count per million; DM: dry matter. Figure S14. Effect of 3-nitrooxypropanol (3-NOP) supplementation on the distributions of hydrogenases and associated terminal reductases in dairy cattle. A results were obtained from a comparative analysis using MMUPHin and further validated by MaAsLin2 analysis. The gray and skyblue strips represent hydrogenases and associated terminal reductases, respectively. The comparative analysis was conducted with a threshold of Q < 0.05 and cross-verified by MaAsLin2 with Q < 0.05. CPM: count per million; DM: dry matter.

Additional file 2: Table S1. Ingredient and chemical composition of the basal diet in beef study 1. Table S2. Ingredient and chemical composition of the basal diet in beef study 2. Table S3. Ingredient and chemical composition of the basal diet in dairy study 1. Table S4. Ingredient and chemical composition of the basal diet in dairy study 2.

Additional file 3: Table S5. Summary of sequence data generated from rumen samples after 3-NOP supplementation. Table S6. Comparative analysis heterogeneity test results and differentially abundant rumen microbial taxa after 3-NOP supplementation based on metataxonomic analysis. Table S7. Significantly different of rumen microbial taxa in the metataxonomic analysis of beef study 1 after 3-NOP supplementation. Table S8. Significantly different of rumen microbial taxa in the metataxonomic analysis of beef study 2 after 3-NOP supplementation. Table S9. Significantly different relative abundance (%) of rumen microbial taxa in the metataxonomic analysis of beef study 2 during the recovery phase after 3-NOP supplementation. Table S10. Significantly different rumen microbial taxa in the metataxonomic analysis of dairy study 1 after 3-NOP supplementation. Table S11. Significantly different rumen microbial taxa in the metataxonomic analysis of dairy study 2 after 3-NOP supplementation. Table S12. Significantly different relative abundance (%) of rumen microbial taxa in the metagenomic analysis of beef study 1 after 3-NOP supplementation. Table S13. Significantly different relative abundance (%) of rumen microbial taxa in the metagenomic analysis of beef study 2 after 3-NOP supplementation. Table S14. Significantly different relative abundance (%) of rumen microbial taxa in the metagenomic analysis of dairy study 1 after 3-NOP supplementation. Table S15. Significantly different relative abundance (%) of rumen microbial taxa in the metagenomic analysis of dairy study 2 after 3-NOP supplementation. Table S16. Comparative analysis heterogeneity test results and differentially abundant rumen microbial taxa after 3-NOP supplementation based on metagenomic analysis. Table S17. Comparative analysis heterogeneity test results and significant differences of 3-nitrooxypropanol (3-NOP) supplementation on hydrogenases, terminal reductases, and electron transferases. Table S18. Summary of genes related to hydrogen sink and electron transferases after 3-NOP supplementation. Table S19. Summary of modules related to methane, volatile fatty acid, and nitrogen after 3-NOP supplementation. Table S20. Methyl coenzyme M reductase abundance analyzed by metagenomics after 3-NOP supplementation.

## Data Availability

Pyrosequencing gene sequencing data of rumen microbiota are available at the National Center for Biotechnology Information (NCBI) under project number PRJNA1150225 (Beef1), PRJNA1150246 (Beef2), PRJNA1150253 (Dairy1), and PRJNA1150254 (Dairy2). Metagenomic sequencing data of ruminal microbiota are available at NCBI (project number PRJNA1150266).
