# Supplementary material for: Comparative analysis of rumen metagenomes with dietary supplementation of 3-nitrooxypropanol revealed divergent modes of action in hydrogen metabolism and reductant pathways between beef and dairy cattle
Source: Microbiome. 2026 Feb 19;14:72. doi: 10.1186/s40168-025-02201-y (PMC12918512; doi:10.1186/s40168-025-02201-y)
Supplement: Supplementary file 2 — Additional file 1: Figure S1. Schematic representation of the four in vivo trials used in the comparative analysis, including short-term and long-term 3-NOP supplementation studies in beef and dairy cattle (Beef1: Romero-Perez et al., 2014 [9]; Beef2: Romero-Perez et al., 2015 [10]; Dairy1: Haisan et al., 2014 [15]; Dairy2: Haisan et al., 2017 [16]). Figure S2. Effect of short-term 3-nitrooxypropanol (3-NOP) supplementation on the abundance of A bacterial, B archaeal, and C protozoal taxa in beef cattle. *3-NOP dose level information: con: 0, low: 53, med: 161, high: 345 mg/kg of DM. Others indicates taxa with less than 5% abundance; UCF: uncultured family-level; UCG: uncultured genus-level; UG: unclassified genus-level. Figure S3. Effect of long-term 3-nitrooxypropanol (3-NOP) supplementation on the abundance of A bacterial, B archaeal, and C protozoal taxa in beef cattle. *3-NOP dose level information: con: 0, high: 280 mg/kg of DM. Others indicates taxa with less than 5% abundance; UCG: uncultured genus-level; UG: unclassified genus-level; recov: recovery period. Figure S4. Effect of 3-nitrooxypropanol (3-NOP) supplementation on the abundance of A bacterial, B archaeal, and C protozoal taxa in dairy cattle. *3-NOP dose level information: con: 0, high: 130 mg/kg of DM. Others indicates taxa with less than 5% abundance; UCG: uncultured genus-level; UG: unclassified genus-level. Figure S5. Dose response effect of 3-nitrooxypropanol (3-NOP) supplementation on the abundance of A bacterial, B archaeal, and C protozoal taxa in dairy cattle. *3-NOP dose level information: con: 0, low: 68, high: 132 mg/kg of DM. Others indicates taxa with less than 5% abundance; UCG: uncultured genus-level; UG: unclassified genus-level. Figure S6. Alpha diversity and beta diversity analysis of rumen microbiota before and after batch correction. Alpha diversity was measured by Shannon index in A bacteria, B archaea, and C protozoa of control and 3-NOP treated groups. P values were calculat [file 40168_2025_2201_MOESM1_ESM.zip › Supplemental figures/Choi et al. FigureS1.pdf]

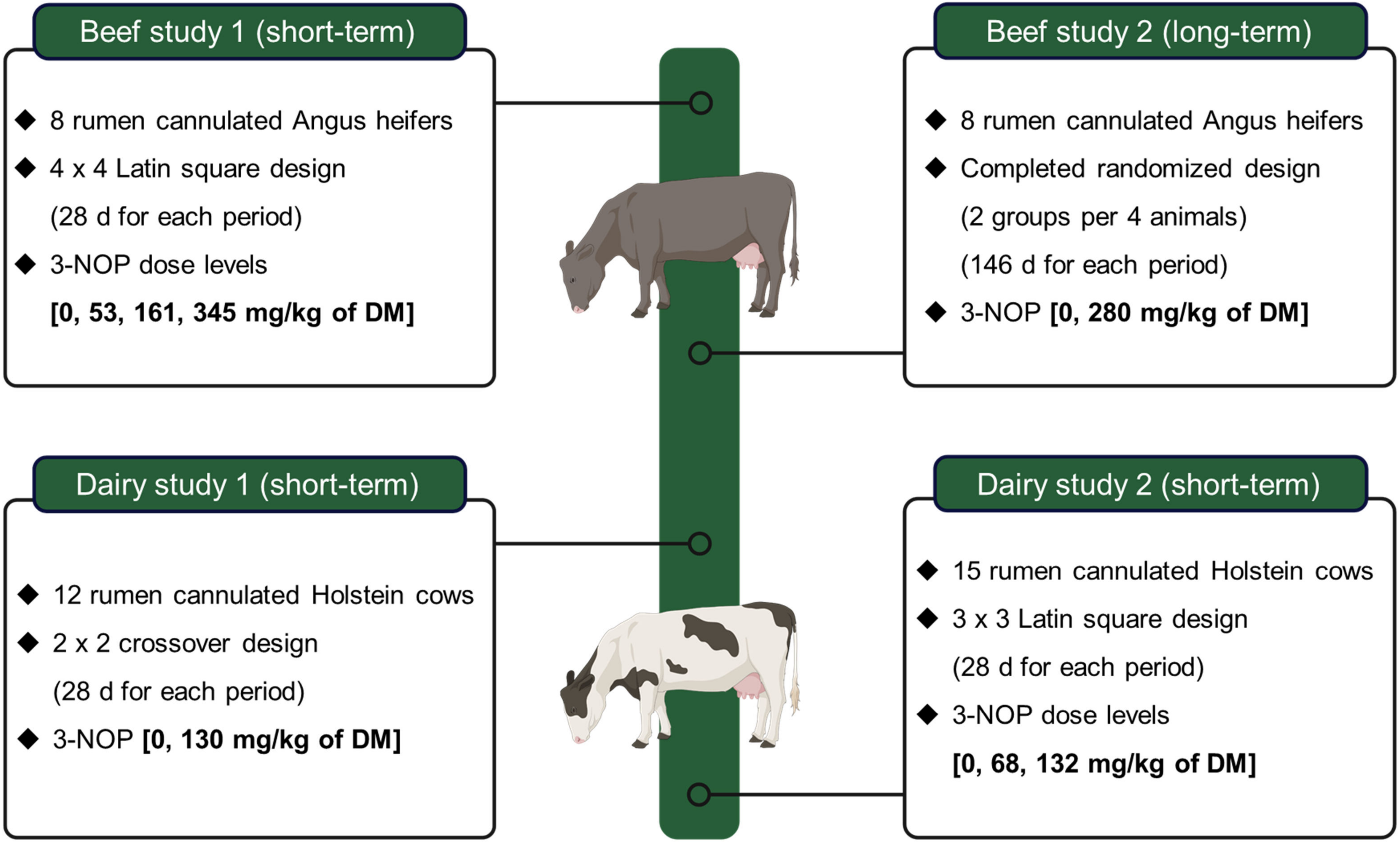
Beef study 1 (short-term)
8 rumen cannulated Angus heifers
4 x 4 Latin square design
(28 d for each period)
3-NOP dose levels
[0, 53, 161, 345 mg/kg of DM]
Beef study 2 (long-term)
8 rumen cannulated Angus heifers
Completed randomized design
(2 groups per 4 animals)
(146 d for each period)
3-NOP [0, 280 mg/kg of DM]
Dairy study 1 (short-term)
12 rumen cannulated Holstein cows
2 x 2 crossover design
(28 d for each period)
3-NOP [0, 130 mg/kg of DM]
Dairy study 2 (short-term)
15 rumen cannulated Holstein cows
3 x 3 Latin square design
(28 d for each period)
3-NOP dose levels
[0, 68, 132 mg/kg of DM]
