# Supplementary figures and images for: Comparative analysis of rumen metagenomes with dietary supplementation of 3-nitrooxypropanol revealed divergent modes of action in hydrogen metabolism and reductant pathways between beef and dairy cattle
Source: Microbiome. 2026 Feb 19;14:72. doi: 10.1186/s40168-025-02201-y (PMC12918512; doi:10.1186/s40168-025-02201-y)

Beef 2

A Hydrogenase

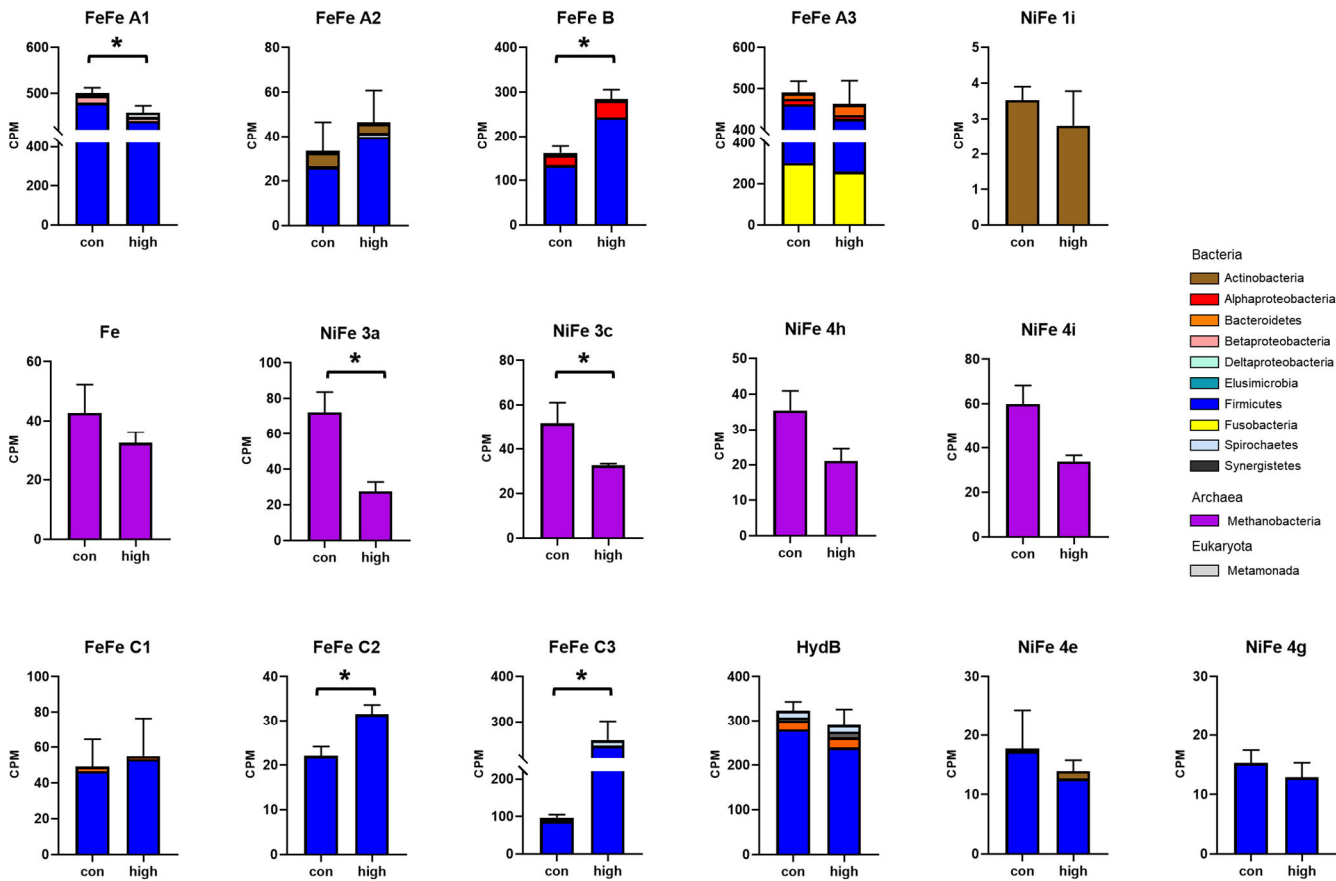

B Terminal reductase

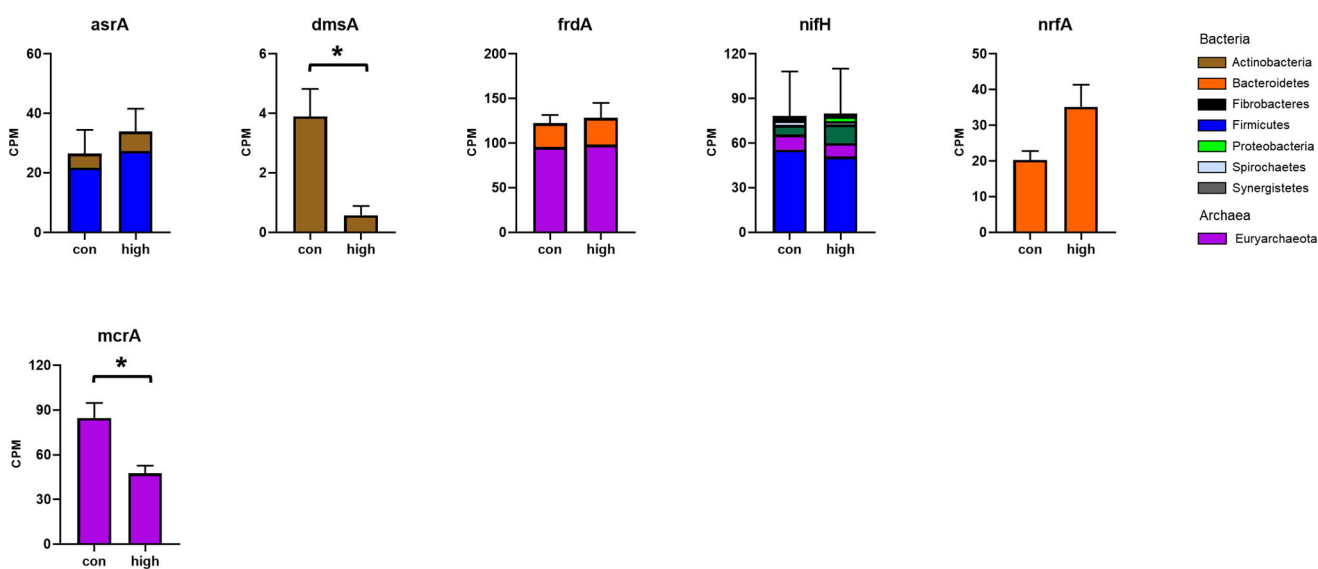

Supplement: Supplementary file 2 — Additional file 1: Figure S1. Schematic representation of the four in vivo trials used in the comparative analysis, including short-term and long-term 3-NOP supplementation studies in beef and dairy cattle (Beef1: Romero-Perez et al., 2014 [9]; Beef2: Romero-Perez et al., 2015 [10]; Dairy1: Haisan et al., 2014 [15]; Dairy2: Haisan et al., 2017 [16]). Figure S2. Effect of short-term 3-nitrooxypropanol (3-NOP) supplementation on the abundance of A bacterial, B archaeal, and C protozoal taxa in beef cattle. *3-NOP dose level information: con: 0, low: 53, med: 161, high: 345 mg/kg of DM. Others indicates taxa with less than 5% abundance; UCF: uncultured family-level; UCG: uncultured genus-level; UG: unclassified genus-level. Figure S3. Effect of long-term 3-nitrooxypropanol (3-NOP) supplementation on the abundance of A bacterial, B archaeal, and C protozoal taxa in beef cattle. *3-NOP dose level information: con: 0, high: 280 mg/kg of DM. Others indicates taxa with less than 5% abundance; UCG: uncultured genus-level; UG: unclassified genus-level; recov: recovery period. Figure S4. Effect of 3-nitrooxypropanol (3-NOP) supplementation on the abundance of A bacterial, B archaeal, and C protozoal taxa in dairy cattle. *3-NOP dose level information: con: 0, high: 130 mg/kg of DM. Others indicates taxa with less than 5% abundance; UCG: uncultured genus-level; UG: unclassified genus-level. Figure S5. Dose response effect of 3-nitrooxypropanol (3-NOP) supplementation on the abundance of A bacterial, B archaeal, and C protozoal taxa in dairy cattle. *3-NOP dose level information: con: 0, low: 68, high: 132 mg/kg of DM. Others indicates taxa with less than 5% abundance; UCG: uncultured genus-level; UG: unclassified genus-level. Figure S6. Alpha diversity and beta diversity analysis of rumen microbiota before and after batch correction. Alpha diversity was measured by Shannon index in A bacteria, B archaea, and C protozoa of control and 3-NOP treated groups. P values were calculat [file 40168_2025_2201_MOESM1_ESM.zip › Supplemental figures/Choi et al. FigureS10.pdf]

Dairy 1

A Hydrogenase

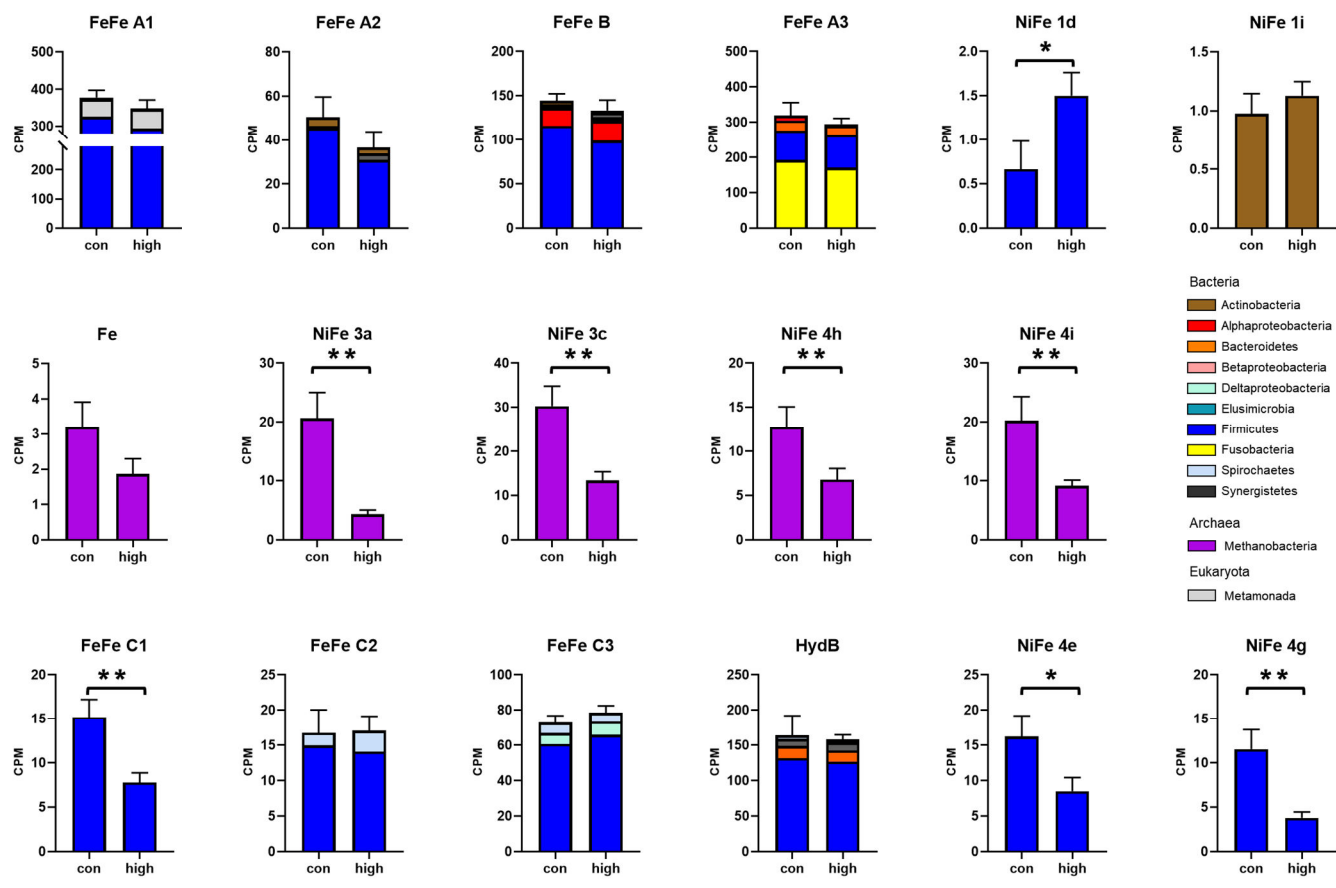

B Terminal reductase

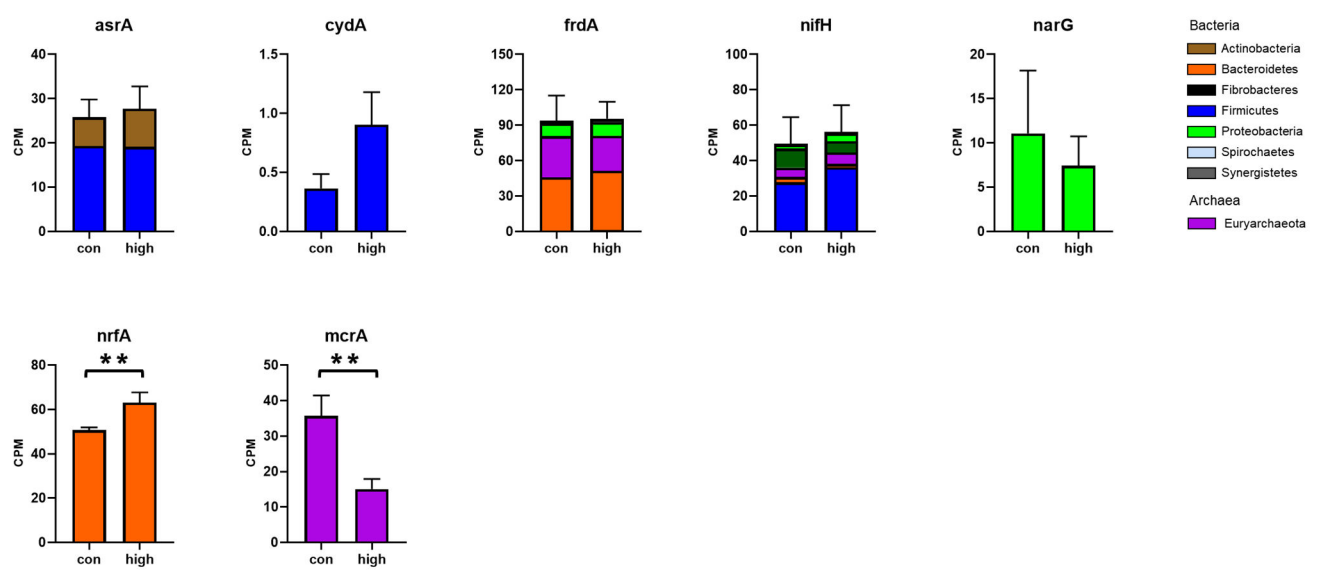

Supplement: Supplementary file 2 — Additional file 1: Figure S1. Schematic representation of the four in vivo trials used in the comparative analysis, including short-term and long-term 3-NOP supplementation studies in beef and dairy cattle (Beef1: Romero-Perez et al., 2014 [9]; Beef2: Romero-Perez et al., 2015 [10]; Dairy1: Haisan et al., 2014 [15]; Dairy2: Haisan et al., 2017 [16]). Figure S2. Effect of short-term 3-nitrooxypropanol (3-NOP) supplementation on the abundance of A bacterial, B archaeal, and C protozoal taxa in beef cattle. *3-NOP dose level information: con: 0, low: 53, med: 161, high: 345 mg/kg of DM. Others indicates taxa with less than 5% abundance; UCF: uncultured family-level; UCG: uncultured genus-level; UG: unclassified genus-level. Figure S3. Effect of long-term 3-nitrooxypropanol (3-NOP) supplementation on the abundance of A bacterial, B archaeal, and C protozoal taxa in beef cattle. *3-NOP dose level information: con: 0, high: 280 mg/kg of DM. Others indicates taxa with less than 5% abundance; UCG: uncultured genus-level; UG: unclassified genus-level; recov: recovery period. Figure S4. Effect of 3-nitrooxypropanol (3-NOP) supplementation on the abundance of A bacterial, B archaeal, and C protozoal taxa in dairy cattle. *3-NOP dose level information: con: 0, high: 130 mg/kg of DM. Others indicates taxa with less than 5% abundance; UCG: uncultured genus-level; UG: unclassified genus-level. Figure S5. Dose response effect of 3-nitrooxypropanol (3-NOP) supplementation on the abundance of A bacterial, B archaeal, and C protozoal taxa in dairy cattle. *3-NOP dose level information: con: 0, low: 68, high: 132 mg/kg of DM. Others indicates taxa with less than 5% abundance; UCG: uncultured genus-level; UG: unclassified genus-level. Figure S6. Alpha diversity and beta diversity analysis of rumen microbiota before and after batch correction. Alpha diversity was measured by Shannon index in A bacteria, B archaea, and C protozoa of control and 3-NOP treated groups. P values were calculat [file 40168_2025_2201_MOESM1_ESM.zip › Supplemental figures/Choi et al. FigureS11.pdf]

# Dairy 2

## A Hydrogenase

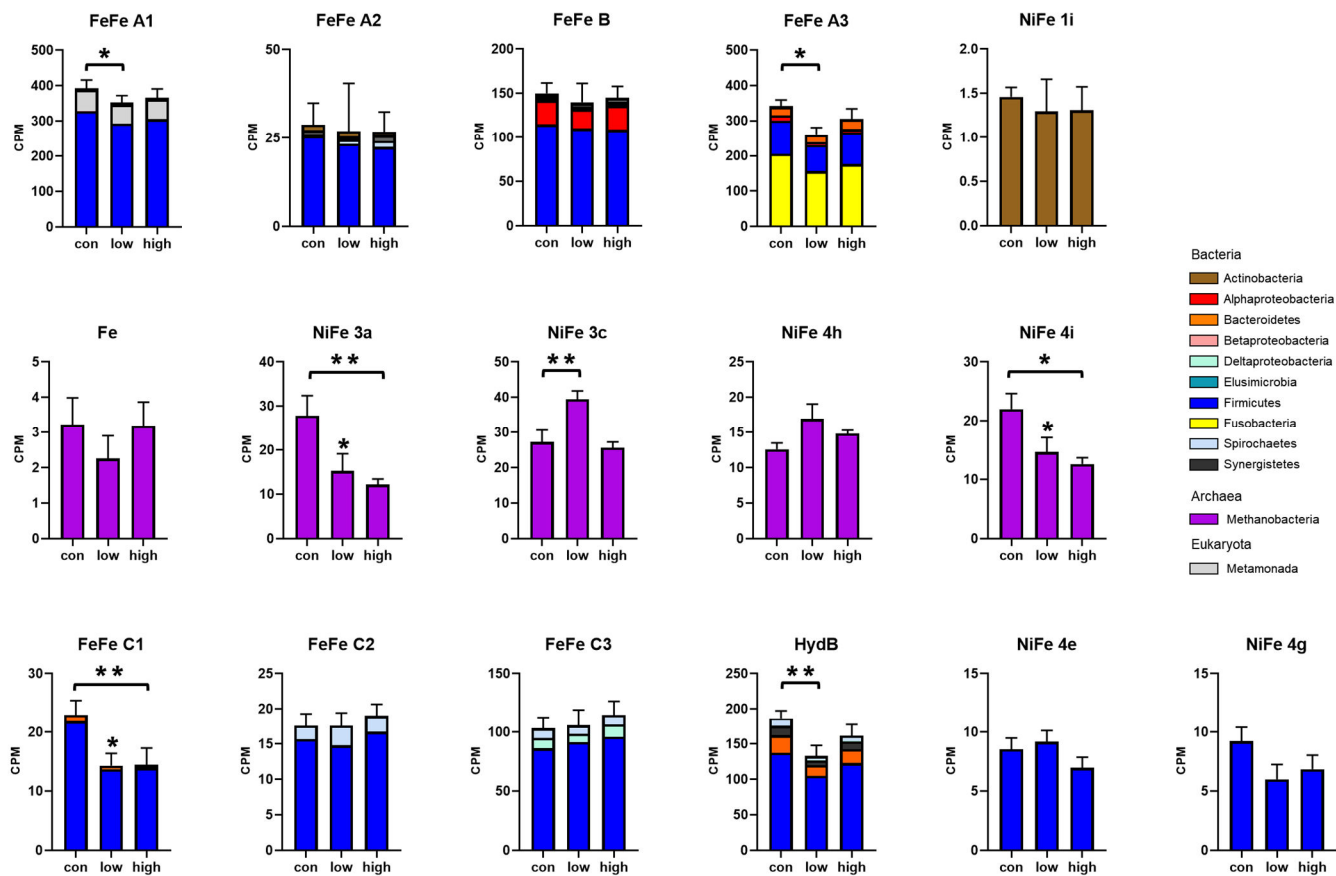

## B Terminal reductase

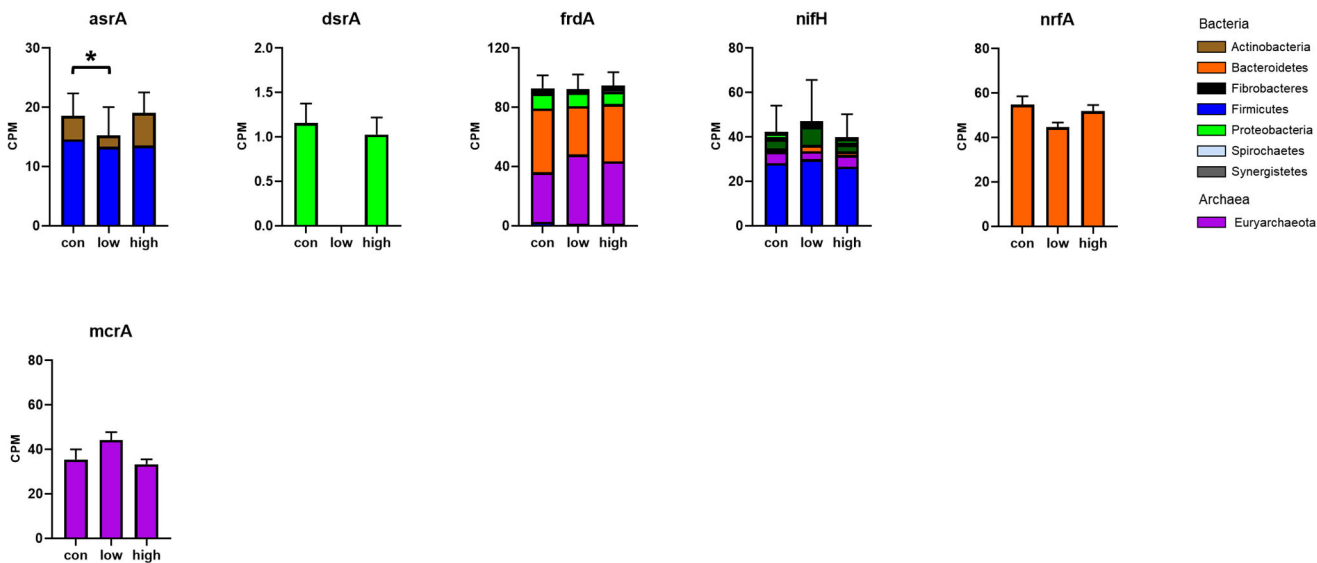

Supplement: Supplementary file 2 — Additional file 1: Figure S1. Schematic representation of the four in vivo trials used in the comparative analysis, including short-term and long-term 3-NOP supplementation studies in beef and dairy cattle (Beef1: Romero-Perez et al., 2014 [9]; Beef2: Romero-Perez et al., 2015 [10]; Dairy1: Haisan et al., 2014 [15]; Dairy2: Haisan et al., 2017 [16]). Figure S2. Effect of short-term 3-nitrooxypropanol (3-NOP) supplementation on the abundance of A bacterial, B archaeal, and C protozoal taxa in beef cattle. *3-NOP dose level information: con: 0, low: 53, med: 161, high: 345 mg/kg of DM. Others indicates taxa with less than 5% abundance; UCF: uncultured family-level; UCG: uncultured genus-level; UG: unclassified genus-level. Figure S3. Effect of long-term 3-nitrooxypropanol (3-NOP) supplementation on the abundance of A bacterial, B archaeal, and C protozoal taxa in beef cattle. *3-NOP dose level information: con: 0, high: 280 mg/kg of DM. Others indicates taxa with less than 5% abundance; UCG: uncultured genus-level; UG: unclassified genus-level; recov: recovery period. Figure S4. Effect of 3-nitrooxypropanol (3-NOP) supplementation on the abundance of A bacterial, B archaeal, and C protozoal taxa in dairy cattle. *3-NOP dose level information: con: 0, high: 130 mg/kg of DM. Others indicates taxa with less than 5% abundance; UCG: uncultured genus-level; UG: unclassified genus-level. Figure S5. Dose response effect of 3-nitrooxypropanol (3-NOP) supplementation on the abundance of A bacterial, B archaeal, and C protozoal taxa in dairy cattle. *3-NOP dose level information: con: 0, low: 68, high: 132 mg/kg of DM. Others indicates taxa with less than 5% abundance; UCG: uncultured genus-level; UG: unclassified genus-level. Figure S6. Alpha diversity and beta diversity analysis of rumen microbiota before and after batch correction. Alpha diversity was measured by Shannon index in A bacteria, B archaea, and C protozoa of control and 3-NOP treated groups. P values were calculat [file 40168_2025_2201_MOESM1_ESM.zip › Supplemental figures/Choi et al. FigureS12.pdf]

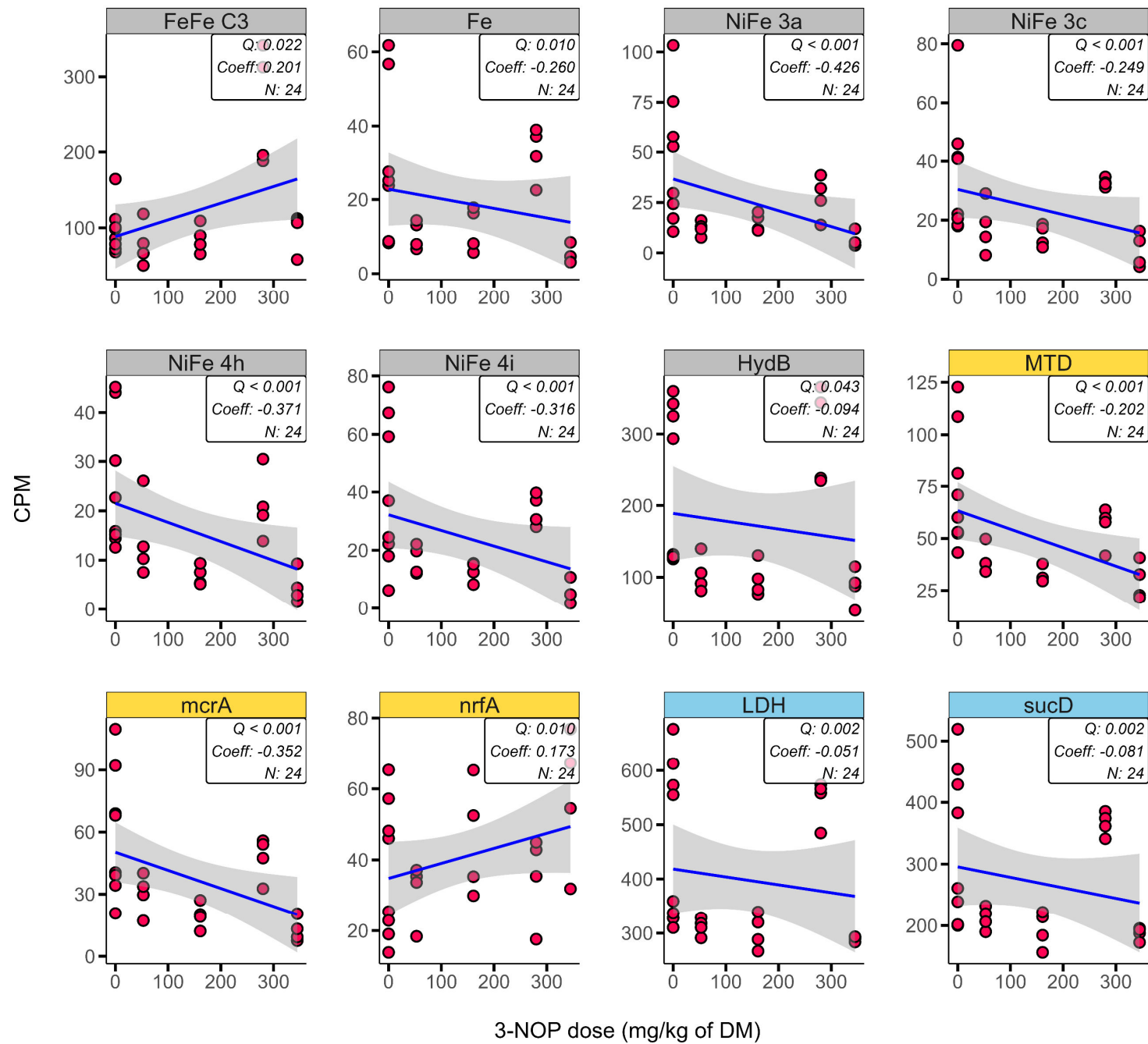

Supplement: Supplementary file 2 — Additional file 1: Figure S1. Schematic representation of the four in vivo trials used in the comparative analysis, including short-term and long-term 3-NOP supplementation studies in beef and dairy cattle (Beef1: Romero-Perez et al., 2014 [9]; Beef2: Romero-Perez et al., 2015 [10]; Dairy1: Haisan et al., 2014 [15]; Dairy2: Haisan et al., 2017 [16]). Figure S2. Effect of short-term 3-nitrooxypropanol (3-NOP) supplementation on the abundance of A bacterial, B archaeal, and C protozoal taxa in beef cattle. *3-NOP dose level information: con: 0, low: 53, med: 161, high: 345 mg/kg of DM. Others indicates taxa with less than 5% abundance; UCF: uncultured family-level; UCG: uncultured genus-level; UG: unclassified genus-level. Figure S3. Effect of long-term 3-nitrooxypropanol (3-NOP) supplementation on the abundance of A bacterial, B archaeal, and C protozoal taxa in beef cattle. *3-NOP dose level information: con: 0, high: 280 mg/kg of DM. Others indicates taxa with less than 5% abundance; UCG: uncultured genus-level; UG: unclassified genus-level; recov: recovery period. Figure S4. Effect of 3-nitrooxypropanol (3-NOP) supplementation on the abundance of A bacterial, B archaeal, and C protozoal taxa in dairy cattle. *3-NOP dose level information: con: 0, high: 130 mg/kg of DM. Others indicates taxa with less than 5% abundance; UCG: uncultured genus-level; UG: unclassified genus-level. Figure S5. Dose response effect of 3-nitrooxypropanol (3-NOP) supplementation on the abundance of A bacterial, B archaeal, and C protozoal taxa in dairy cattle. *3-NOP dose level information: con: 0, low: 68, high: 132 mg/kg of DM. Others indicates taxa with less than 5% abundance; UCG: uncultured genus-level; UG: unclassified genus-level. Figure S6. Alpha diversity and beta diversity analysis of rumen microbiota before and after batch correction. Alpha diversity was measured by Shannon index in A bacteria, B archaea, and C protozoa of control and 3-NOP treated groups. P values were calculat [file 40168_2025_2201_MOESM1_ESM.zip › Supplemental figures/Choi et al. FigureS13.pdf]

CPM

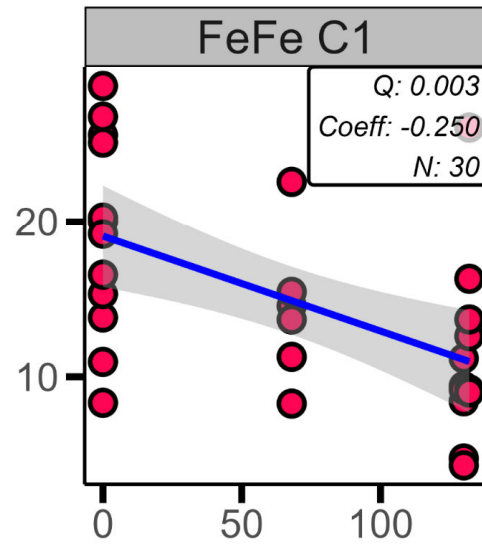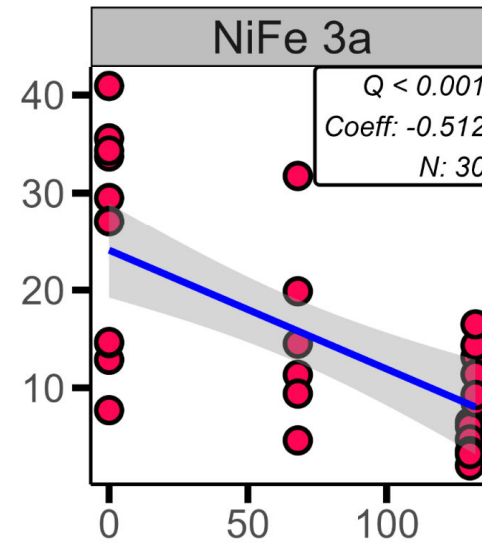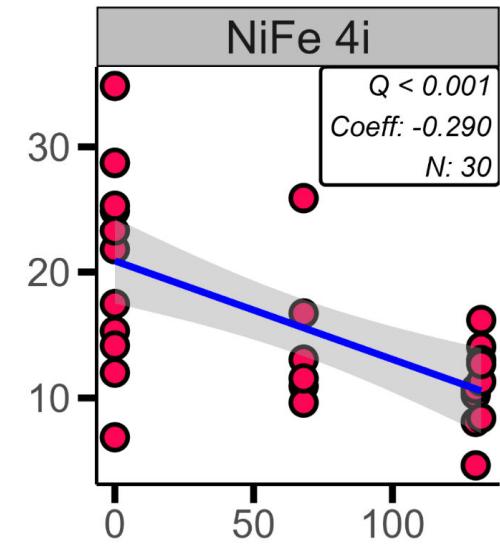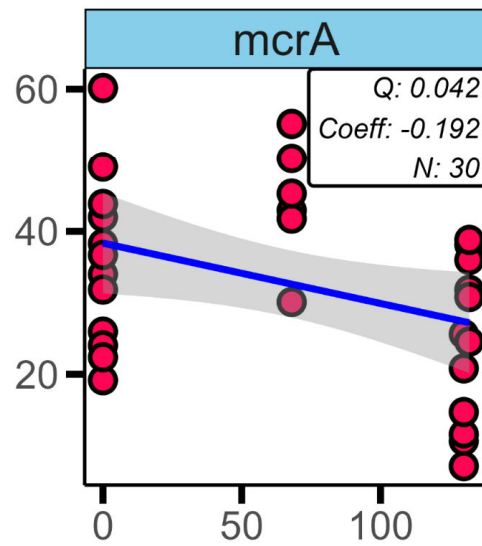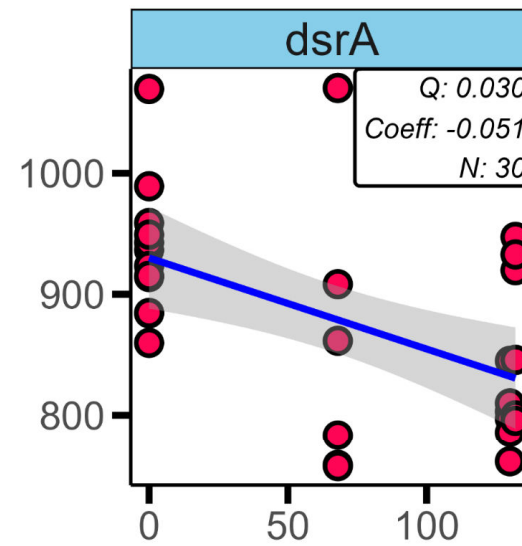

3-NOP dose (mg/kg of DM)

Supplement: Supplementary file 2 — Additional file 1: Figure S1. Schematic representation of the four in vivo trials used in the comparative analysis, including short-term and long-term 3-NOP supplementation studies in beef and dairy cattle (Beef1: Romero-Perez et al., 2014 [9]; Beef2: Romero-Perez et al., 2015 [10]; Dairy1: Haisan et al., 2014 [15]; Dairy2: Haisan et al., 2017 [16]). Figure S2. Effect of short-term 3-nitrooxypropanol (3-NOP) supplementation on the abundance of A bacterial, B archaeal, and C protozoal taxa in beef cattle. *3-NOP dose level information: con: 0, low: 53, med: 161, high: 345 mg/kg of DM. Others indicates taxa with less than 5% abundance; UCF: uncultured family-level; UCG: uncultured genus-level; UG: unclassified genus-level. Figure S3. Effect of long-term 3-nitrooxypropanol (3-NOP) supplementation on the abundance of A bacterial, B archaeal, and C protozoal taxa in beef cattle. *3-NOP dose level information: con: 0, high: 280 mg/kg of DM. Others indicates taxa with less than 5% abundance; UCG: uncultured genus-level; UG: unclassified genus-level; recov: recovery period. Figure S4. Effect of 3-nitrooxypropanol (3-NOP) supplementation on the abundance of A bacterial, B archaeal, and C protozoal taxa in dairy cattle. *3-NOP dose level information: con: 0, high: 130 mg/kg of DM. Others indicates taxa with less than 5% abundance; UCG: uncultured genus-level; UG: unclassified genus-level. Figure S5. Dose response effect of 3-nitrooxypropanol (3-NOP) supplementation on the abundance of A bacterial, B archaeal, and C protozoal taxa in dairy cattle. *3-NOP dose level information: con: 0, low: 68, high: 132 mg/kg of DM. Others indicates taxa with less than 5% abundance; UCG: uncultured genus-level; UG: unclassified genus-level. Figure S6. Alpha diversity and beta diversity analysis of rumen microbiota before and after batch correction. Alpha diversity was measured by Shannon index in A bacteria, B archaea, and C protozoa of control and 3-NOP treated groups. P values were calculat [file 40168_2025_2201_MOESM1_ESM.zip › Supplemental figures/Choi et al. FigureS14.pdf]

# Beef 1

## A Bacteria

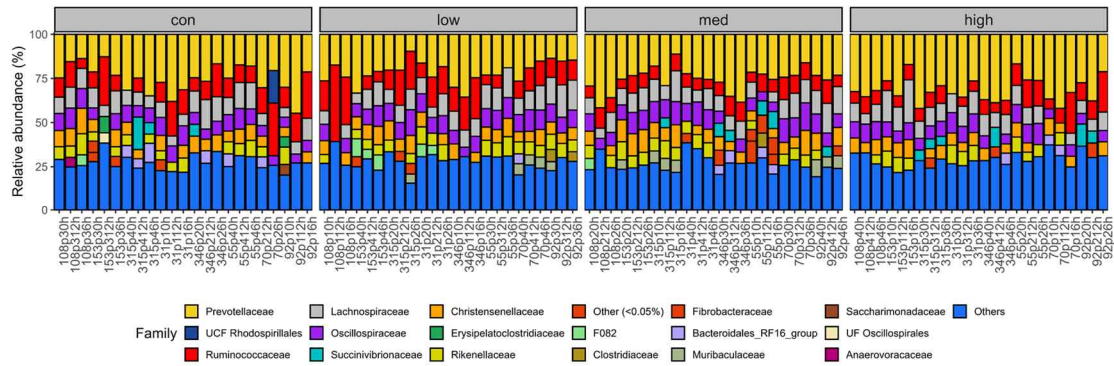

## B Archaea

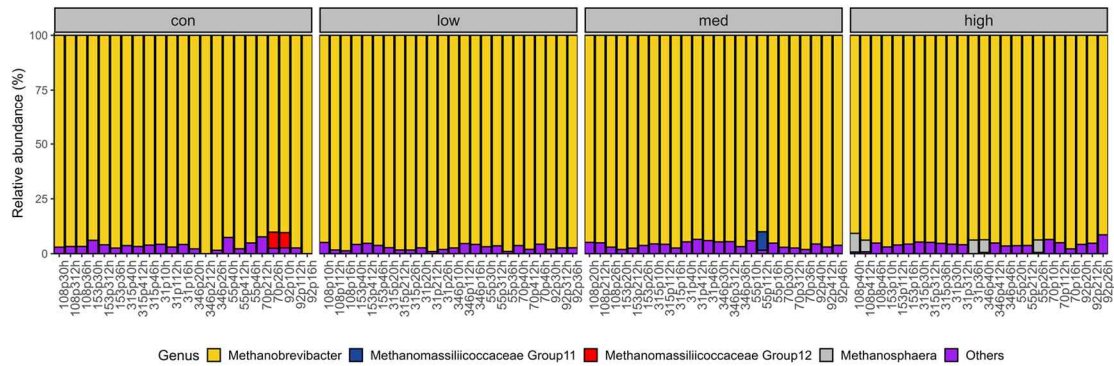

## C Protozoa

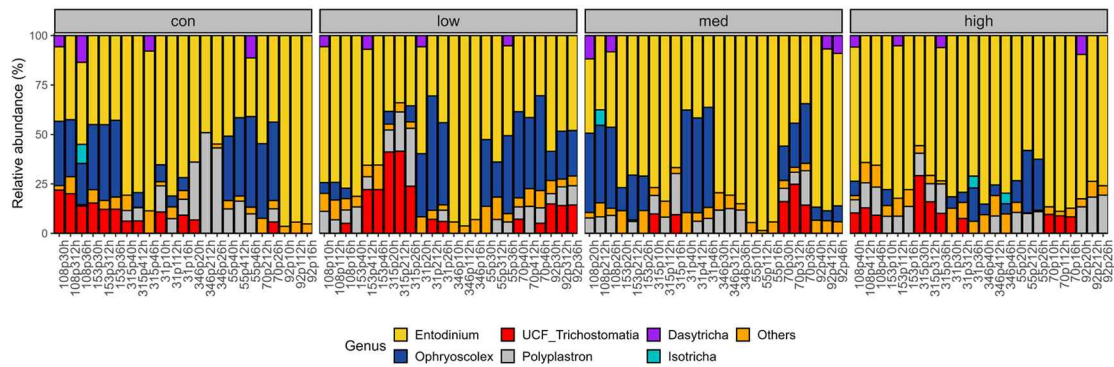

Supplement: Supplementary file 2 — Additional file 1: Figure S1. Schematic representation of the four in vivo trials used in the comparative analysis, including short-term and long-term 3-NOP supplementation studies in beef and dairy cattle (Beef1: Romero-Perez et al., 2014 [9]; Beef2: Romero-Perez et al., 2015 [10]; Dairy1: Haisan et al., 2014 [15]; Dairy2: Haisan et al., 2017 [16]). Figure S2. Effect of short-term 3-nitrooxypropanol (3-NOP) supplementation on the abundance of A bacterial, B archaeal, and C protozoal taxa in beef cattle. *3-NOP dose level information: con: 0, low: 53, med: 161, high: 345 mg/kg of DM. Others indicates taxa with less than 5% abundance; UCF: uncultured family-level; UCG: uncultured genus-level; UG: unclassified genus-level. Figure S3. Effect of long-term 3-nitrooxypropanol (3-NOP) supplementation on the abundance of A bacterial, B archaeal, and C protozoal taxa in beef cattle. *3-NOP dose level information: con: 0, high: 280 mg/kg of DM. Others indicates taxa with less than 5% abundance; UCG: uncultured genus-level; UG: unclassified genus-level; recov: recovery period. Figure S4. Effect of 3-nitrooxypropanol (3-NOP) supplementation on the abundance of A bacterial, B archaeal, and C protozoal taxa in dairy cattle. *3-NOP dose level information: con: 0, high: 130 mg/kg of DM. Others indicates taxa with less than 5% abundance; UCG: uncultured genus-level; UG: unclassified genus-level. Figure S5. Dose response effect of 3-nitrooxypropanol (3-NOP) supplementation on the abundance of A bacterial, B archaeal, and C protozoal taxa in dairy cattle. *3-NOP dose level information: con: 0, low: 68, high: 132 mg/kg of DM. Others indicates taxa with less than 5% abundance; UCG: uncultured genus-level; UG: unclassified genus-level. Figure S6. Alpha diversity and beta diversity analysis of rumen microbiota before and after batch correction. Alpha diversity was measured by Shannon index in A bacteria, B archaea, and C protozoa of control and 3-NOP treated groups. P values were calculat [file 40168_2025_2201_MOESM1_ESM.zip › Supplemental figures/Choi et al. FigureS2.pdf]

# Beef 2

## A Bacteria

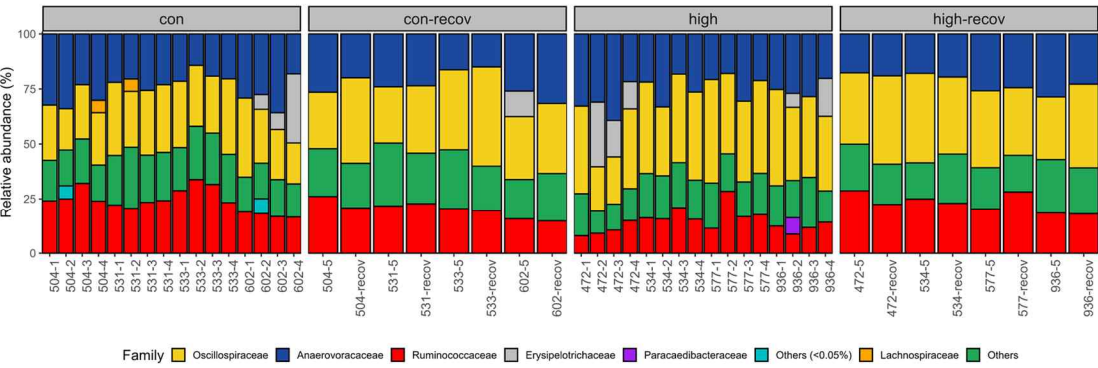

## B Archaea

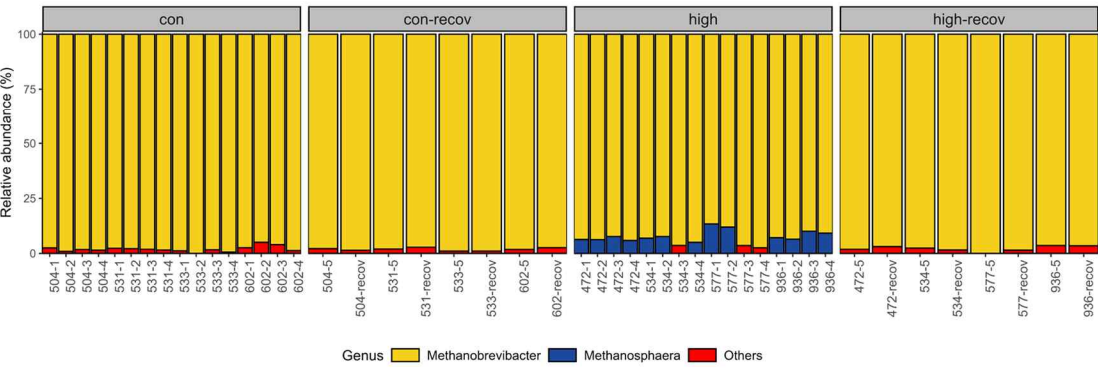

## C Protozoa

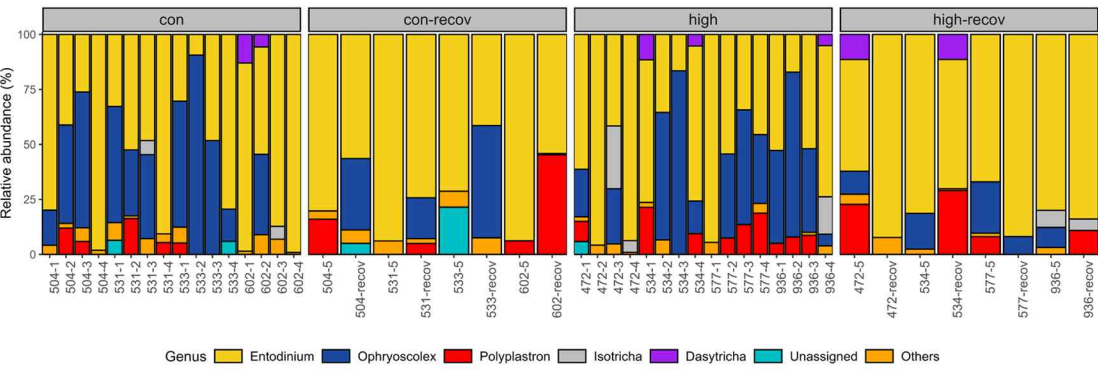

Supplement: Supplementary file 2 — Additional file 1: Figure S1. Schematic representation of the four in vivo trials used in the comparative analysis, including short-term and long-term 3-NOP supplementation studies in beef and dairy cattle (Beef1: Romero-Perez et al., 2014 [9]; Beef2: Romero-Perez et al., 2015 [10]; Dairy1: Haisan et al., 2014 [15]; Dairy2: Haisan et al., 2017 [16]). Figure S2. Effect of short-term 3-nitrooxypropanol (3-NOP) supplementation on the abundance of A bacterial, B archaeal, and C protozoal taxa in beef cattle. *3-NOP dose level information: con: 0, low: 53, med: 161, high: 345 mg/kg of DM. Others indicates taxa with less than 5% abundance; UCF: uncultured family-level; UCG: uncultured genus-level; UG: unclassified genus-level. Figure S3. Effect of long-term 3-nitrooxypropanol (3-NOP) supplementation on the abundance of A bacterial, B archaeal, and C protozoal taxa in beef cattle. *3-NOP dose level information: con: 0, high: 280 mg/kg of DM. Others indicates taxa with less than 5% abundance; UCG: uncultured genus-level; UG: unclassified genus-level; recov: recovery period. Figure S4. Effect of 3-nitrooxypropanol (3-NOP) supplementation on the abundance of A bacterial, B archaeal, and C protozoal taxa in dairy cattle. *3-NOP dose level information: con: 0, high: 130 mg/kg of DM. Others indicates taxa with less than 5% abundance; UCG: uncultured genus-level; UG: unclassified genus-level. Figure S5. Dose response effect of 3-nitrooxypropanol (3-NOP) supplementation on the abundance of A bacterial, B archaeal, and C protozoal taxa in dairy cattle. *3-NOP dose level information: con: 0, low: 68, high: 132 mg/kg of DM. Others indicates taxa with less than 5% abundance; UCG: uncultured genus-level; UG: unclassified genus-level. Figure S6. Alpha diversity and beta diversity analysis of rumen microbiota before and after batch correction. Alpha diversity was measured by Shannon index in A bacteria, B archaea, and C protozoa of control and 3-NOP treated groups. P values were calculat [file 40168_2025_2201_MOESM1_ESM.zip › Supplemental figures/Choi et al. FigureS3.pdf]

# Dairy 1

## A Bacteria

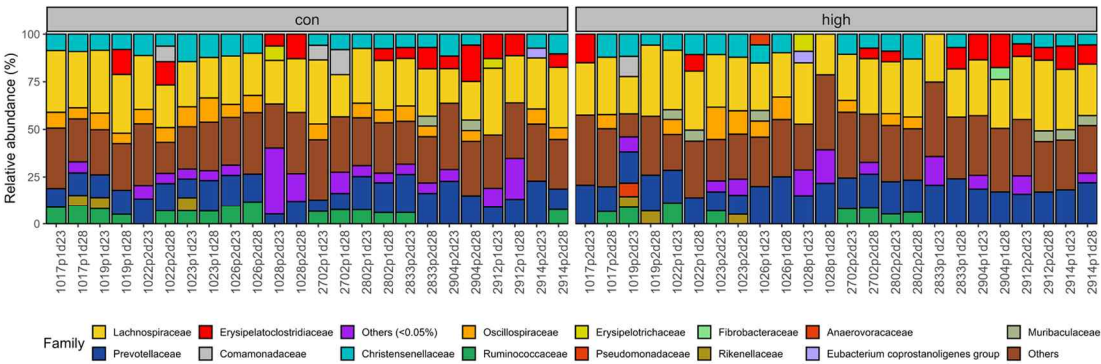

## B Archaea

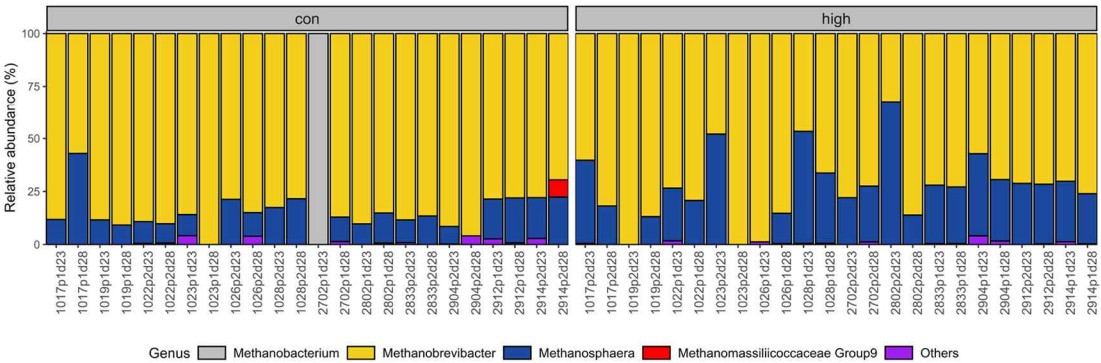

## C Protozoa

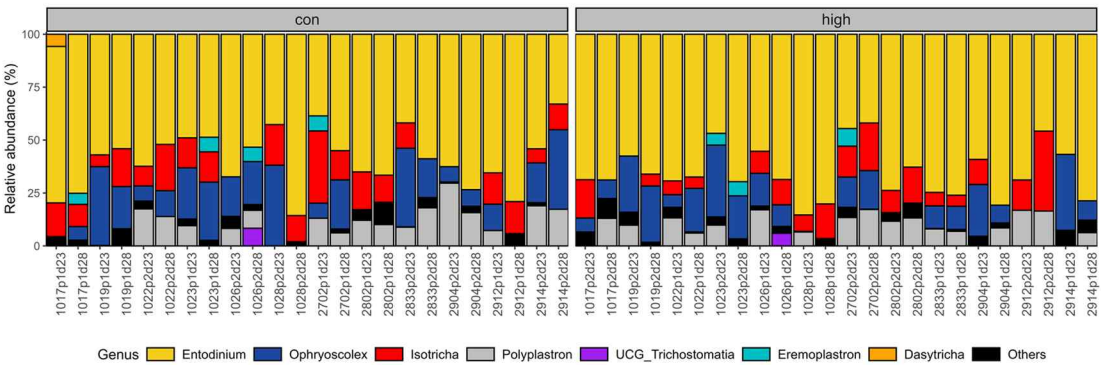

Supplement: Supplementary file 2 — Additional file 1: Figure S1. Schematic representation of the four in vivo trials used in the comparative analysis, including short-term and long-term 3-NOP supplementation studies in beef and dairy cattle (Beef1: Romero-Perez et al., 2014 [9]; Beef2: Romero-Perez et al., 2015 [10]; Dairy1: Haisan et al., 2014 [15]; Dairy2: Haisan et al., 2017 [16]). Figure S2. Effect of short-term 3-nitrooxypropanol (3-NOP) supplementation on the abundance of A bacterial, B archaeal, and C protozoal taxa in beef cattle. *3-NOP dose level information: con: 0, low: 53, med: 161, high: 345 mg/kg of DM. Others indicates taxa with less than 5% abundance; UCF: uncultured family-level; UCG: uncultured genus-level; UG: unclassified genus-level. Figure S3. Effect of long-term 3-nitrooxypropanol (3-NOP) supplementation on the abundance of A bacterial, B archaeal, and C protozoal taxa in beef cattle. *3-NOP dose level information: con: 0, high: 280 mg/kg of DM. Others indicates taxa with less than 5% abundance; UCG: uncultured genus-level; UG: unclassified genus-level; recov: recovery period. Figure S4. Effect of 3-nitrooxypropanol (3-NOP) supplementation on the abundance of A bacterial, B archaeal, and C protozoal taxa in dairy cattle. *3-NOP dose level information: con: 0, high: 130 mg/kg of DM. Others indicates taxa with less than 5% abundance; UCG: uncultured genus-level; UG: unclassified genus-level. Figure S5. Dose response effect of 3-nitrooxypropanol (3-NOP) supplementation on the abundance of A bacterial, B archaeal, and C protozoal taxa in dairy cattle. *3-NOP dose level information: con: 0, low: 68, high: 132 mg/kg of DM. Others indicates taxa with less than 5% abundance; UCG: uncultured genus-level; UG: unclassified genus-level. Figure S6. Alpha diversity and beta diversity analysis of rumen microbiota before and after batch correction. Alpha diversity was measured by Shannon index in A bacteria, B archaea, and C protozoa of control and 3-NOP treated groups. P values were calculat [file 40168_2025_2201_MOESM1_ESM.zip › Supplemental figures/Choi et al. FigureS4.pdf]

# Dairy 2

## A Bacteria

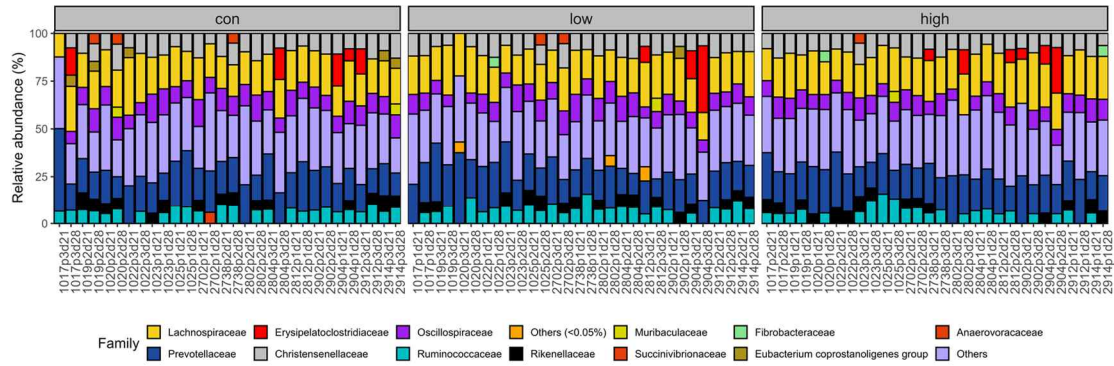

## B Archaea

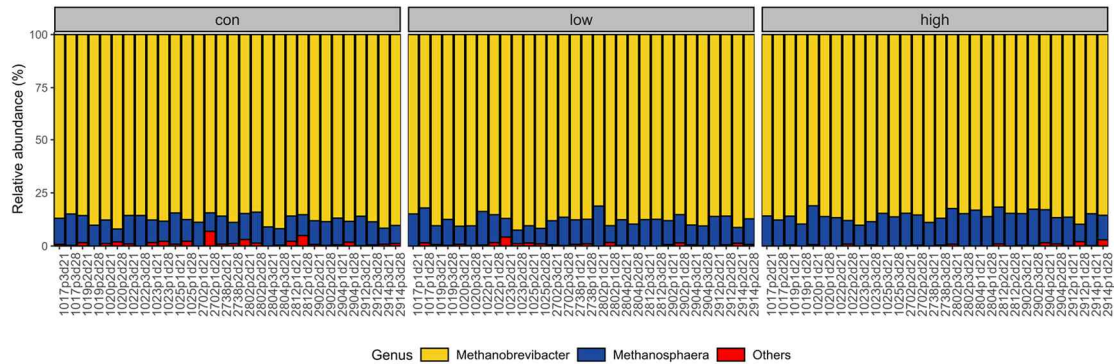

## C Protozoa

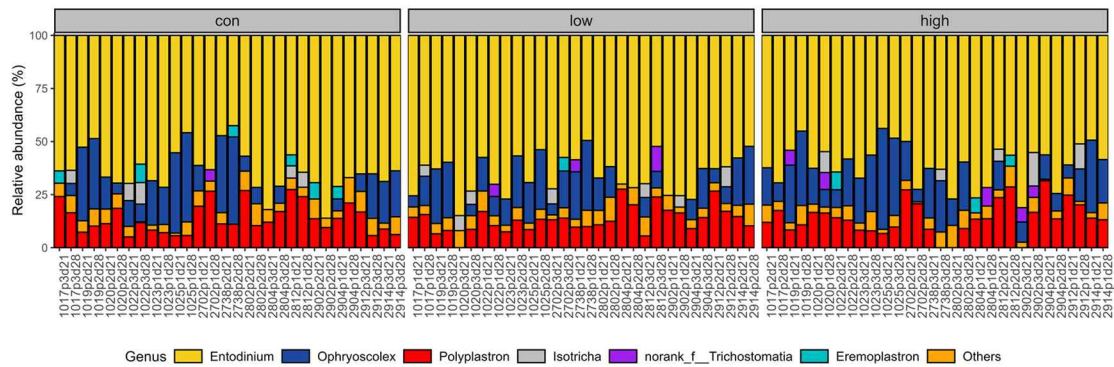

Supplement: Supplementary file 2 — Additional file 1: Figure S1. Schematic representation of the four in vivo trials used in the comparative analysis, including short-term and long-term 3-NOP supplementation studies in beef and dairy cattle (Beef1: Romero-Perez et al., 2014 [9]; Beef2: Romero-Perez et al., 2015 [10]; Dairy1: Haisan et al., 2014 [15]; Dairy2: Haisan et al., 2017 [16]). Figure S2. Effect of short-term 3-nitrooxypropanol (3-NOP) supplementation on the abundance of A bacterial, B archaeal, and C protozoal taxa in beef cattle. *3-NOP dose level information: con: 0, low: 53, med: 161, high: 345 mg/kg of DM. Others indicates taxa with less than 5% abundance; UCF: uncultured family-level; UCG: uncultured genus-level; UG: unclassified genus-level. Figure S3. Effect of long-term 3-nitrooxypropanol (3-NOP) supplementation on the abundance of A bacterial, B archaeal, and C protozoal taxa in beef cattle. *3-NOP dose level information: con: 0, high: 280 mg/kg of DM. Others indicates taxa with less than 5% abundance; UCG: uncultured genus-level; UG: unclassified genus-level; recov: recovery period. Figure S4. Effect of 3-nitrooxypropanol (3-NOP) supplementation on the abundance of A bacterial, B archaeal, and C protozoal taxa in dairy cattle. *3-NOP dose level information: con: 0, high: 130 mg/kg of DM. Others indicates taxa with less than 5% abundance; UCG: uncultured genus-level; UG: unclassified genus-level. Figure S5. Dose response effect of 3-nitrooxypropanol (3-NOP) supplementation on the abundance of A bacterial, B archaeal, and C protozoal taxa in dairy cattle. *3-NOP dose level information: con: 0, low: 68, high: 132 mg/kg of DM. Others indicates taxa with less than 5% abundance; UCG: uncultured genus-level; UG: unclassified genus-level. Figure S6. Alpha diversity and beta diversity analysis of rumen microbiota before and after batch correction. Alpha diversity was measured by Shannon index in A bacteria, B archaea, and C protozoa of control and 3-NOP treated groups. P values were calculat [file 40168_2025_2201_MOESM1_ESM.zip › Supplemental figures/Choi et al. FigureS5.pdf]

**A**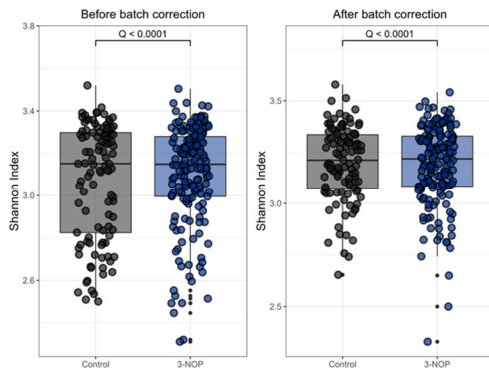**B**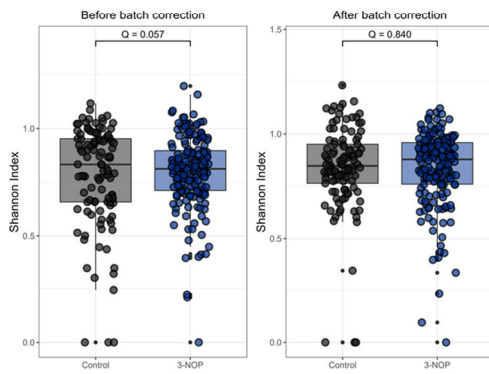**C**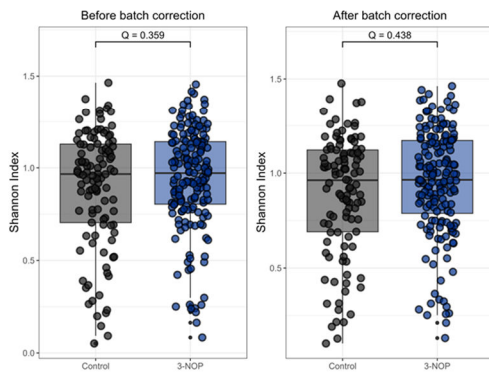**D**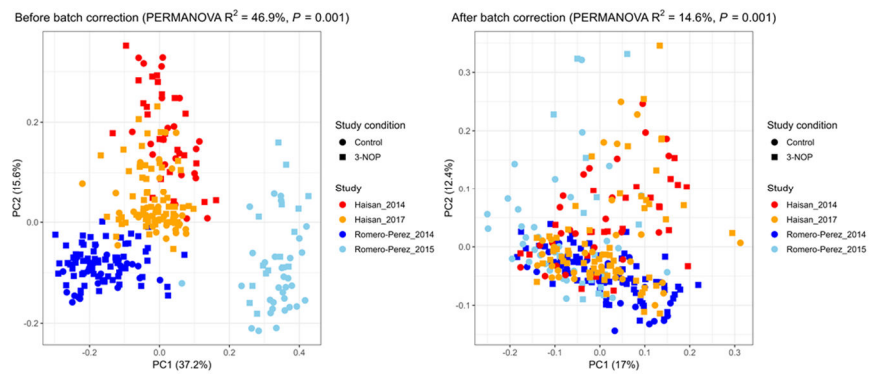**E**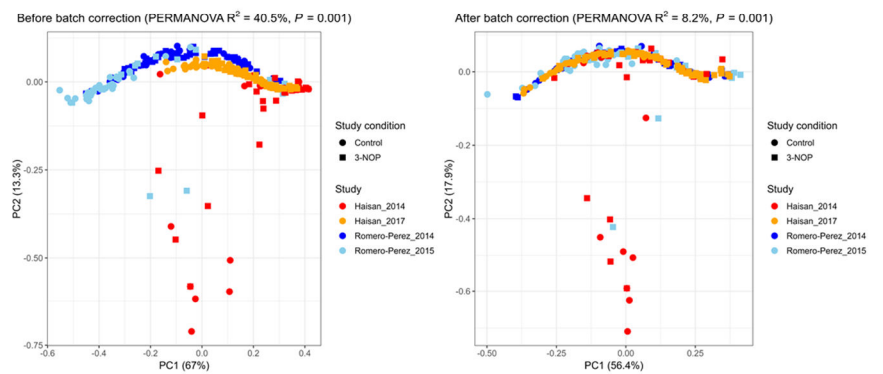**F**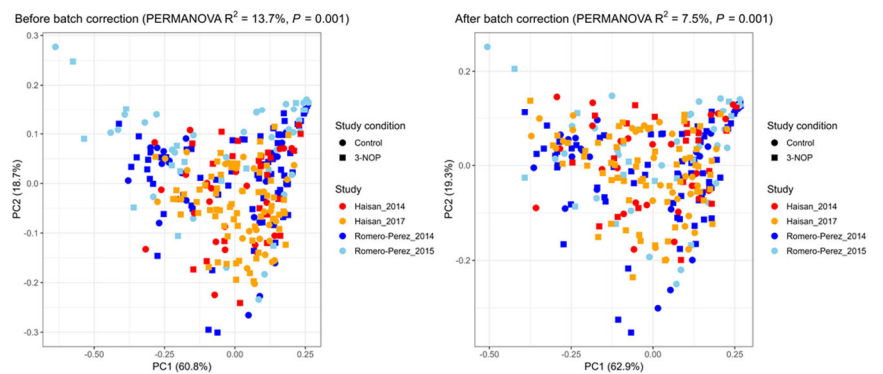

Supplement: Supplementary file 2 — Additional file 1: Figure S1. Schematic representation of the four in vivo trials used in the comparative analysis, including short-term and long-term 3-NOP supplementation studies in beef and dairy cattle (Beef1: Romero-Perez et al., 2014 [9]; Beef2: Romero-Perez et al., 2015 [10]; Dairy1: Haisan et al., 2014 [15]; Dairy2: Haisan et al., 2017 [16]). Figure S2. Effect of short-term 3-nitrooxypropanol (3-NOP) supplementation on the abundance of A bacterial, B archaeal, and C protozoal taxa in beef cattle. *3-NOP dose level information: con: 0, low: 53, med: 161, high: 345 mg/kg of DM. Others indicates taxa with less than 5% abundance; UCF: uncultured family-level; UCG: uncultured genus-level; UG: unclassified genus-level. Figure S3. Effect of long-term 3-nitrooxypropanol (3-NOP) supplementation on the abundance of A bacterial, B archaeal, and C protozoal taxa in beef cattle. *3-NOP dose level information: con: 0, high: 280 mg/kg of DM. Others indicates taxa with less than 5% abundance; UCG: uncultured genus-level; UG: unclassified genus-level; recov: recovery period. Figure S4. Effect of 3-nitrooxypropanol (3-NOP) supplementation on the abundance of A bacterial, B archaeal, and C protozoal taxa in dairy cattle. *3-NOP dose level information: con: 0, high: 130 mg/kg of DM. Others indicates taxa with less than 5% abundance; UCG: uncultured genus-level; UG: unclassified genus-level. Figure S5. Dose response effect of 3-nitrooxypropanol (3-NOP) supplementation on the abundance of A bacterial, B archaeal, and C protozoal taxa in dairy cattle. *3-NOP dose level information: con: 0, low: 68, high: 132 mg/kg of DM. Others indicates taxa with less than 5% abundance; UCG: uncultured genus-level; UG: unclassified genus-level. Figure S6. Alpha diversity and beta diversity analysis of rumen microbiota before and after batch correction. Alpha diversity was measured by Shannon index in A bacteria, B archaea, and C protozoa of control and 3-NOP treated groups. P values were calculat [file 40168_2025_2201_MOESM1_ESM.zip › Supplemental figures/Choi et al. FigureS6.pdf]

## A Beef 1

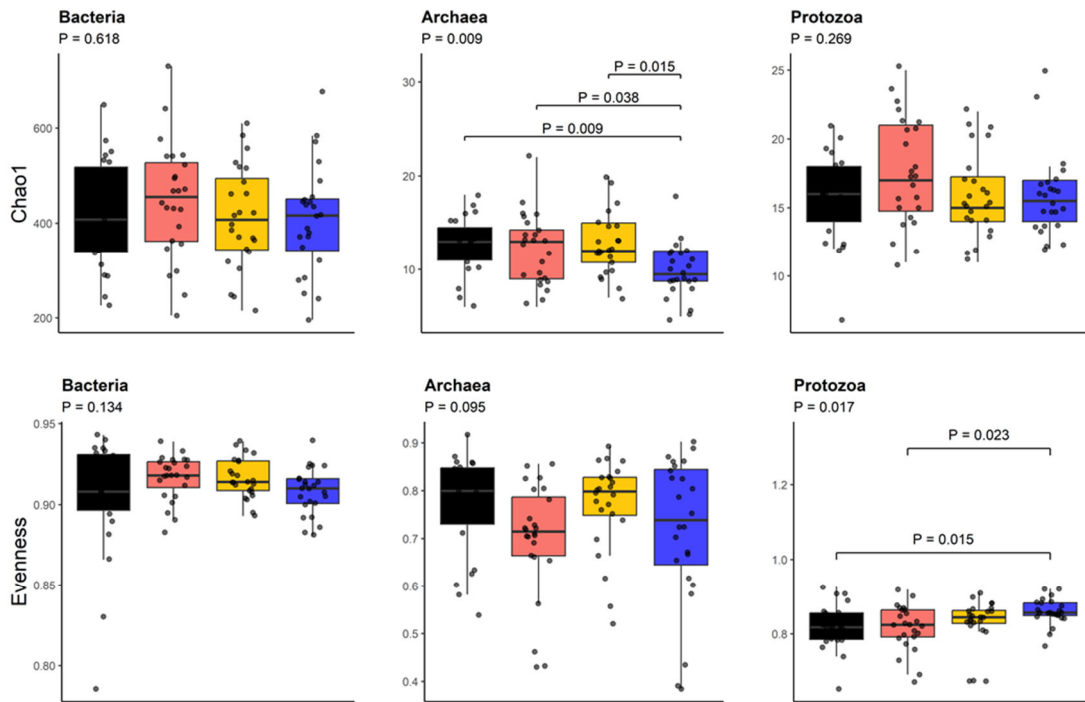

## B Beef 2

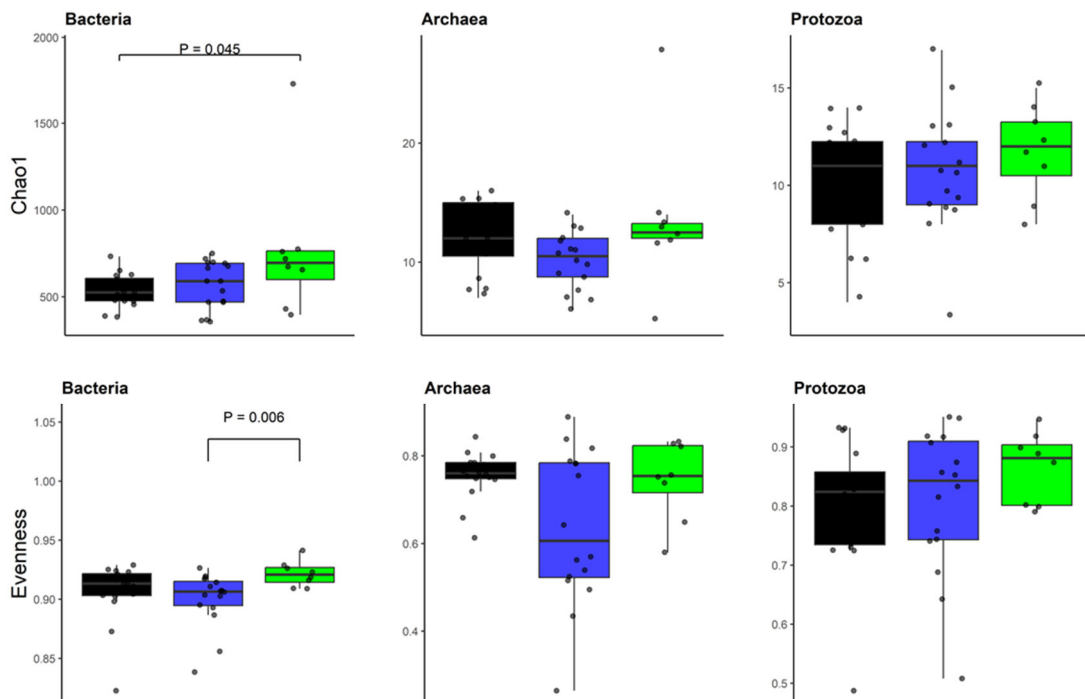

Supplement: Supplementary file 2 — Additional file 1: Figure S1. Schematic representation of the four in vivo trials used in the comparative analysis, including short-term and long-term 3-NOP supplementation studies in beef and dairy cattle (Beef1: Romero-Perez et al., 2014 [9]; Beef2: Romero-Perez et al., 2015 [10]; Dairy1: Haisan et al., 2014 [15]; Dairy2: Haisan et al., 2017 [16]). Figure S2. Effect of short-term 3-nitrooxypropanol (3-NOP) supplementation on the abundance of A bacterial, B archaeal, and C protozoal taxa in beef cattle. *3-NOP dose level information: con: 0, low: 53, med: 161, high: 345 mg/kg of DM. Others indicates taxa with less than 5% abundance; UCF: uncultured family-level; UCG: uncultured genus-level; UG: unclassified genus-level. Figure S3. Effect of long-term 3-nitrooxypropanol (3-NOP) supplementation on the abundance of A bacterial, B archaeal, and C protozoal taxa in beef cattle. *3-NOP dose level information: con: 0, high: 280 mg/kg of DM. Others indicates taxa with less than 5% abundance; UCG: uncultured genus-level; UG: unclassified genus-level; recov: recovery period. Figure S4. Effect of 3-nitrooxypropanol (3-NOP) supplementation on the abundance of A bacterial, B archaeal, and C protozoal taxa in dairy cattle. *3-NOP dose level information: con: 0, high: 130 mg/kg of DM. Others indicates taxa with less than 5% abundance; UCG: uncultured genus-level; UG: unclassified genus-level. Figure S5. Dose response effect of 3-nitrooxypropanol (3-NOP) supplementation on the abundance of A bacterial, B archaeal, and C protozoal taxa in dairy cattle. *3-NOP dose level information: con: 0, low: 68, high: 132 mg/kg of DM. Others indicates taxa with less than 5% abundance; UCG: uncultured genus-level; UG: unclassified genus-level. Figure S6. Alpha diversity and beta diversity analysis of rumen microbiota before and after batch correction. Alpha diversity was measured by Shannon index in A bacteria, B archaea, and C protozoa of control and 3-NOP treated groups. P values were calculat [file 40168_2025_2201_MOESM1_ESM.zip › Supplemental figures/Choi et al. FigureS7a.pdf]

## C Dairy 1

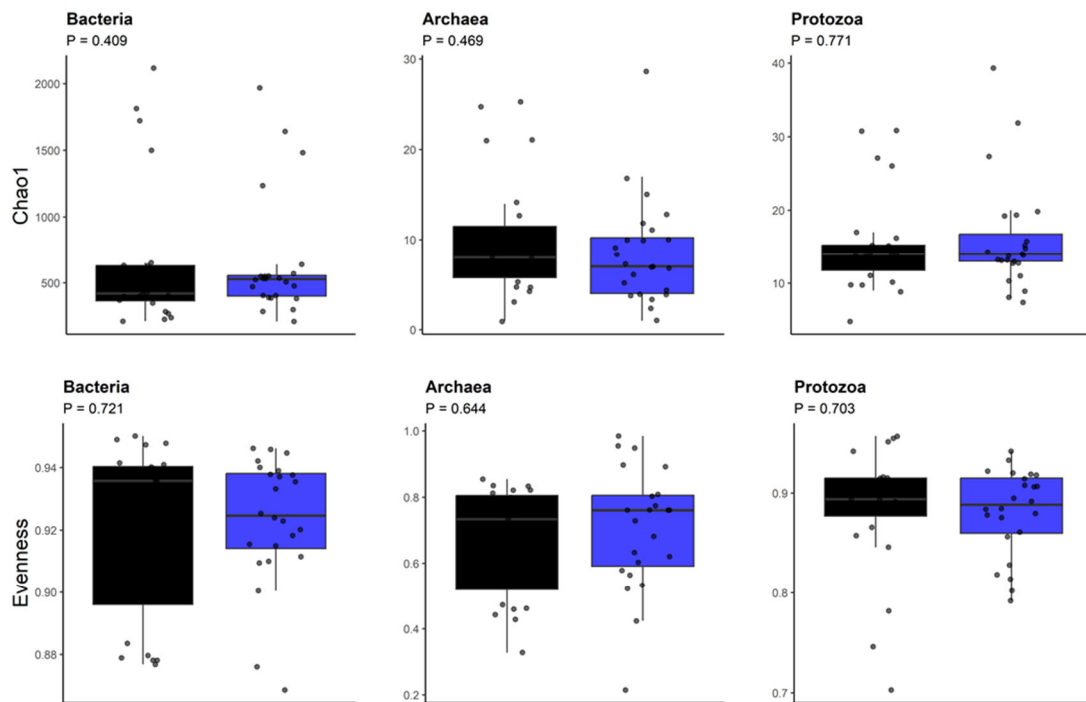

## D Dairy 2

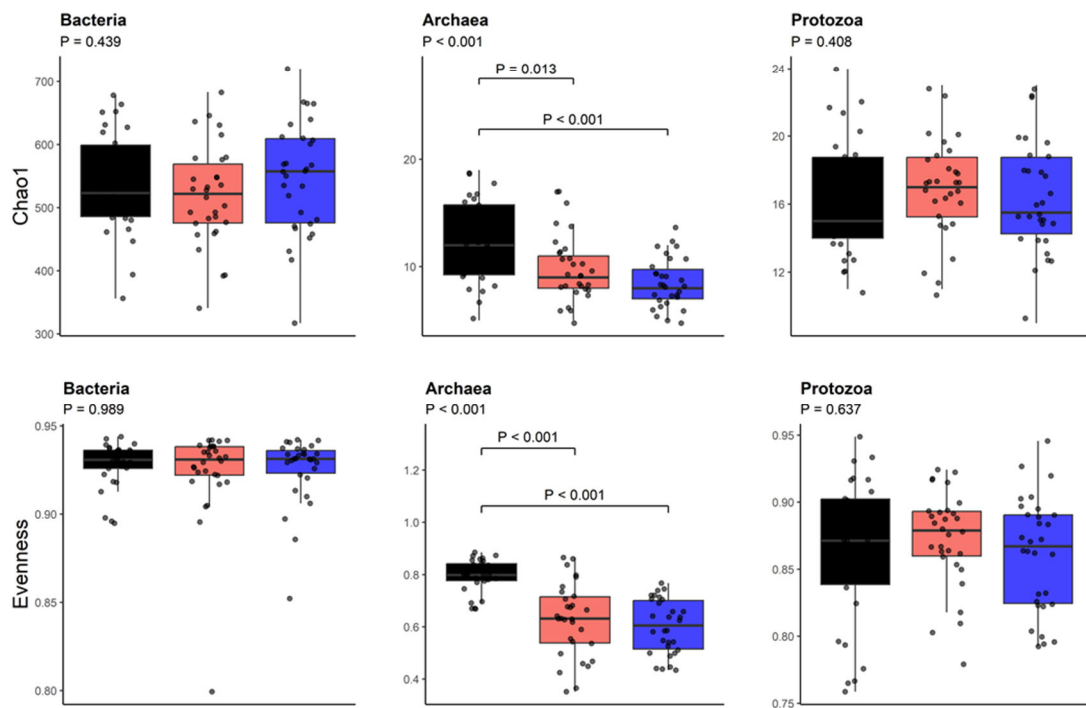

Supplement: Supplementary file 2 — Additional file 1: Figure S1. Schematic representation of the four in vivo trials used in the comparative analysis, including short-term and long-term 3-NOP supplementation studies in beef and dairy cattle (Beef1: Romero-Perez et al., 2014 [9]; Beef2: Romero-Perez et al., 2015 [10]; Dairy1: Haisan et al., 2014 [15]; Dairy2: Haisan et al., 2017 [16]). Figure S2. Effect of short-term 3-nitrooxypropanol (3-NOP) supplementation on the abundance of A bacterial, B archaeal, and C protozoal taxa in beef cattle. *3-NOP dose level information: con: 0, low: 53, med: 161, high: 345 mg/kg of DM. Others indicates taxa with less than 5% abundance; UCF: uncultured family-level; UCG: uncultured genus-level; UG: unclassified genus-level. Figure S3. Effect of long-term 3-nitrooxypropanol (3-NOP) supplementation on the abundance of A bacterial, B archaeal, and C protozoal taxa in beef cattle. *3-NOP dose level information: con: 0, high: 280 mg/kg of DM. Others indicates taxa with less than 5% abundance; UCG: uncultured genus-level; UG: unclassified genus-level; recov: recovery period. Figure S4. Effect of 3-nitrooxypropanol (3-NOP) supplementation on the abundance of A bacterial, B archaeal, and C protozoal taxa in dairy cattle. *3-NOP dose level information: con: 0, high: 130 mg/kg of DM. Others indicates taxa with less than 5% abundance; UCG: uncultured genus-level; UG: unclassified genus-level. Figure S5. Dose response effect of 3-nitrooxypropanol (3-NOP) supplementation on the abundance of A bacterial, B archaeal, and C protozoal taxa in dairy cattle. *3-NOP dose level information: con: 0, low: 68, high: 132 mg/kg of DM. Others indicates taxa with less than 5% abundance; UCG: uncultured genus-level; UG: unclassified genus-level. Figure S6. Alpha diversity and beta diversity analysis of rumen microbiota before and after batch correction. Alpha diversity was measured by Shannon index in A bacteria, B archaea, and C protozoa of control and 3-NOP treated groups. P values were calculat [file 40168_2025_2201_MOESM1_ESM.zip › Supplemental figures/Choi et al. FigureS7b.pdf]

A Beef 1

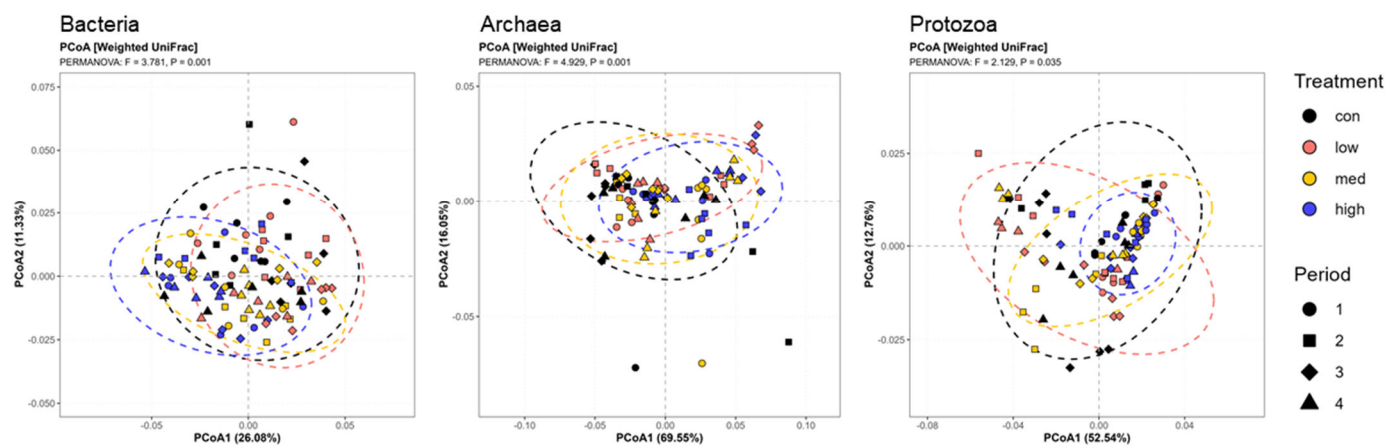

B Beef 2

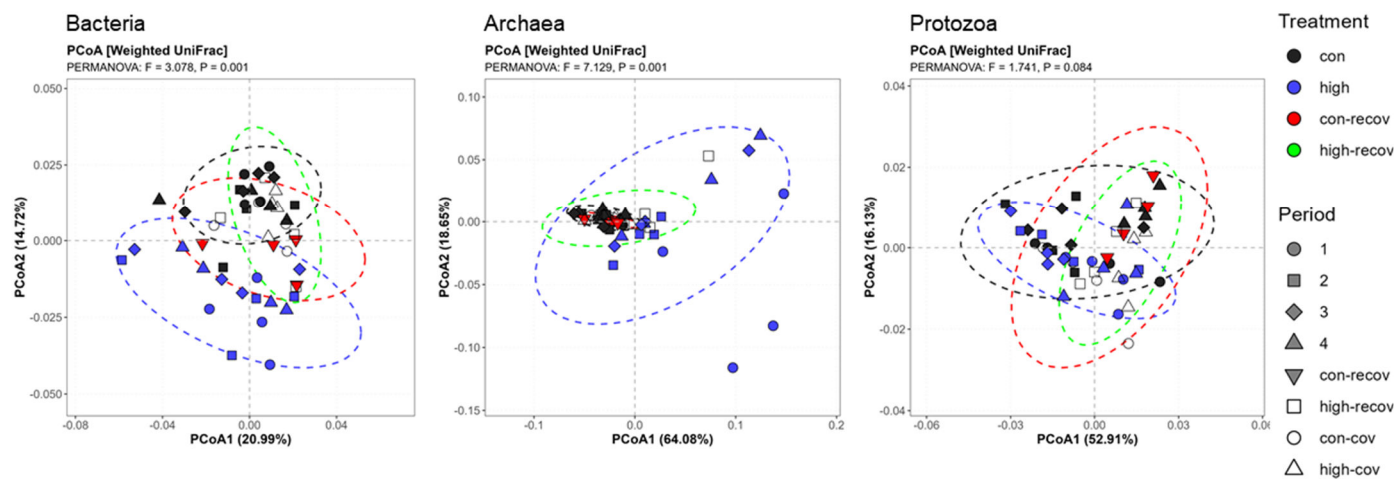

Supplement: Supplementary file 2 — Additional file 1: Figure S1. Schematic representation of the four in vivo trials used in the comparative analysis, including short-term and long-term 3-NOP supplementation studies in beef and dairy cattle (Beef1: Romero-Perez et al., 2014 [9]; Beef2: Romero-Perez et al., 2015 [10]; Dairy1: Haisan et al., 2014 [15]; Dairy2: Haisan et al., 2017 [16]). Figure S2. Effect of short-term 3-nitrooxypropanol (3-NOP) supplementation on the abundance of A bacterial, B archaeal, and C protozoal taxa in beef cattle. *3-NOP dose level information: con: 0, low: 53, med: 161, high: 345 mg/kg of DM. Others indicates taxa with less than 5% abundance; UCF: uncultured family-level; UCG: uncultured genus-level; UG: unclassified genus-level. Figure S3. Effect of long-term 3-nitrooxypropanol (3-NOP) supplementation on the abundance of A bacterial, B archaeal, and C protozoal taxa in beef cattle. *3-NOP dose level information: con: 0, high: 280 mg/kg of DM. Others indicates taxa with less than 5% abundance; UCG: uncultured genus-level; UG: unclassified genus-level; recov: recovery period. Figure S4. Effect of 3-nitrooxypropanol (3-NOP) supplementation on the abundance of A bacterial, B archaeal, and C protozoal taxa in dairy cattle. *3-NOP dose level information: con: 0, high: 130 mg/kg of DM. Others indicates taxa with less than 5% abundance; UCG: uncultured genus-level; UG: unclassified genus-level. Figure S5. Dose response effect of 3-nitrooxypropanol (3-NOP) supplementation on the abundance of A bacterial, B archaeal, and C protozoal taxa in dairy cattle. *3-NOP dose level information: con: 0, low: 68, high: 132 mg/kg of DM. Others indicates taxa with less than 5% abundance; UCG: uncultured genus-level; UG: unclassified genus-level. Figure S6. Alpha diversity and beta diversity analysis of rumen microbiota before and after batch correction. Alpha diversity was measured by Shannon index in A bacteria, B archaea, and C protozoa of control and 3-NOP treated groups. P values were calculat [file 40168_2025_2201_MOESM1_ESM.zip › Supplemental figures/Choi et al. FigureS8a.pdf]

## C Dairy 1

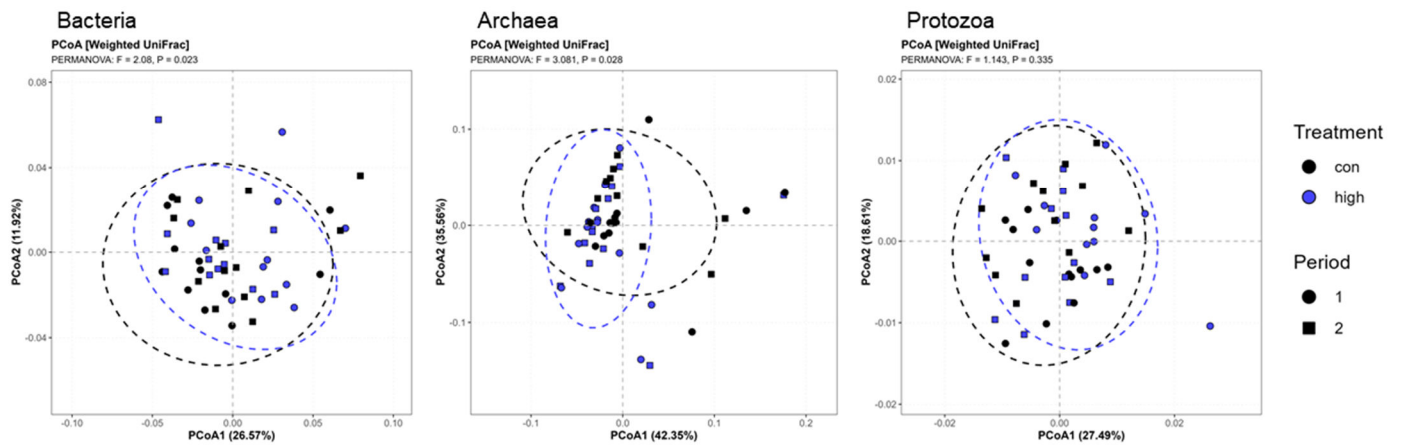

## D Dairy 2

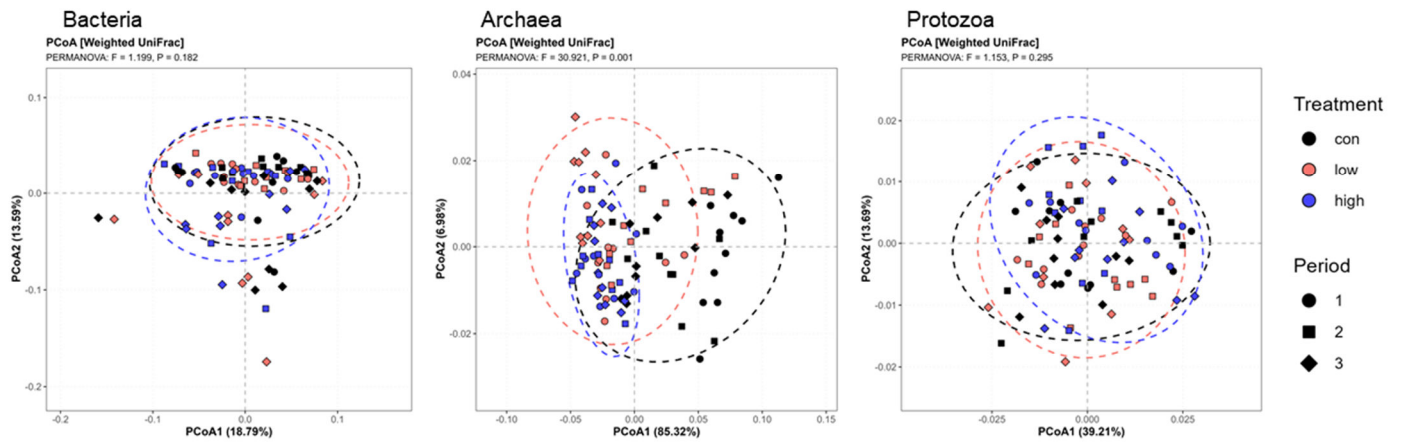

Supplement: Supplementary file 2 — Additional file 1: Figure S1. Schematic representation of the four in vivo trials used in the comparative analysis, including short-term and long-term 3-NOP supplementation studies in beef and dairy cattle (Beef1: Romero-Perez et al., 2014 [9]; Beef2: Romero-Perez et al., 2015 [10]; Dairy1: Haisan et al., 2014 [15]; Dairy2: Haisan et al., 2017 [16]). Figure S2. Effect of short-term 3-nitrooxypropanol (3-NOP) supplementation on the abundance of A bacterial, B archaeal, and C protozoal taxa in beef cattle. *3-NOP dose level information: con: 0, low: 53, med: 161, high: 345 mg/kg of DM. Others indicates taxa with less than 5% abundance; UCF: uncultured family-level; UCG: uncultured genus-level; UG: unclassified genus-level. Figure S3. Effect of long-term 3-nitrooxypropanol (3-NOP) supplementation on the abundance of A bacterial, B archaeal, and C protozoal taxa in beef cattle. *3-NOP dose level information: con: 0, high: 280 mg/kg of DM. Others indicates taxa with less than 5% abundance; UCG: uncultured genus-level; UG: unclassified genus-level; recov: recovery period. Figure S4. Effect of 3-nitrooxypropanol (3-NOP) supplementation on the abundance of A bacterial, B archaeal, and C protozoal taxa in dairy cattle. *3-NOP dose level information: con: 0, high: 130 mg/kg of DM. Others indicates taxa with less than 5% abundance; UCG: uncultured genus-level; UG: unclassified genus-level. Figure S5. Dose response effect of 3-nitrooxypropanol (3-NOP) supplementation on the abundance of A bacterial, B archaeal, and C protozoal taxa in dairy cattle. *3-NOP dose level information: con: 0, low: 68, high: 132 mg/kg of DM. Others indicates taxa with less than 5% abundance; UCG: uncultured genus-level; UG: unclassified genus-level. Figure S6. Alpha diversity and beta diversity analysis of rumen microbiota before and after batch correction. Alpha diversity was measured by Shannon index in A bacteria, B archaea, and C protozoa of control and 3-NOP treated groups. P values were calculat [file 40168_2025_2201_MOESM1_ESM.zip › Supplemental figures/Choi et al. FigureS8b.pdf]

Beef 1

A Hydrogenase

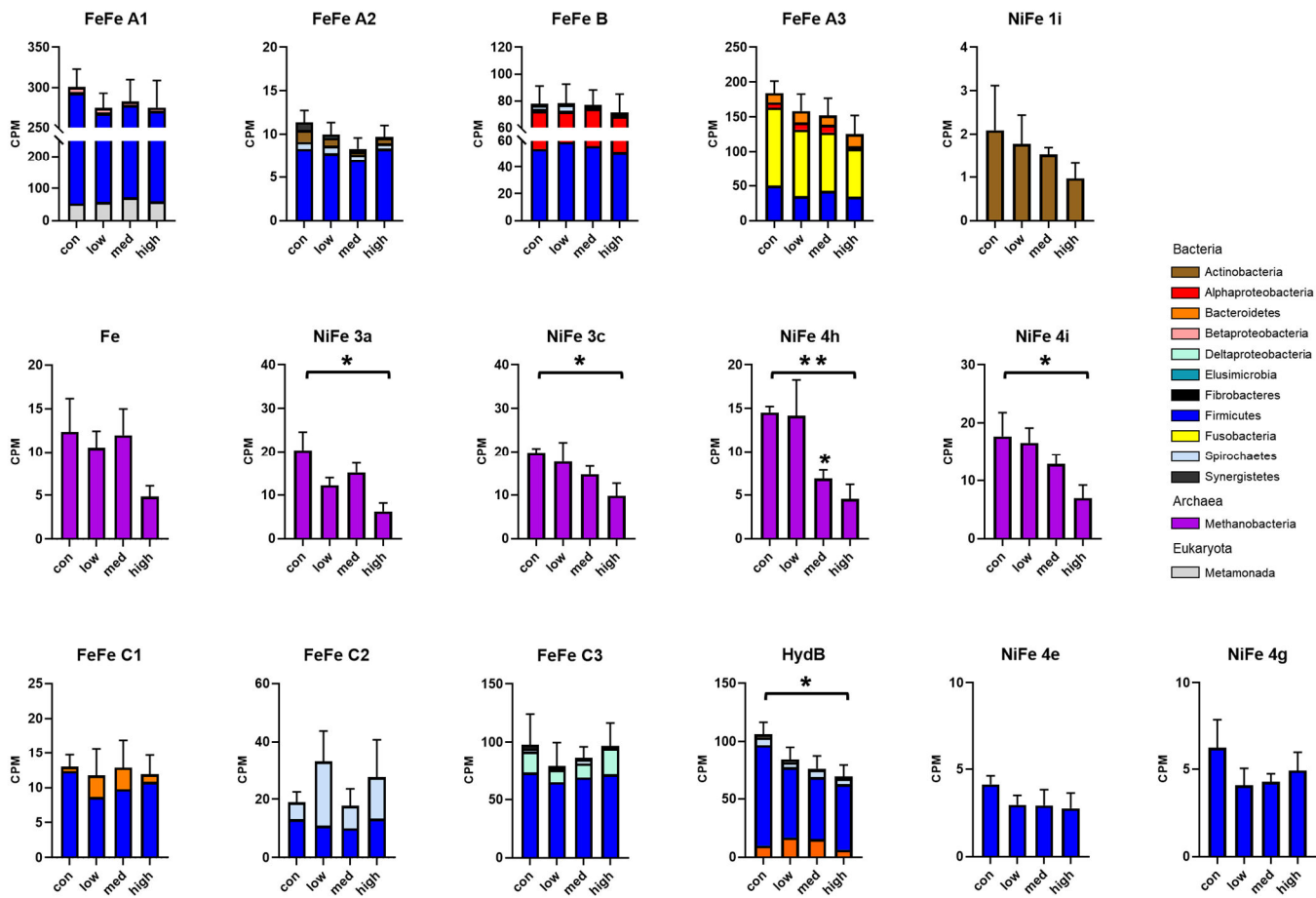

B Terminal reductase

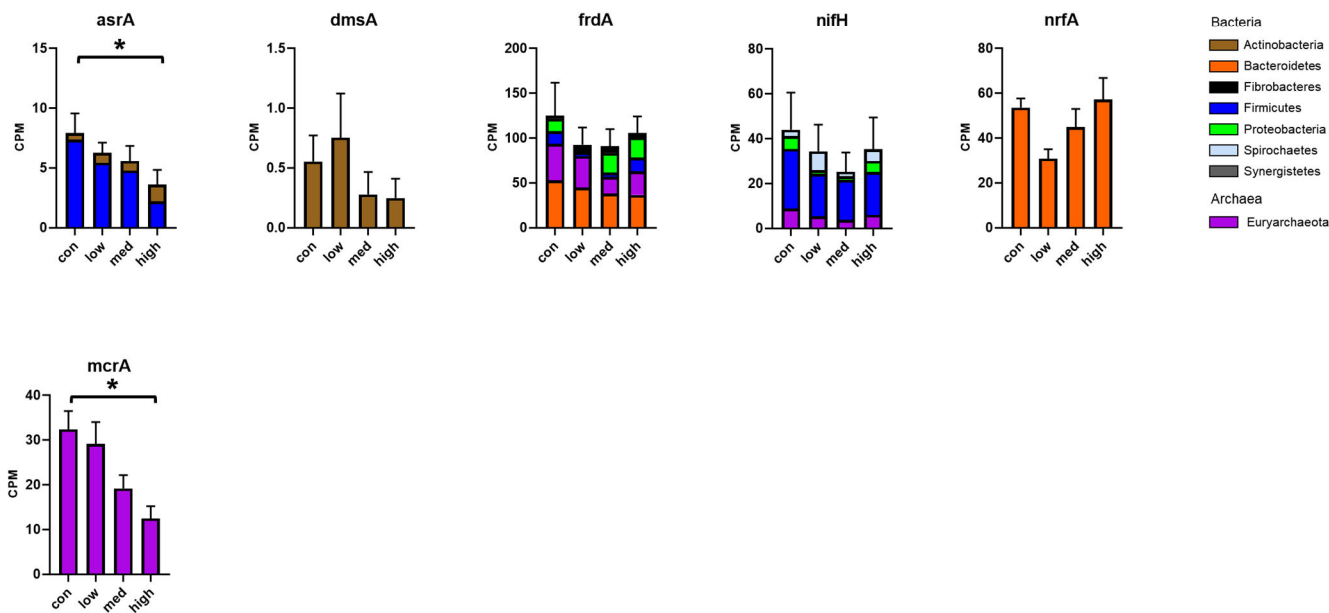

Supplement: Supplementary file 2 — Additional file 1: Figure S1. Schematic representation of the four in vivo trials used in the comparative analysis, including short-term and long-term 3-NOP supplementation studies in beef and dairy cattle (Beef1: Romero-Perez et al., 2014 [9]; Beef2: Romero-Perez et al., 2015 [10]; Dairy1: Haisan et al., 2014 [15]; Dairy2: Haisan et al., 2017 [16]). Figure S2. Effect of short-term 3-nitrooxypropanol (3-NOP) supplementation on the abundance of A bacterial, B archaeal, and C protozoal taxa in beef cattle. *3-NOP dose level information: con: 0, low: 53, med: 161, high: 345 mg/kg of DM. Others indicates taxa with less than 5% abundance; UCF: uncultured family-level; UCG: uncultured genus-level; UG: unclassified genus-level. Figure S3. Effect of long-term 3-nitrooxypropanol (3-NOP) supplementation on the abundance of A bacterial, B archaeal, and C protozoal taxa in beef cattle. *3-NOP dose level information: con: 0, high: 280 mg/kg of DM. Others indicates taxa with less than 5% abundance; UCG: uncultured genus-level; UG: unclassified genus-level; recov: recovery period. Figure S4. Effect of 3-nitrooxypropanol (3-NOP) supplementation on the abundance of A bacterial, B archaeal, and C protozoal taxa in dairy cattle. *3-NOP dose level information: con: 0, high: 130 mg/kg of DM. Others indicates taxa with less than 5% abundance; UCG: uncultured genus-level; UG: unclassified genus-level. Figure S5. Dose response effect of 3-nitrooxypropanol (3-NOP) supplementation on the abundance of A bacterial, B archaeal, and C protozoal taxa in dairy cattle. *3-NOP dose level information: con: 0, low: 68, high: 132 mg/kg of DM. Others indicates taxa with less than 5% abundance; UCG: uncultured genus-level; UG: unclassified genus-level. Figure S6. Alpha diversity and beta diversity analysis of rumen microbiota before and after batch correction. Alpha diversity was measured by Shannon index in A bacteria, B archaea, and C protozoa of control and 3-NOP treated groups. P values were calculat [file 40168_2025_2201_MOESM1_ESM.zip › Supplemental figures/Choi et al. FigureS9.pdf]
