# Supplementary material for: Comparative analysis of rumen metagenomes with dietary supplementation of 3-nitrooxypropanol revealed divergent modes of action in hydrogen metabolism and reductant pathways between beef and dairy cattle
Source: Microbiome. 2026 Feb 19;14:72. doi: 10.1186/s40168-025-02201-y (PMC12918512; doi:10.1186/s40168-025-02201-y)
Supplement: Supplementary file 3 — Additional file 2: Table S1. Ingredient and chemical composition of the basal diet in beef study 1. Table S2. Ingredient and chemical composition of the basal diet in beef study 2. Table S3. Ingredient and chemical composition of the basal diet in dairy study 1. Table S4. Ingredient and chemical composition of the basal diet in dairy study 2. [file 40168_2025_2201_MOESM2_ESM.docx]

**Tables**

**Table S1.** Ingredient and chemical composition of the basal diet in beef study 1*

| Item | % of DM |
| --- | --- |
| Ingredient^1,2^ |  |
| Barley silage^3^ | 60 |
| Barley grain, dry rolled | 35 |
| Barley grain, ground | 2.688 |
| Calcium carbonate | 1.374 |
| Canola meal | 0.500 |
| Salt | 0.158 |
| Urea | 0.110 |
| Molasses, dried | 0.108 |
| LRC feedlot vitamin-mineral premix^4^ | 0.055 |
| Vitamin E (500,000 IU/kg) | 0.004 |
| Flavoring agent | 0.003 |
| Chemical composition^5^ |  |
| DM | 46.7 ± 2.05 |
| OM, % of DM | 92.9 ± 0.43 |
| CP, % of DM | 11.7 ± 0.25 |
| NDF, % of DM | 37.6 ± 1.31 |
| ADF, % of DM | 20.6 ± 0.55 |
| Starch, % of DM | 31.8 ± 1.03 |
| Fat, % of DM | 2.7 ± 0.19 |
| Ash, | 7.1 ± 0.43 |
| NFC^6^ | 40.9 |

^1^All ingredients except barley silage and dry-rolled barley grain were provided as part of a pelleted supplement.

^2^Each beef received 2.69 mg/d of melengesterol acetate as a pellet to suppress estrous activity. Pellet contained MGA-100 premix (Pfizer Canada Inc., Kirkland, QC, Canada), 0.45%; ground barley grain, 95.99%; dried molasses, 2.51%; and flavoring agent, 0.05% (DM basis). It was fed at 600 g/animal daily (as-is basis).

^3^Composition: DM, 34.5%; CP, 11.4%; NDF, 49.7%; ADF, 32.3%; starch, 16.1%; and GE, 5.6 Mcal/kg.

^4^Feedlot vitamin-mineral premix contained CaCO_3_, 35.01%; CuSO_4_, 10.37%; ZnSO_4_, 28.23%; Ethylenediamine dihydriodide (80% concentration), 0.15%; selenium 1% (10,000 mg Se/kg), 5.01%; CoSO4, 0.1%; MnSO_4_, 14.54%; vitamin A (500,000,000 IU/kg), 1.71%; vitamin D (500,000,000 IU/ kg), 0.17%; and vitamin E (500,000 IU/kg), 4.7%.

^5^Mean ± SD; n = 4

^6^NFC (non-fibrous carbohydrate) was calculated by NFC (%) = 100 - (NDF + CP + Fat + Ash).

*This table was reproduced from Romero-Pérez et al. [9].

**Table S2.** Ingredient and chemical composition of the basal diet in beef study 2*

| Item | % of DM |
| --- | --- |
| Ingredient^1^ |  |
| Barley silage^2^ | 60 |
| Barley grain, dry rolled | 35 |
| Barley grain, ground | 2.688 |
| Calcium carbonate | 1.374 |
| Canola meal | 0.500 |
| Salt | 0.158 |
| Urea | 0.110 |
| Molasses, dried | 0.108 |
| LRC feedlot vitamin-mineral premix^3^ | 0.055 |
| Vitamin E (500,000 IU/kg) | 0.004 |
| Flavoring agent | 0.003 |
| Chemical composition^4^ |  |
| DM | 53.6 ± 2.18 |
| OM, % of DM | 92.5 ± 0.24 |
| CP, % of DM | 11.3 ± 0.63 |
| NDF, % of DM | 38.6 ± 1.35 |
| ADF, % of DM | 22.4 ± 1.72 |
| Starch, % of DM | 33.8 ± 0.57 |
| Fat, % of DM | 1.85 ± 0.13 |
| Ash | 7.5 ± 0.24 |
| NFC^5^ | 40.8 |
| GE, Mcal/kg | 4.9 ± 0.53 |

^1^All ingredients except barley silage and dry-rolled barley grain were provided as part of a pelleted supplement.

^2^Composition: 42.4% DM, 10.5% CP, 49.9% NDF, and 33.1% ADF.

^3^Feedlot vitamin–mineral premix contained 35.01% CaCO_3_, 10.37% CuSO_4_, 28.23% ZnSO_4_, 0.15% ethylenediamine dihydriodide (80% concentration), 5.01% selenium 1% (10,000 mg Se/kg), 0.1% CoSO_4_, 14.54% MnSO_4_, 1.71% vitamin A (500,000,000 IU/kg), 0.17% vitamin D (500,000,000 IU/kg), and 4.7% vitamin E (500,000 IU/kg).

^4^Mean ± SD; n = 6

^5^NFC (non-fibrous carbohydrate) was calculated by NFC (%) = 100 - (NDF + CP + Fat + Ash).

*This table was reproduced from Romero-Pérez et al. [10].

**Table S3.** Ingredient and chemical composition of the basal diet in dairy study 1*

| Item (% of DM) | Total |
| --- | --- |
| Ingredient |  |
| Barley silage | 37.9 |
| Dry ground corn | 31.4 |
| Canola meal | 11.3 |
| Corn gluten meal | 8.4 |
| Beet pulp | 6.2 |
| Canola oil | 1.4 |
| Limestone | 1.2 |
| Salt | 1.2 |
| Calcium diphosphate | 0.6 |
| Magnesium oxide | 0.3 |
| Mineral and vitamin premix^1^ | 0.1 |
| Chemical composition |  |
| DM, % | 53.5 |
| OM | 90.8 |
| CP | 19.6 |
| NDF | 26.5 |
| ADF | 18.4 |
| Starch | 26.8 |
| Ether extracts | 5.0 |
| Ash | 9.2 |
| NFC^2^ | 39.7 |

^1^Contained 17,413 KIU/kg of vitamin A, 1,714 KIU/kg of vitamin D3, 57 KIU/kg of vitamin E, 579 mg/kg of Co, 28,586 mg/kg of Cu, 1,286 mg/kg of I, 51,429 mg/kg of Mn, 85,714 mg/kg of Zn, and 571 mg/kg of Se.

^2^NFC (non-fibrous carbohydrate) was calculated by NFC (%) = 100 - (NDF + CP + Fat + Ash).

*This table was reproduced from Haisan et al. [17].

**Table S4.** Ingredient and chemical composition of the basal diet in dairy study 2*

| Item (% of DM) | Total |
| --- | --- |
| Ingredient |  |
| Barley silage | 60.0 |
| Dry ground corn | 20.2 |
| Canola meal | 7.3 |
| Corn gluten meal | 5.4 |
| Beet pulp | 4.0 |
| Canola oil | 0.9 |
| Limestone | 0.8 |
| Salt | 0.8 |
| Calcium diphosphate | 0.4 |
| Magnesium oxide | 0.2 |
| Mineral and vitamin premix^1^ | 0.1 |
| Chemical composition |  |
| DM, % | 46.1 |
| OM | 90.7 |
| CP | 19.6 |
| NDF | 33.8 |
| Ether extracts | 3.4 |
| Ash | 9.3 |
| NFC^2^ | 33.9 |

^1^Contained 17 413 KIU/kg vitamin A, 1714 KIU/kg vitamin D3, 57 KIU/kg vitamin E, 579 mg/kg Co, 28 586 mg/kg Cu, 1286 mg/kg I, 51 429 mg/kg Mn, 85714 mg/kg Zn, and 571 mg/kg Se.

^2^NFC (non-fibrous carbohydrate) was calculated by NFC (%) = 100 - (NDF + CP + Fat + Ash).

*This table was reproduced from Haisan et al. [18].
